# Jackfruit genome and population genomics provide insights into fruit evolution and domestication history in China

**DOI:** 10.1093/hr/uhac173

**Published:** 2022-08-04

**Authors:** Xinggu Lin, Chao Feng, Tao Lin, A J Harris, Yingzhi Li, Ming Kang

**Affiliations:** Key Laboratory of Plant Resources Conservation and Sustainable Utilization, South China Botanical Garden, Chinese Academy of Sciences, 510650 Guangzhou, China; University of Chinese Academy of Sciences, 100049 Beijing, China; Key Laboratory of Plant Resources Conservation and Sustainable Utilization, South China Botanical Garden, Chinese Academy of Sciences, 510650 Guangzhou, China; Key Laboratory of Plant Resources Conservation and Sustainable Utilization, South China Botanical Garden, Chinese Academy of Sciences, 510650 Guangzhou, China; University of Chinese Academy of Sciences, 100049 Beijing, China; Key Laboratory of Plant Resources Conservation and Sustainable Utilization, South China Botanical Garden, Chinese Academy of Sciences, 510650 Guangzhou, China; Horticulture and Forestry Department, Guangdong Ocean University, 524088 Zhanjiang, China; Key Laboratory of Plant Resources Conservation and Sustainable Utilization, South China Botanical Garden, Chinese Academy of Sciences, 510650 Guangzhou, China; Center of Conservation Biology, Core Botanical Gardens, Chinese Academy of Sciences, 510650 Guangzhou, China

## Abstract

As the largest known tree-borne fruit in the world, jackfruit (*Artocarpus heterophyllus*) is an important cultivated crop in tropical regions of South and Southeast Asia. The species has been cultivated in China for more than 1000 years, but the history of its introduction to the country remains unclear. We assembled a high-quality chromosome-level genome of jackfruit into 985.63 Mb with scaffold N50 of 32.81 Mb. We analyzed whole-genome resequencing data of 295 landraces to investigate the domestication history in China and agronomic trait evolution of jackfruit. Population structure analysis revealed that jackfruits of China could be traced back to originate from Southeast Asia and South Asia independently. Selection signals between jackfruit and its edible congener, cempedak (*Artocarpus integer*), revealed several important candidate genes associated with fruit development and ripening. Moreover, analyses of selective sweeps and gene expression revealed that the *AhePG1* gene may be the major factor in determining fruit texture. This study not only resolves the origins of jackfruit of China, but also provides valuable genomic resources for jackfruit breeding improvement and offers insights into fruit size evolution and fruit texture changes.

## Introduction

The genus *Artocarpus* comprises ~70 species of evergreen flowering trees in the mulberry family (Moraceae) and is widely distributed from South and Southeast Asia into Oceania [1]. It includes several economically important fruit crops, such as jackfruit (*A. heterophyllus*), breadfruit (*A. altilis*), breadnut (*A. camansi*), cempedak (*A. integer*), and marang (*A. odoratissimus*). Of them, the jackfruit, *A. heterophyllus*, is native to South and Southeast Asia but has been introduced throughout the tropics as a multipurpose tree, providing food, fodder, timber, fuel, and medicinal products [2]. Jackfruit is the largest known tree-borne fruit in the world, with one fruit weighing up to 45 kg and having hundreds of seeds. The greenish unripe fruit may be cooked as a vegetable or prepared with sauces as a vegetarian substitute for meat, while the brown ripened fruit is eaten fresh for the sweet pulp surrounding the seeds. The fruit is a rich source of carbohydrates, proteins, potassium, calcium, iron, and vitamins [2]. The starchy seeds are extremely high in vitamin B1 and B2 and are also edible when cooked [3]. Moreover, the jackfruit has antioxidant, anti-inflammatory, antibacterial, antineoplastic and hypoglycemic effects because it contains many classes of phytochemicals such as carotenoids, flavanoids, volatile acids, sterols, and tannins [2].

Despite its important nutritional value and medicinal properties, jackfruit is still considered a minor fruit crop in much of eastern Asia. However, it is an important crop in India and other Southeast Asian countries and is, consequently, the national fruit of Bangladesh and Sri Lanka. Jackfruit is known as the poor man’s fruit, because it is easy to grow and is resilient to pests and diseases, which makes it a perfect candidate to improve food and nutrition security as well as to raise income. In Bangladesh, jackfruit is the second most important fruit crop after mango, representing ~27% of total fruit production of the country [4]. Globally, there has been increasing interest in promoting and commercializing jackfruit products in recent decades due to the rising awareness of the importance of improving food security. According to the statistics of the United Nations Food and Agriculture Organization (FAO), the production of jackfruit was estimated at 3.7 million tons over the 3-year span of 2015–17 [5].

Jackfruit plays a significant role in the agriculture and culture of India, where it has been cultivated for at least 3000 years [6]. However, the geographic origin of jackfruit remains enigmatic, and no clear wild ancestor has been identified [7]. Today, one widely accepted hypothesis suggests that jackfruit originated in the Western Ghats of South India and thereafter was spread as a crop throughout Southeast and East Asia, tropical Africa, and the Americas [8, 9]. Alternatively, Barrau posited that it originated in Malaysia due to the great diversity of jackfruit cultivars found there [10]. However, jackfruit has never been found in the wild in Malaysia and therefore it was likely introduced there from other regions [8]. Nevertheless, recent work suggests that Borneo is a center of diversification for the genus and *A. integer* (cempedak, an important crop in Malaysia), which is sister to *A. heterophyllus*, is native to Borneo and the Thai-Malay Peninsula [11]. If jackfruit originated in the Malay region, it may have reached the Western Ghats via overland dispersal through Indo-Burma [11].

In China, jackfruit has been cultivated for >1000 years and is now widely grown from Yunnan in southwest China to Fujian in eastern China [12]. However, its origin and introduction history remain unclear. According to literary and historical records [12], an envoy of the Pala Dynasty (now Bangladesh and Northeast India) brought jackfruit seeds to China and planted them in Guangzhou >1500 years ago. In contrast, some historians recorded that jackfruit was introduced from Southeast Asian countries during the 11th to 17th centuries and was first cultivated in Hainan Island [12]. In addition, jackfruit was probably also introduced to Taiwan Island in the 17th century by the Dutch, with subsequent spread to the mainland. However, these hypotheses about the origin of jackfruit in China have not been tested within a molecular phylogenomic framework.

Jackfruit trees are primarily cultivated in southern China in home gardens or on small farms, where they comprise many phenotypically diverse landraces. The trees are an out-crossing, seed-propagated species with few or no highly stable cultivars. Despite evidence that jackfruit has been cultivated for millennia, this species has not experienced intensive human selection. For these reasons, jackfruit exhibits a wide range of genetic and phenotypic diversity in fruit size, shape, color, flesh type and sugar content, flowering time, and other agronomic traits. Previous studies have provided insights into the genetic diversity of jackfruit germplasm in China and other countries from South and Southeast Asia, but they used only a small number of markers and accessions [7, 13, 14]. Recent progress in next-generation sequencing (NGS) has significantly advanced genomic studies in fruit crops [15, 16]. In particular, whole-genome resequencing techniques enable the high-throughput development of single-nucleotide polymorphisms (SNPs) to detect and characterize genomic regions associated with agronomic traits [17, 18]. Thus, NGS approaches can be used to quickly and efficiently expand the genomic resources for jackfruits and enhance our abilities to understand the genetic basis of its economically important phenotypes under domestication.

Here, we first assembled the chromosome-level genome of jackfruit by combining third-generation PacBio sequencing technology with a high-density genetic linkage map. Thereafter, we conducted whole-genome resequencing on 295 accessions, principally landraces of *A. heterophyllus* from China, Bangladesh, Thailand, Malaysia, and Indonesia. To provide context in our genomic analyses, we also included 12 individuals of *A. integer* and 22 individuals of other species from the same genus. We used these genomic data to determine patterns of genetic diversity and population structure and to infer jackfruit domestication history in China. We also performed scans for selection between the genomes of *A. heterophyllus* and *A. integer* to identify candidate regions associated with changes in fruit size during jackfruit domestication. Moreover, we carried out a selective sweep analysis to dissect the genomic basis of fruit texture of jackfruit. Our work provides the first insights into the genome-wide patterns of diversity and domestication history of jackfruit, and adds to the understanding of genetic loci that may affect traits associated with fruit quality. The jackfruit genome with the linkage map provides a valuable resource for molecular breeding of this tropical fruit.

## Results

### Chromosome-level genome assembly and annotation

We sequenced the genome of jackfruit (a firm-flesh type, cultivar ‘S10’) yielding a 985.85-Mb assembly, covering 92.44% of the estimated genome based on Illumina paired-end reads (total data 97.88 Gb, ~92.34× coverage), PacBio single-molecule long reads (total data 111.85 Gb, ~105.52× coverage), and 10× Genomics (total data 116.84 Gb, ~110.23× coverage) (Supplementary Data Fig. S1; Supplementary Data Tables S1–S3). In order to anchor the assembled jackfruit genome to chromosomes, we constructed a *de novo* genetic map of the *F*_1_ population of a cross between a firm-flesh type and a soft-flesh type. This map comprised 2834 SNP markers across 28 linkage groups and spanned 4386.39 cM with a mean marker distance of 1.91 cM (Supplementary Data Fig. S2; Supplementary Data Table S4). Subsequently, we mapped the assembled jackfruit genome with the high-density genetic linkage map to construct the chromosomes and order the scaffolds based on the genetic distance (Supplementary Data Table S5). The final genome assembly consisted of 482 scaffolds with N50 sizes of 32.81 Mb, spanned 985.63 Mb, and covered 92.98% of the estimated genome (Supplementary Data Table S6). The high conformance and completeness of the assembled genome were confirmed by 96.4% complete ultra-conserved core eukaryotic genes (CEGs), 93.5% Benchmarking Universal Single-Copy Orthologs (BUSCO) assessment [C, 93.5% (S, 58%, D, 35.5%); F, 1%; M, 5.5%, n, 1440], 98.9% expressed sequence tags (ESTs), and 80.25% average mapped ratio of RNA sequences (Supplementary Data Tables S7–S10).

**Figure 1:**
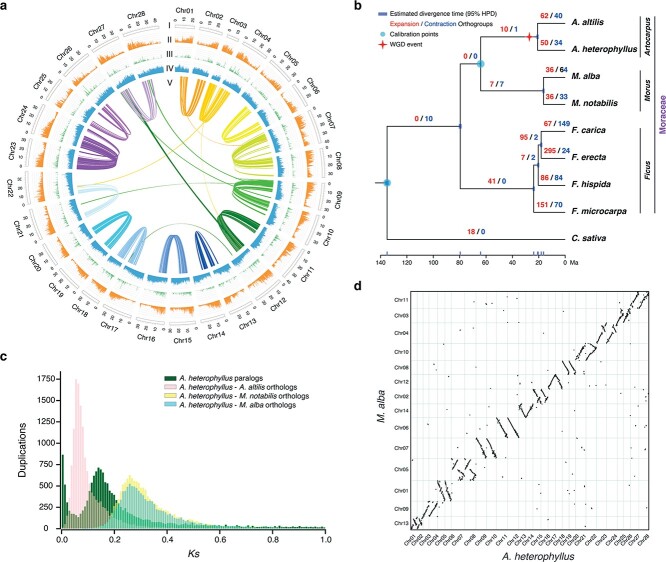
Evolution of the genome of jackfruit and comparative genomic analysis. **a** Genomic characterization of jackfruit. (I) The 28 chromosomes. (II–IV) Distributions of gene density, density of tandem duplications, and percentage of TEs, respectively, in each 200-kb non-overlapping window. (V) Synteny relationships of gene blocks between jackfruit chromosomes. **b** Phylogenetic tree, divergence time, WGD event and orthogroup expansions/contractions among nine angiosperms. The ML tree was constructed with 844 single-copy orthogroups. Divergence times were calculated based on two calibration points (turquoise circles) and indicated by confidence intervals of 95% highest posterior density (HPD) with light purple bars. The numbers in red and blue suggest the expanded and contracted orthogroups along each branch, respectively. An inferred WGD event is marked by star. **c***K*_s_ distributions for paralogs of *A. heterophyllus* and orthologs between *A. heterophyllus* and other species of Moraceae. The peaks of *K*_s_ distributions within *Artocarpus* (green and pink) were lower than that between *Artocarpus* and *Morus* (yellow and cyan), suggesting that the WGD event in *Artocarpus* may have occurred after divergence from *Morus*. **d** Macrosyntenic comparison of whole genomes between *A. heterophyllus* and *M. alba*.

We identified 54.02% (532.44 Mb) of the assembled jackfruit genome as comprising repetitive regions (Supplementary Data Tables S6 and S11), including 52.77% (520.08 Mb) transposable elements (TEs), which displayed a negative correlation with the gene density (Fig. 1a). Long terminal repeats (LTRs) were the most common type of TE, spanning 490.40 Mb and representing ~49.76% of the genome (Supplementary Data Table S12). We inferred that the jackfruit genome encompassed 41 997 protein-coding genes, 99.2% of which were structurally and functionally annotated (Supplementary Data Tables S13 and S14). Additionally, we identified 611 microRNAs (miRNAs), 716 transfer RNAs (tRNAs), 1576 ribosomal RNAs (rRNAs), and 1014 small nuclear RNAs (snRNAs) (Supplementary Data Table S15).

### Genome evolution

To explore the evolutionary relationships between *A. heterophyllus* and eight other species of eudicots, we constructed a phylogenetic tree with 844 single-copy orthologs identified by OrthoFinder. The maximum likelihood (ML) tree resolved *Artocarpus* (*A. heterophyllus* and *A. altilis*) as sister to *Morus* (*M. notabilis* and *M. alba*), which is consistent with a previously reconstructed topology of Moraceae [11, 19]. We used BEAST to estimate the divergence time and predicted that *A. altilis* diverged from *A. heterophyllus* ~20.9 million years ago (Ma) (Fig. 1b). We found 50 orthogroups (584 genes) of the jackfruit genome significantly expanded and 34 orthogroups (20 genes) significantly contracted (*P* ≤ .01) (Fig. 1b). The expanded gene families of jackfruit were significantly enriched for genes associated with response to biotic stimulus, transferase activity, and oxidoreductase activity mainly (Supplementary Data Fig. S3).

Whole-genome duplication (WGD) has occurred commonly in angiosperms and has long been recognized as an important evolutionary force that has shaped plant evolution [20]. To estimate WGD events, we selected *A. altilis* to represent the relatives of *A. heterophyllus*. Compared with two other species of Moraceae, we found that the genomes in *Artocarpus* underwent a relatively recent lineage-specific WGD event (Fig. 1c). Furthermore, we validated the WGD event by relative one-to-one ortholog *K*_s_ of different species in *Artocarpus* with the wgd package (Supplementary Data Fig. S4). The peak showed greater *K*_s_ between *Artocarpus* and *Morus* than within *Artocarpus*, indicating that the WGD event in *Artocarpus* might have occurred after the divergence from *Morus*. The collinearity result between*A. heterophyllus* and *M. alba* further suggested a WGD event in jackfruit (Fig. 1d; Supplementary Data Fig. S5).

### Genomic diversity and population structure in *A. heterophyllus*

We sequenced 295 *A. heterophyllus*, 12 *A. integer*, and other 22 individuals of the subgenera *Artocarpus* and *Pseudojaca*. The samples of *A. heterophyllus* included 262 from China, 19 from Bangladesh, 6 from Thailand, 4 from Malaysia, and 4 from Indonesia (Supplementary Data Table S16). Most of them are landraces via seed propagation and have not been subjected to artificial breeding. The 12 samples of *A. integer* include 9 from China, 2 from Malaysia, and 1 from Brunei (Supplementary Data Table S16). In total, we generated ~4.8 Tb of whole-genome resequencing data. The mapping rates of *A. heterophyllus* and *A. integer* accessions to the reference genome were 95.25 ± 0.03 and 89.01 ± 1.26% respectively, with the average mapping depth ranging from 24.07× to 54.45× (Supplementary Data Table S16). We identified 95 835 447 SNPs via applying the recommended filtering criteria with GATK.

**Figure 2:**
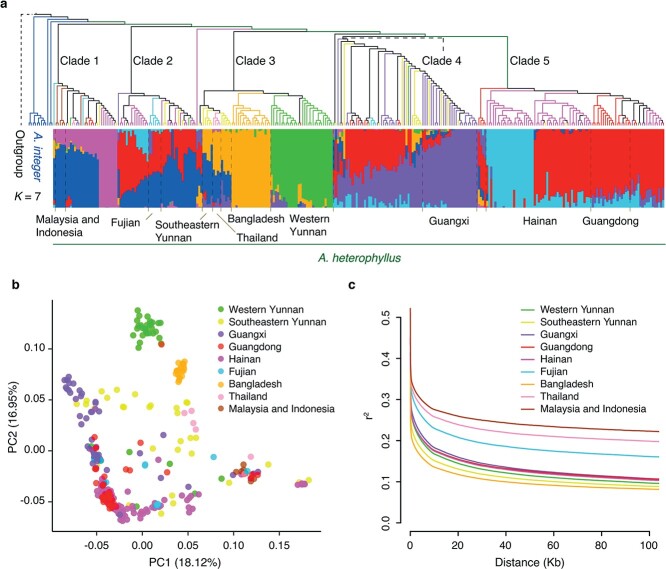
Population structure and genetic diversity of 295 accessions of jackfruit. **a** Phylogeny and population structure of jackfruit individuals. The phylogenetic tree was constructed by merging 19 830 parallel trees of every 50-kb non-overlapping window using ASTRAL. The outgroup comprises 22 individuals of subgenera *Artocarpus* and *Pseudojaca*. Different colors of the branches represent individuals of jackfruit from different groups corresponding to geographic distributions. The population structure of the accessions was estimated using ADMIXTURE with optimal *K* = 7 ancestral populations represented by different colored bars proportional to their genomic contributions. **b** PCA plots of the first two principal components using the core 337 857 SNPs of 295 jackfruit individuals. **c** Decay of linkage disequilibrium of jackfruit groups.

To understand the phylogenetic relationships among accessions of *A. heterophyllus* and *A. integer*, we constructed a phylogenetic tree based on the basic set of 34 378 013 high-quality SNPs. We found that *A. heterophyllus* is clearly separated from *A. integer* except for three accessions of *A. integer* (Fig. 2a). These three individuals of *A. integer* clustered with the clade of *A. heterophyllus*, probably resulting from the introgression with *A. heterophyllus* (see below). The landraces of *A. heterophyllus* mainly split into five clades with high bootstrap support (Fig. 2a; Supplementary Data Fig. S6). The first-diverging clade (Clade 1; Fig. 2a) of *A. heterophyllus* comprised accessions from Malaysia, Indonesia, Thailand, and southern China (Fujian, Yunnan, Guangxi, Guangdong, Hainan). The adjacent clade (Clade 2; Fig. 2a) includes a set of admixed individuals from Fujian, Yunnan, Guangxi, and Hainan in China. The other three groups clustered basically according to geographical distribution. The 19 accessions from Bangladesh formed a monophyletic group, and clustered with most accessions from Yunnan, which were polyphyletic (Clade 3; Fig. 2a). The individuals from Guangxi principally clustered into Clade 4 (Fig. 2a), which also contained some individuals from Yunnan, Guangdong, and Fujian. Clade 5 contained most accessions from Guangdong and Hainan.

To investigate population structure, we first performed principal component analysis (PCA) of the 295 individuals of *A. heterophyllus* based on 337 857 SNPs. The first two principal components (PCs) explained 18.12 and 16.95% of the total genetic variance, respectively (Fig. 2b). PC1 and PC2 separated jackfruit accessions of western Yunnan, Bangladesh, and Southeast Asia, but could not split the accessions of China clearly. We then performed model-based clustering analysis using ADMIXTURE by increasing *K* (number of populations) from 2 to 7 (Supplementary Data Fig. S7). For *K* = 5, the accessions were roughly separated into five groups corresponding to the five identified phylogenetic lineages (Supplementary Data Fig. S7). Specifically, we found a unique grouping containing all accessions from Bangladesh and most accessions from western Yunnan, while most accessions from Guangdong and Hainan formed a distinct cluster (Supplementary Data Fig. S7). For *K* = 6, we found a division between the accessions of Bangladesh and western Yunnan, and a subgroup of individuals from Hainan were further separated from Guangdong at *K* = 7 (Supplementary Data Fig. S7). These results were consistent with the phylogenetic tree and PCA analysis and revealed that jackfruit from western Yunnan and Bangladesh had a close relationship but relatively independent genetic structure. Moreover, the jackfruit accessions from other regions of China had complex population structure, and exhibited disordered branches, a scattered distribution and multiple ancestral components in the results of phylogenetic tree, PCA and population structure, respectively.

We estimated the nucleotide diversity (π), linkage disequilibrium (LD; *r*^2^), and genetic differentiation (*F*_ST_) among the geographically defined populations. The highest genetic diversity was detected for Bangladesh (π = 0.005112) and the western Yunnan population (π = 0.005088; Supplementary Data Table S17). These results were in line with LD analysis showing that Bangladesh had the smallest LD value and fastest decay rate, followed by southeastern and western Yunnan (Fig. 2c). Hainan and Guangdong populations had similar trends in genetic diversity and LD decay, indicating a close genetic relationship between these two groups.

Genome-wide differentiation between western Yunnan and Bangladesh (*F*_ST_ = 0.088) is smaller than that between western Yunnan and Southeast Asia (Thailand, Malaysia and Indonesia) (*F*_ST_ = 0.114–0.181), while all other regions except Guangxi from China have lower pairwise *F*_ST_ values compared with Thailand (*F*_ST_ = 0.052–0.121) than with Bangladesh (*F*_ST_ = 0.093–0.146) (Supplementary Data Table S18). These results are consistent with the phylogenetic and population structure analyses in showing that jackfruit from western Yunnan is more closely related to Bangladesh than Southeast Asia; in contrast, jackfruit from other regions of China is more genetically related with Southeast Asia.

**Figure 3:**
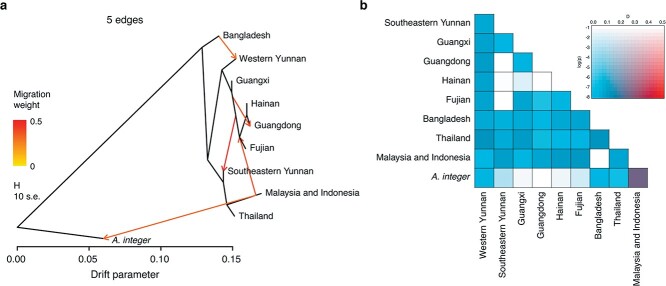
Introgression and genetic relatedness of jackfruit. **a** TreeMix analysis of jackfruit among different geographic groups, with *A. integer* as the outgroup. **b** Four-taxon ABBA-BABA test showing extensive introgression among the jackfruits of different geographic regions.

TreeMix analyses indicated signals of introgression involving at least five migration events between populations, i.e. from Bangladesh to western Yunnan, from the jackfruit ancestor of Fujian, Hainan, and Guangdong to that of Southeast Yunnan, from jackfruit of Malaysia and Indonesia to the jackfruit ancestor of Fujian, Hainan, and Guangdong, from Guangxi to Guangdong, and from the jackfruit ancestor of Malaysia and Indonesia to *A. integer* (Fig. 3a). The widespread gene flow among jackfruits of different geographic regions was further supported by ABBA-BABA tests (Fig. 3b). In addition, the ABBA-BABA tests revealed significant gene flow between *A. integer* and jackfruit from Malaysia and Indonesia (Fig. 3b). Furthermore, we found jackfruit between western Yunnan and Bangladesh shared more identity by descent (IBD) haplotypes than other comparisons with Bangladesh (Supplementary Data Fig. S8). On the contrary, some of the jackfruit from other regions of China (except western Yunnan) shared extensive IBD haplotypes with jackfruit of Southeast Asia. Moreover, there were few IBD haplotypes shared by jackfruit among Bangladesh, Malaysia, and Indonesia. Collectively, the results of phylogenetic, population structure, and gene flow analyses suggest that jackfruit might have been introduced to China via at least two independent routes, i.e. South Asia and Southeast Asia.

### Signals of selective sweep between jackfruit and cempedak

**Figure 4:**
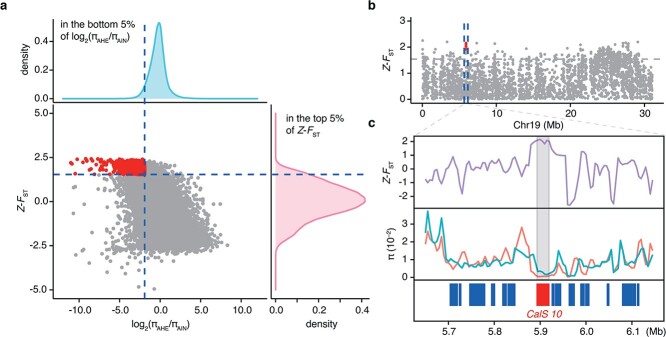
Genomic signatures of selection signals between jackfruit and cempedak. **a** Distribution of population differentiation (*Z*-transformed *F*_ST_) and nucleotide diversity ratios [log_2_ (π_AHE_/π_AIN_)] between jackfruit and cempedak by 10-kb sliding windows and 5-kb steps. Genomic regions of high population differentiation between jackfruit and cempedak and low nucleotide diversity within jackfruit are marked by red dots in the upper left (above 95% threshold). **b** Genomic landscape of CDRs on chromosome 19 between jackfruit and cempedak. Vertical blue dashed lines represent the 500-kb region around the *CalS 10* gene. Red dots indicate signals of selective sweep in the region. **c***Z*-transformed *F*_ST_ and π values of the *CalS* gene linking region between jackfruit and cempedak. Red line indicates the nucleotide diversity of *A. heterophyllus* (AHE group). Green line indicates the nucleotide diversity of *A. integer* (AIN group).

As a sister species to jackfruit (*A. heterophyllus*), cempedak (*A. integer*) has a similar, but much smaller fruit (0.6–3.5 kg) [21]. It is an important tropical fruit in Malaysia and prevalently cultivated in southern Thailand and parts of Indonesia [22]. To identify genetic selection signals between jackfruit and cempedak, we detected the candidate divergent regions (CDRs) based on high genetic differentiation (in the top 5% of *Z*-transformed *F*_ST_) and low nucleotide diversity [in the bottom 5% of log_2_ (π_AHE_/π_AIN_)] throughout the whole genome with 10-kb sliding windows. In total, we identified 1074 genes of 4290 CDRs as being under selection (Fig. 4a; Supplementary Data Table S19). These genes were mainly enriched in callose synthase process (K11000, adjusted *P* value = .003) (Supplementary Data Table S20), containing six callose synthase genes (*CalS*) and two unannotated genes (Fig. 4b and c; Supplementary Data Fig. S9). CalS is the key enzyme in callose synthesis and is also associated with pollen development, cell plate formation, plasmodesmata regulation, and response to stress [23, 24]. The neighbor-joining tree showed that CalS proteins between *A. heterophyllus* and *Arabidopsis thaliana* displayed a high degree of homology (Supplementary Data Fig. S10). Moreover, we also found that most members of the callose synthase complex were under selection, including sucrose synthase, UDP-glucose transferase, and Rho-like protein (Supplementary Data Table S19). The jackfruit plant produces a multiple fruit formed by the fusion of multiple flowers in an inflorescence, and the edible part of jackfruit comes from the perianth [2]. Similar to jackfruit, cempedak is a multiple fruit derived from a whole inflorescence, but obviously smaller than jackfruit, and has fewer edible bulbs. The selective signals between jackfruit and cempedak uncovered the key regulators in the callose synthesis pathway, which impact pollination and fertility. Accordingly, we presumed that the different fruit size between jackfruit and cempedak is likely associated with pollination.

Interestingly, we also found three transporter protein genes associated with fruit ripening, including two members of the sugar transporter family, SWEET (*AHE.Chr18.535*, *AHE.Chr18.642*), and one auxin transport protein, BIG (*AHE.Chr22.1004*), within the regions under selection (Supplementary Data Table S19). SWEET and BIG are required for sugar efflux and auxin polar transport, respectively [25, 26]. We also identified two U-box proteins, six F-box proteins, and 18 E3 ubiquitin-protein ligases in connection with the ubiquitination pathway in the CDR region (Supplementary Data Table S19). The ubiquitination pathway regulates cytological and physiological processes via selectively removing regulatory proteins efficiently and rapidly, thus modulating responses to environmental stress as well as plant development [27]. Altogether, we identified signals of divergent selection for several important candidate genes associated fruit development and ripening as well as responses to environment stress.

### Signals of selective sweep between firm variety and soft variety of jackfruit

**Figure 5:**
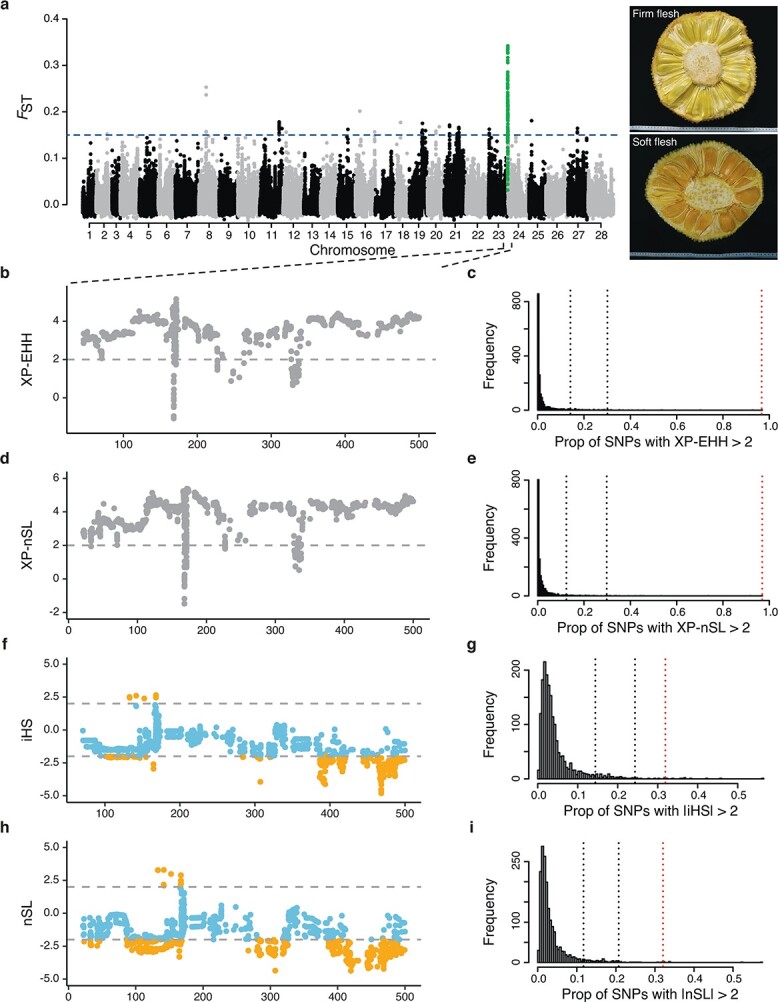
Schematics of positive selection signatures between firm-flesh and soft-flesh populations of jackfruit. **a** Manhattan plot of *F*_ST_ between firm and soft types of jackfruit. Horizontal blue line represents the cut-off of high population differentiation (*F*_ST_ = 0.15). The 500-kb region of chromosome 24 was identified as a high differentiation region and is marked in green. **b**, **d** Manhattan plots of XP-EHH and XP-nSL statistics for comparing firm-flesh and soft-flesh type fruits. The horizontal dashed line represents the threshold of positive selection signal (XP-EHH > 2, XP-nSL > 2). **c**, **e** The highest concentrations of significant XP-EHH and XP-nSL signals were detected in the 500-kb region of chromosome 24 (marked with red dashed line) with comparisons of the genome-wide distribution based on non-overlapping windows of 500 kb. The black dashed lines indicate the 95 and 99% percentiles. **f**, **h** Patterns of normalized iHS and nSL scores of the candidate region in the firm-flesh population. The horizontal dashed lines indicate the threshold of positive selection signal (|iHS| > 2, |nSL| > 2) (marked in orange). **g**, **i** High concentrations of significant |iHS| and |nSL| signals were detected in the 500-kb region of chromosome 24 (marked with red dashed line), with comparisons of the genome-wide distribution based on non-overlapping windows of 500 kb. The black dashed lines indicate the 95 and 99% percentiles.

The varieties of jackfruit are mainly classified into those having firm or soft fruits. The perianth of the firm-flesh type is crisp and remains firm even at full ripeness, while the perianth of the soft-flesh type becomes pulpy, soft, and spongy during ripening [2, 28]. To investigate the genetic basis of fruit texture of jackfruit, we compared the genomes of firm and soft types for strong selective signatures. Based on this, we detected high population differentiation in the 500-kb region of chromosome 24 (average weighted *F*_ST_ = 0.186; Fig. 5a). We further calculated the cross-population extended haplotype homozygosity (XP-EHH) and two-population haplotype-based statistic (XP-nSL) to examine the selective sweeps around the 500-kb region. We identified strong positive selection signals in this region from the firm type of jackfruit (XP-EHH > 2, XP-nSL > 2), and the region contained the highest concentration of significant hits according to the XP-EHH and XP-nSL tests across the genome (96.7 and 96.9% of all tested SNPs, respectively, Fig. 5b–e). Moreover, we calculated the normalized integrated haplotype score (iHS) and the number of segregating sites by length (nSL) to explore the recent positive selection of this region in the firm-type population. In a genome-wide comparison, we detected significant positive selection signals for 31.9% of all tested SNPs in the region exhibiting |iHS| > 2.0 (Fig. 5f and g). We also observed similar results via an nSL test (32.1% of all tested SNPs; Fig. 5h and i), which is sensitive to incomplete selective sweeps [29]. These results indicated that the 500-kb region on chromosome 24 was the strongest candidate for positive selection in the firm type of jackfruit.

**Figure 6:**
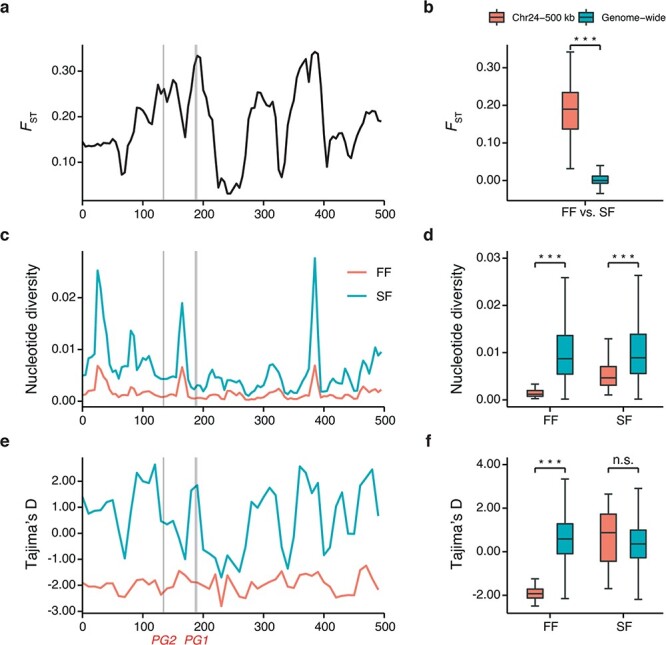
Signals of selective sweep in firm-flesh and soft-flesh populations of jackfruit. Left panels: Schematics of selective sweeps for the ~500-kb region surrounding *AhePG* genes. The *AhePG* genes are visualized with shadows. Right panels: Comparison of these statistics between the *AhePG* region (red boxplot) and genomic background (green boxplot). Statistics were calculated separately for individuals from firm-flesh populations (FF) and soft-flesh populations (SF) of jackfruit. Statistical significance between candidate regions and the rest of genome-wide regions was calculated using the Mann–Whitney *U*-test (n.s., not significant, ^***^P <1e−4). **a**, **b** Genetic differentiation (*F*_ST_). **c**, **d** Nucleotide diversity. **e**, **f** Tajima’s *D*.

It is believed that strong positive selection can result in selective sweeps for accumulating beneficial mutations [30]. In order to further elucidate the evolutionary history between firm and soft types of jackfruit around the selective region, we performed annotation and functional enrichment analysis of the genes in the region. We identified a total of 48 genes (Supplementary Data Table S21), which were enriched for the KEGG pathways of pathogen-inducible salicylic acid glucosyltransferase (K13691; adjusted *P* value = .000018), phosphatidylinositol-bisphosphatase (K01099; adjusted *P* value = .0027), and polygalacturonase (K01184; adjusted *P* value = .0027) (Supplementary Data Table S22). Specifically, we found two genes encoding polygalacturonase (PG), which were known to contribute to fruit texture in strawberry and peach [31]. We then tested their patterns of genetic variation in the firm and soft types. We found that the genetic differentiation was remarkable at the *AhePG* locus compared with the genome-wide background (Fig. 6a and b). Additionally, the level of nucleotide diversity at the *AhePG* locus was exceptionally low compared with the whole genome in these two types (Fig. 6c and d). We observed a much stronger decline of nucleotide diversity in the firm type compared with the soft type. Furthermore, we only found significantly low Tajima’s *D* statistics in the firm type (Fig. 6e and f). These results support the occurrence of strong positive selection at *AhePG* locus in the firm population. Haplotype differentiation of *AhePG1* (*AHE.Chr24.26*) and *AhePG2* (*AHE.Chr24.22*) loci separated most of the firm-type accessions from the soft-type accessions (Supplementary Data Fig. S11), indicating their potential contributions to fruit texture of jackfruit.

Furthermore, we identified 190 genes encoding key enzymes related to pectin degradation in the jackfruit genome (Supplementary Data Table S23), such as polygalacturonases, pectate lyases, pectinesterases, β-galactosidases, α-galactosidases, and α-arabinofuranosidases. The analyses of gene expression profiles in jackfruit pulps of different firmness revealed that most pectin degradation-related genes had low expression levels or were even not expressed, or were expressed at the initial stage of fruit softening (Supplementary Data Fig. S12), whereas the expression level of the *AhePG1* gene increased sharply along with the decrease in jackfruit firmness and the enhancement of fruit softening. The gene expression of *AhePG1* was significantly negatively correlated with fruit firmness (*r* = −.836, *P* = .0096; Supplementary Data Fig. S13). These results suggest that the *AhePG1* gene is probably a major factor in determining pectin degradation in jackfruit.

## Discussion

We used the PacBio and Illumina platforms to assemble the chromosome-level genome of *A. heterophyllus* (Supplementary Data Table S6), which spanned 985.63 Mb and comprised 41 997 protein-coding genes. This is the first report of an assembled jackfruit genome anchored to chromosomes based on a high-density linkage map. The high-quality chromosome-level genome of jackfruit is foundational and valuable for insights into the genome evolution and genetic architecture of agronomic traits.

We have reconstructed a putative domestication history of jackfruit, with a focus on the origin and introduction routes of the species to China. We found that jackfruit of China could be traced back to independent introductions from Southeast Asia (Malaysia, Indonesia, and Thailand) and South Asia (Bangladesh), both of which might trace back to the Silk Road. The Silk Road was a grouping of major historical trade arteries and cultural transmission routes in Asia, and primarily consisted of two major routes, the Overland Silk Road and the Maritime Silk Road [32]. These routes promoted economic and cultural development, as well as the dispersal of crops. For example, grapes, pomegranates, peppers, corn, cucumbers, and dozens of other crops spread to China along the Silk Road [33]. The Overland Silk Road emerged in the Chinese Han Dynasty in the second century before the common era (BCE) and continued to be important until the 18th century of the common era (CE) [34]. Notably, the east line of the Silk Road in South Asia connected western Yunnan with ancient India at their closest distances that were not threatened by the Tibetan army and nomadic nations of the northern steppes. Thus, it attracted the attention of monks who sought to go west towards India [35]. In addition, the rise and spread of Buddhism along the Silk Road played an important role in crop dispersion. For example, mango originated from India and was likely introduced to Southeast Asia outside its original range of cultivation by Buddhist monks in the fourth and fifth centuries [36]. Similarly, jackfruit of ancient India (including Bangladesh) was probably dispersed to China via the Buddhist monks as they traveled. However, most of the Overland Silk Road passed through vast virgin forests, treacherous mountains and valleys, and deserts, not to mention that it was vulnerable to war and political conflicts in the Western Regions and blocked at certain times. Therefore, the Maritime Silk Road rose to prominence in the seventh century and became a vital route for trade and the spread of culture and religion around the Indian Ocean through the 16th century. The Maritime Silk Road brought China closer to Southeast and South Asia, possibly promoting the spread of jackfruit. According to historical documentation [37], jackfruit was widely popular and planted in Hainan, Guangdong, Guangxi, the southeast of Yunnan, and Fujian of China during the 14th to 20th centuries.

As a multiple fruit with fusion of multiple flowers in an inflorescence, the fruit size of jackfruit is correlated with how many flowers on an inflorescence get pollinated. Our genomic scans for selection signatures identified callose synthase genes that may play important roles in fruit size evolution between jackfruit and cempedak. CalS is the key enzyme in callose synthesis and plays a role in pollen development, cell plate formation, plasmodesmata regulation, and response to stress [23, 24]. In *Arabidopsis*, five out of the 12 CalSs, including CalS5, CalS9, CalS10, CalS11, and CalS12, play important roles during sporophytic development and pollination [38]. Notably, AHE.Chr18.898 (AheCalS5) was homologous with CalS5 of *A. thaliana* (Supplementary Data Fig. S10), which is essential for exine formation and pollen viability. A previous study revealed that knockout mutations of *CalS5* led to severe reduction in fertility in *Arabidopsis* [39]. Mijin and Ding [40] demonstrated that each edible bulb of flesh within a jackfruit develops from an entire carpel after fertilization, while the unfertilized carpels form perigone, a non-palatable part of the syncarp. Furthermore, a recent study found that assisted pollination can produce syncarp with heavier weight and affect the fruit size of jackfruit [41]. Therefore, pollination and fertilization processes, including positive selection on genes associated with the callose synthesis pathway, is probably an important mechanism underlying fruit size between jackfruit and cempedak.

The firm type of jackfruit is more popular in the fresh food market, and has a longer shelf life than the soft type. We found that these two types of jackfruit do not form clades in phylogenetic analyses (Supplementary Data Fig. S6), and this is consistent with prior studies [42]. This suggests that changes in fruit texture in jackfruit probably result from very few genetic loci instead of genome-wide genetic variation. Typically, textural change of ripening fruit depends on the cell wall composition and cellular adhesion, particularly the loss of pectins. We identified strong positive selection at the *AhePG* locus in firm-type jackfruit. PG is a crucial enzyme in pectin degradation and contributes to fruit softening. Recent studies on strawberry, apple, and papaya have revealed that PG-mediated pectin disassembly plays a central role in fruit softening [43, 44]. Moreover, endo-PG has been shown to function as an essential factor to achieve a melting flesh fruit texture in peach [45, 46]. Specifically, the expression of *endo-PG* was higher and increased along with softening in melting flesh peaches, while it was expressed at lower and steadier levels in non-melting flesh peaches [45]. Recently, it was reported that two *endo-PG* genes controlled the peach melting flesh and stone adhesion traits [47]. Similarly, PG activity in ripe fruits of the soft type was higher than in the firm type, and this indicates that PG may be related to the difference in fruit texture between these two types of jackfruit [28, 48]. In the present study, we observed that two *PG* genes were located in a 500-kb region under strong selection by comparing firm and soft types of jackfruit. Furthermore, the gene expression of *AhePG1* was significantly positively correlated with fruit softening. Therefore, we suggest that the *AhePG1* gene may have a pronounced effect on the texture of jackfruit by controlling pectin degradation. However, the specific functions of these genes under selection in jackfruit still need further verification.

## Materials and methods

### Genome sequencing, assembly, and annotation

The jackfruit cultivar ‘S10’, a superior clonal line from seed selection with large fruit and high sweetness, was used for genome sequencing. We collected fresh leaves of ‘S10’ in the germplasm repository of Guangdong Ocean University (Zhanjiang, China) for genome sequencing. We prepared genomic DNA of fresh leaves and constructed paired-end libraries of 350-bp inserted size, PacBio 20-kb insertion libraries and 10× Genomics libraries.

We used FALCON v0.3.0 [49] to correct errors in PacBio long reads by removing short reads of <5 kb. We assembled contigs and applied the Quiver algorithm [50] and PILON v1.22 [51] to correct the assembly reads. We used fragScaff software [52] to perform 10× Genomics scaffold extension. We generated superScaffold from the alignment between linked reads of the 10× Genomics library and consensus sequence of PacBio assembly using BOWTIE v2.2 [53] for assembling the draft genome. We evaluated jackfruit genome size using GCE [54]. We estimated the quality of the assembled genome with CEGMA [55], BUSCO [56], and RNA-seq data.

Further, we anchored scaffolds to chromosomes based on the genetic linkage map. Subsequently, we identified the repetitive sequences and annotated gene functions using several databases. Detailed methods are included in Supplementary Data.

### Phylogenetic analysis and divergence time estimation

We used OrthoFinder v2.2.7 [57] to classify the orthogroups of proteins from *A. heterophyllus* and eight other species of angiosperms, including breadfruit (*A. altilis*), mulberry (*Morus alba*, *Morus notabilis*), fig (*Ficus erecta*, *Ficus carica*), banyan (*Ficus hispida*, *Ficus microcarpa*), and marijuana (*Cannabis sativa*). We aligned the proteins for each gene within single-copy orthogroups in MUSCLE v3.8.31 [58]. For each gene alignment, we reconstructed an ML tree in IQ-TREE v1.6.12 [59] with *C. sativa* as the outgroup. We merged the gene trees to construct the phylogenetic tree using ASTRAL v5.14.2 [60]. For divergence time estimation, we used BEAST v2.6.2 [61] with one fossil constraint. We performed a uniform prior distribution for the fossil calibration, setting 64 Ma as the minimum bound of stem *Artocarpus*. We set the constraint for stem of Moraceae as a secondary calibration based on the age estimate (81.7–93.3 Ma) [19]. Within BEAST, we used the Markov chain Monte Carlo method for 10 000 000 generations with sampling every 1000 generations. We used CAFÉ v4.2 [62] to calculate the gene family expansions and contractions with *P*-value <.01. We then analyzed GO enrichment and KEGG enrichment for significantly expanded and contracted genes using the R package clusterProfiler [63] against all *A. heterophyllus* genes as background*.*

Detailed methods are included in Supplementary Data.

### Collinearity and whole-genome duplication

For the collinearity analysis, we used the python version of MCScan (version 20101014) [64] to identify syntenic blocks between *A. heterophyllus* and *M. alba*. We visualized the syntenic blocks of at least 30 shared genes within the *A. heterophyllus* genome in CIRCOS v0.69 [65]. For identifying the WGD events, we applied the wgd package [66] to construct the *K*_s_ distribution of paralogs or one-to-one orthologs from five genomes of Moraceae (*A. heterophyllus*, *A. altilis*, *M. notabilis*, *M. alba*, *Ficus erecta*) and four transcriptomes (*Artocarpus nitidus*, *Artocarpus hypargyreus*, *Ficus hirta*, *Broussonetia papyrifera*). The sources of these genomes were the same as for the phylogenetic analysis. The transcriptional data of *A. nitidus* and *A. hypargyreus* were sequenced, while the RNA-seq data of *F. hirta* (SRR1909647, SRR1909654) and *B. papyrifera* (SRR1477753) were downloaded from NCBI. These RNA sequences were assembled and predicted using Trinity.

### Whole-genome resequencing and variation calling

We resequenced 295 individuals of *A. heterophyllus* (including 37 firm-flesh and 26 soft-flesh individuals) and 34 individuals of its relatives (including 12 individuals of *A. integer*; Supplementary Data Table S16). We filtered paired-end resequencing data by removing sequences containing adapter or poly-N and low-quality reads (reads with >30% bases having Phred quality ≤25). Further, we obtained clean data using the in-house pipeline QC_pe (https://github.com/scbgfengchao/) [67]. We mapped the clean reads to the genome of *A. heterophyllus* using BWA v0.7.17 [68]. Then we converted the alignments into the BAM format and filtered the unmapped and non-unique reads using SAMtools v1.9 [69]. We marked PCR duplicates and realigned the sequences around indels using GATK v4.1.3.0 [70]. We calculated coverage and sequencing depth based on the final BAM files using SAMtools v1.9. We obtained genome Variant Call Formats (gVCFs) for each individual using HaplotypeCaller of GATK software, with filtering of sites with base quality <30. Then we merged all gVCF files to generate the VCF file of all individuals using GATK CombineGVCFs and GenotypeGVCFs. Furthermore, we used GATK VariantFiltration to remove variant sites with filter expression QD < 2.0 || FS > 60.0 || MQ < 40.0 || MQRankSum < −12.5 || ReadPosRankSum < −8.0. We also filtered the sites using parameters of --min-meanDP 5 --max-meanDP 200 --max-alleles 2 --max-missing 0.5 with VCFtools v0.1.13 [71]. As a result, we obtained a total of 95 835 447 SNPs. We used VCFtools to construct the basic set of SNPs by excluding SNPs with non-biallelic, max-missing rate >0.05. Furthermore, we removed SNPs with minor allele frequency (MAF) < .05 and *r*^2^ > .2 to generate the core set for PCA and population structure analysis.

### Phylogenetic and population genetic analysis

We extracted the SNPs present in all resequenced individuals to construct an ML tree using IQ-TREE for each 50-kb non-overlapping window, with 22 individuals of the subgenera *Artocarpus* and *Pseudojaca* as the outgroup. Then we merged the trees to construct the species tree using ASTRAL v5.14.2. We performed PCA and population structure analysis with the core set of 295 *A. heterophyllus* individuals using PLINK v1.90b6.10 [72] and ADMIXTURE v1.3.0 [73], respectively. We calculated nucleotide diversity (π) and genetic differentiation (*F*_ST_) in 10-kb windows using VCFtools, and analyzed LD using PopLDdecay v3.40 [74].

### Estimation of gene flow

According to the results of PCA and STRUCTURE analyses, we split the jackfruit of Yunnan into southeastern Yunnan and western Yunnan groups and combined the jackfruit of Malaysia and Indonesia into one group. To detect the gene flow across the geographical populations of *A. heterophyllus*, we performed TreeMix v1.12 [75] using biallelic SNPs that were unlinked and non-missing data, with blocks of 100 SNPs and -se option to calculate standard errors of migration weights. In the TreeMix analysis, we set *A. integer* as the outgroup, excluding the three admixed individuals of *A. integer* (XM-14#, ZJ-13, ZJ-17). Considering admixture events (‘migration’) to improve the fit of the inferred tree, TreeMix builds ML graphs that link populations with their common ancestor via the covariance matrix of allele frequencies between populations. Furthermore, we applied ABBA-BABA tests in Dsuite [76] using the biallelic SNPs of all *A. heterophyllus* and nine *A. integer* (excluding XM-14#, ZJ-13, ZJ-17), containing no missing data. We set the other 11 individuals of subgenus *Artocarpus* and 11 individuals of subgenus *Pseudojaca* as the outgroup. The ABBA-BABA test uses the *D* statistic as a measure of discordant genealogies on four-taxon, rooted trees. The form of four-taxon topology is (((P1, P2), P3), outgroup), and P1 to P3 are ingroups. Gene flow between P1 and P3 leads to a *D*-score <0, while gene flow between P2 and P3 exhibits a *D*-score >0. For evaluating whether the *D*-score is significantly different from zero, we used the Dtrios module in Dsuite to calculate the *Z*-score (*Z* = *D*/standard error of *D*) [76], and outputted the *P*-values. For further insight into relationships of jackfruit in different regions, we applied Beagle v4.1 [77] to analyze the IBD blocks, setting the following parameters: window = 100 000, overlap = 10 000, ibdtrim = 100, ibdlod = 3.

### Selective sweep analysis

To detect regions and genes under selection, we removed the SNPs with max-missing rate >0.2. Then, we calculated *F*_ST_, π, and Tajima’s *D* of different populations using VCFtools with a 10-kb sliding window and a step size of 5 kb. For identifying potential selective sweeps between the *A. heterophyllus* (AHE) and *A. integer* (AIN) groups, we selected the windows simultaneously with high genetic differentiation {the top 5% of *Z*-transformed *F*_ST_ and low nucleotide diversity [the bottom 5% of log_2_ (π_AHE_/π_AIN_)]} as CDRs in populations of *A. heterophyllus*; *Z*-transformed *F*_ST_ = (per window *F*_ST_ − mean *F*_ST_)/standard deviation of *F*_ST_ [78]. We obtained the candidate genes in CDRs and analyzed KEGG enrichment using the R package clusterProfiler*.*

We found high population differentiation in the 500-kb region of chromosome 24 between the firm type (FF group) and soft type (SF group) of jackfruit. To test for possible positive selection, we measured the cross-population extended haplotype homozygosity (XP-EHH) [79], two-population haplotype-based statistic (XP-nSL) [80], integrated haplotype score (iHS) [81], and the number of segregating sites by length (nSL) using Selscan v1.3.0 [82] with its default parameters. Then, we used 100 frequency bins to normalize iHS and nSL across the whole genome. We normalized all these haplotype-based values with a 500-kb window. For the XP-EHH and XP-nSL analyses, we used sets of haplotypes in the soft-type population as a reference. Positive scores indicate hard or soft sweeps in the firm-type population, and score >2 was considered to represent candidate selective regions. We calculated the proportion of SNPs with XP-EHH > 2, XP-nSL > 2, |iHS| > 2 or |nSL| > 2 in each 500-bp window. We evaluated statistical significance using the ranking of genome-wide windows, and excluded windows having <100 SNPs. We compared statistical significance between candidate regions and the rest of the genome-wide regions using the Mann–Whitney *U*-test. We identified the candidate genes combined with low nucleotide diversity [the bottom 5% of log_2_(π_FFgroup_/π_SFgroup_)] in the 500-kb region of chromosome 24, and performed KEGG analysis with the R package clusterProfiler.

### Transcriptional expression level of candidate genes related to jackfruit texture

To investigate the expression pattern of genes associated with jackfruit texture, we collected jackfruit pulps of different firmness during fruit softening. We measured the hardness value of jackfruit pulps using a hardness tester, taking six biological replicates for each group. Then, we mixed the samples within each group and subjected them to transcriptome analysis. We trimmed the RNA sequences to remove adaptors and contaminating sequences, and preprocessed to remove low-quality sequences with QC script [67]. Then we mapped the sequences to the jackfruit genome with HiSat2 (http://ccb.jhu.edu/software/hisat/index.shtml), and assembled them based on the alignment results with StringTie [83]. Next, we merged the non-redundant assembled set of each sample with StringTie’s merge function. According to the read-count data provided by StringTie, we calculated the fragments per kilobase of transcript per million fragments mapped (FPKM) values for each gene using Ballgown [83]. We performed correlation analysis between *AhePG1* gene expression and fruit firmness using the cor function in R and fitted the result by linear regression using the lm function. We removed the genes of FPKM value <1 in all samples, and drew a gene expression heat map of the remaining genes using the R package ComplexHeatmap (http://www.bioconductor.org/packages/3.8/bioc/html/ComplexHeatmap.html).

## Acknowledgements

This work was supported by the Institution of South China Sea Ecology and Environmental Engineering, Chinese Academy of Sciences (No. ISEE 2018YB02), Projects of Enhancing School with Innovation of Guangdong Ocean University (GDOU2013050217, GDOU2016050256), and Science and Technology Projects of Guangzhou City (202102020341).

## Author contributions

M.K. and Y.L. designed and supervised the project. T.L. collected materials. X.L. performed the experiments and bioinformatics analysis. X.L. designed and visualized the figures. X.L. and M.K. wrote the manuscript. M.K., C.F., and A.J.H. revised the manuscript. All authors read and proved the final version of this manuscript.

## Data availability

The raw genomic Illumina sequences, PacBio sequences, and transcriptome data have been deposited in the NCBI Sequence Read Archive under accession numbers PRJNA788174 and PRJNA791757.

## Conflict of interest

The authors declare no conflict of interests.

## Supplementary data

Supplementary data is available at *Horticulture Research* online.

## Supplementary Material

Web_Material_uhac173
