# Supplementary material for: Jackfruit genome and population genomics provide insights into fruit evolution and domestication history in China
Source: Hortic Res. 2022 Aug 4;9:uhac173. doi: 10.1093/hr/uhac173 (PMC9533223; doi:10.1093/hr/uhac173)
Supplement: Web_Material_uhac173 [file web_material_uhac173.docx]

**Supplemental information**

**Jackfruit genome and population genomics provide insights into fruit evolution and domestication history in China**

Xinggu Lin, Chao Feng, Tao Lin, AJ Harris, Yingzhi Li, Ming Kang

**Contents**

1. Supplementary Notes

2. Supplementary Figures S1-S13

3. Supplementary Table S1-S23

**Title**

Jackfruit genome and population genomics provide insights into fruit evolution and domestication history in China

**Authors**

Xinggu Lin^1,2,#^, Chao Feng^1,#^, Tao Lin^1,2^, AJ Harris^1^, Yingzhi Li^3,*^, Ming Kang^1,4,*^

**Affiliations**

^1^ Key Laboratory of Plant Resources Conservation and Sustainable Utilization, South China Botanical Garden, Chinese Academy of Sciences, Guangzhou, China

^2^ University of Chinese Academy of Sciences, Beijing, China

^3^ Horticulture and Forestry Department, Guangdong Ocean University, Zhanjiang, China

^4^ Center of Conservation Biology, Core Botanical Gardens, Chinese Academy of Sciences, Guangzhou, China

***Correspondence:** Ming Kang (mingkang@scbg.ac.cn), Yingzhi Li (liyz@gdou.edu)

^#^These authors contributed equally to this work.

**Running title:** Jackfruit genome and population genomics.

**Supplementary Notes**

**Plant materials for genome assembly**

The jackfruit cultivar ‘S10’ (firm flesh type) was planted in the germplasm repository of Guangdong Ocean University (Zhanjiang, China). We collected the fresh leaves of ‘S10’ for sequencing and genomic analysis, and used the tissues of flower, leaf, stem and fruit for RNA-sequencing and applied to genome assembly.

**Genome sequencing and assembly**

We prepared genomic DNA of fresh leaves following the modified CTAB protocol [1], and we used it to construct a paired-end library of 350-bp inserted-size for DNA sequencing on the Illumina HiSeq 6000 platform. We also extracted high molecular-weight (HMW) genomic DNA for PacBio 20-kb insertion libraries [2] and 10 × Genomics libraries construction (the manufacturer’s protocol ‘Chromium Genome v1, PN-120229’). We constructed the reference genome by combining the Illumina paired-end reads (total data of 97.88 Gb, ~92.34× coverage), PacBio single-molecule long reads (total data of 111.85Gb, ~105.52× coverage), and 10× Genomics (total data of 116.84Gb, ~110.23× coverage) (Supplementary Table S2).

We used ‘daligner’ in FALCON v0.3.0 [3] to correct error in PacBio long reads by removing short reads less than 5 kb. Then, we identified the consensus sequences using the following parameters: *-- output_multi --min_idt 0.70 --min_cov 4 --max_n_read 200*. Subsequently, we excluded the overlaps among error-corrected reads using parameters *--max_diff 100 --max_cov 100 --min_cov 2* in FALCON, and constructed string graphs with the reads overlaps using Myers’ algorithm [4]. We assembled contigs from the string graph and applied the Quiver algorithm [2] and PILON v1.22 [5] to correct the assembly reads. We used fragScaff software [6] to perform the 10× Genomics scaffolds extending with parameters *-m 3000 -q 30 -E 30000 -o 60000 -C 5 -j 1 -u 3*. Firstly, we generated superScaffold from the alignment between linked-reads of 10× Genomics library and consensus sequence of PacBio assembly using BOWTIE v2.2 [7]. Then we assembled the draft genome only with the consensus sequences supported by the linked-reads.

We performed an analysis of *k*-mer distribution to evaluate the jackfruit genome size using the program in GCE [8]. We estimated the completeness of the assembled genome and conserved genes in jackfruit with analyses in CEGMA [9] and BUSCO [10], respectively. We assembled the expressed sequencing tags (ESTs) using the RNA-Seq data of four different tissues (stems, leaves, inflorescence and fruits) via Trinity v2.8.4 [11]. Thereafter, we aligned the ESTs to the assembled genome by BLAT [12] with identity ≥ 90% and coverage ≥ 50%. In addition, we directly mapped the RNA-Seq data to the genome assembly with TopHat2 [13].

**Genetic map construction**

We constructed a *de novo* molecular genetic map using the RAD-seq data of 108 individuals from the F1 population between firm type vs. soft type jackfruits. We called the SNPs from RAD data using STACKS [14], and selected potential SNP markers according to the expected segregation ratio. For example, the expected segregation ratio of one heterozygous and one homozygous SNP markers between two parents was 1:1, while that of two heterozygous SNP alleles between these two parents was 1:2:1. Then, we filtered out the markers with significantly distorted segregation (*P*-value < 0.01) using a chi-square test. To run linkage analysis for markers present at least 90%, we adopted the double pseudo-test cross strategy [15] and JoinMap 4.0 [16], in which we applied a logarithm of the odds linkage threshold of 5.0 to identify linkage groups. In the end, we divided these SNP markers into 28 linkage groups (Supplementary Table S4, Supplementary Fig. S2). We constructed the linkage groups and anchored scaffolds to chromosomes by aligning and scanning all candidate SNP markers.

**Repeat annotation**

We identified the repetitive sequences using the Repbase-based method [17] and a *de novo* approach at both the DNA- and protein-levels. We acquired the repetitive elements by RepeatMasker and RepeatProteinMask against the Repbase database [18] and MIPS Repeat Element Database [19]. We also searched for repeats in the *de novo* repeat libraries built with the Piler [20], RepeatScout [21] and LTR-FINDER [22] programs.

**Genome annotation**

We predicted the annotation of protein-coding genes by combining *de novo* gene identification with homology-based and RNA-Seq-based prediction. We performed *de novo* prediction of gene models in Augustus [23], GlimmerHMM [24], SNAP [25], Genscan [26], and Geneid [27]. We generated gene models using Genewise v2.2.0 [28] and protein alignments of *Arabidopsis thaliana* [29], *Vitis vinifera* [30], *Fragaria vesca* [31], *Ananas comosus* [32], *Durio zibethinus* [33], *Morus alba* [34] and *Ficus hispida* [35] to *A. heterophyllus* based on tBlastN [36] (E-value < 1E-5). We applied two RNA-Seq-based methods for prediction. On the one hand, we mapped RNA-Seq data to the jackfruit genome in TopHat2 [37] and subsequently generated gene models by Cufflinks [38]. On the other hand, we assembled the ESTs from the RNA-Seq data in Trinity and aligned these ESTs to the assembled genome before generating gene models in PASA [39]. In the end, we merged all the predicted genes to form the non-redundant gene model in EVM [40].

We annotated functions of the predicted genes on the basis of the best match by comparing the protein sequences against the Swiss-Prot [41], TrEMBL [42], KEGG [43], and the NCBI non-redundant protein databases, using BLASTP (E-value < 1E-5). We predicted structural motifs and domains using InterProScan (v5.24) [44] by searching against ProDom [45], PRINTS [46], Pfam [47], SMART [48], and PROSITE [49] databases. Moreover, we performed the Gene Ontology (GO) annotation of predicted genes by searching the corresponding InterProScan entries.

**Phylogenetic analysis and divergence time estimation**

We performed OrthoFinder v2.2.7 [50] to classify the orthogroups of proteins from *A. heterophyllus* and 8 other species of angiosperms comprising *A. altilis* [51], *M. alba*, *M. notabilis* [52], *Ficus erecta* [53], *Ficus carica* [54], *F. hispida*, *Ficus microcarpa* [35], *Cannabis sativa* [55]. We aligned the proteins for each gene within single-copy orthogroups in MUSCLE v3.8.31 [56]. For each gene alignment, we reconstructed a Maximum Likelihood (ML) phylogenetic tree in IQ-TREE v1.6.12 [57] with *C. sativa* as the outgroup. We merged the gene trees to construct the phylogenetic tree using ASTRAL v 5.14.2 [58]. For divergence time estimation, we used BEAST v2.6.2 [59] with one fossil constraint, which was stem of *Artocarpus* (67-64 Ma) [60]. We performed a uniform prior distribution for the fossil calibration, setting 64 Ma as the minimum bound of stem *Artocarpus*. We set the constraint for stem of Moraceae as a secondary calibration based on the age estimate (81.7–93.3 Ma) [61]. We performed a normal distribution for this constraint (mean: 87.5 Ma, Sigma: 2.9). Within BEAST, we performed the Markov chain Monte Carlo for 10,000,000 generations with sampling every 1000 generations. We used CAFÉ v4.2 [62] to calculate the gene family expansions and contractions with *P*-value < 0.01. We then analyzed the gene ontology (GO) enrichment and Kyoto Encyclopedia of Genes and Genomes (KEGG) enrichment for the significantly expanded and contracted genes using the R package ‘clusterProfiler’ [63] against all *A. heterophyllus* genes as background.

**Supplementary references**

1. Doyle JJ, Doyle JL. Isolation of plant DNA from fresh tissue. *Focus* 1990;**12**:13-15.
2. Chin CS, Alexander DH, Marks P *et al.* Nonhybrid, finished microbial genome assemblies from long-read SMRT sequencing data. *Nat Methods.* 2013;**10**:563-569.
3. Chin CS, Peluso P, Sedlazeck FJ *et al.* Phased diploid genome assembly with single-molecule real-time sequencing. *Nat Methods.* 2016;**13**:1050-1054.
4. Myers EW. The fragment assembly string graph. *Bioinformatics* 2005;**21**:79-85.
5. Walker BJ, Abeel T, Shea T *et al.* Pilon: an integrated tool for comprehensive microbial variant detection and genome assembly improvement. *PLoS One* 2014;**9**:e112963.
6. Adey A, Kitzman JO, Burton JN *et al.* In vitro, long-range sequence information for de novo genome assembly via transposase contiguity. *Genome Res.* 2014;**24**:2041-9.
7. Langmead B, Salzberg SL. Fast gapped-read alignment with Bowtie 2. *Nat Methods.* 2012;**9**:357-359.
8. Liu B, Shi Y, Yuan J *et al.* Estimation of genomic characteristics by analyzing *k*-mer frequency in *de novo* genome projects. *arXiv* 2013;1308.
9. Parra G, Bradnam K, Korf I. CEGMA: a pipeline to accurately annotate core genes in eukaryotic genomes. *Bioinformatics* 2007;**23**:1061-1067.
10. Simao FA, Waterhouse RM, Ioannidis P *et al.* BUSCO: assessing genome assembly and annotation completeness with single-copy orthologs. *Bioinformatics* 2015;**31**:3210-3212.
11. Grabherr MG, Haas BJ, Yassour M *et al.* Full-length transcriptome assembly from RNA-Seq data without a reference genome. *Nat Biotechnol.* 2011;**29**:644-652.
12. Kent WJ. BLAT--the BLAST-like alignment tool. *Genome Res.* 2002;**12**:656-664.
13. Trapnell C, Roberts A, Goff L *et al.* Differential gene and transcript expression analysis of RNA-seq experiments with TopHat and Cufflinks. *Nat protoc.* 2012;**7**:562-578.
14. Catchen J, Hohenlohe PA, Bassham S *et al.* Stacks: an analysis tool set for population genomics. *Mol Ecol.* 2013;**22**:3124-3140.
15. Grattapaglia D, Sederoff R. Genetic linkage maps of *Eucalyptus grandis* and *Eucalyptus urophylla* using a pseudo-testcross: mapping strategy and RAPD markers. *Genetics* 1994;**137**:1121-1137.
16. Van Ooijen JW. JoinMap® 4.0: Software for the calculation of genetic linkage maps in experimental populations. Kyazma BV, eds. Wageningen, Netrherlands; 2006.
17. Bao W, Kojima KK, Kohany O. Repbase Update, a database of repetitive elements in eukaryotic genomes. *Mob DNA.* 2015;**6**:11.
18. Tarailo‐Graovac M, Chen N. Using RepeatMasker to identify repetitive elements in genomic sequences. *Curr Protoc Bioinf.* 2009;**25**:4.10.1-4.10.14.
19. Nussbaumer T, Martis MM, Roessner SK *et al.* MIPS PlantsDB: a database framework for comparative plant genome research. *Nucleic Acids Res.* 2013;**41**:1144-1151.
20. Edgar RC, Myers EW. PILER: identification and classification of genomic repeats. *Bioinformatics* 2005;**21 Suppl 1**:i152-158.
21. Price AL, Jones NC, Pevzner PA. *De novo* identification of repeat families in large genomes. *Bioinformatics* 2005;**21 Suppl 1**:i351-358.
22. Xu Z, Wang H. LTR_FINDER: an efficient tool for the prediction of full-length LTR retrotransposons. *Nucleic Acids Res.* 2007;**35**:W265-268.
23. Stanke M, Schoffmann O, Morgenstern B *et al.* Gene prediction in eukaryotes with a generalized hidden Markov model that uses hints from external sources. *BMC Bioinf.* 2006;**7**:62.
24. Majoros WH, Pertea M, Salzberg SL. TigrScan and GlimmerHMM: two open source ab initio eukaryotic gene-finders. *Bioinformatics* 2004;**20**:2878-2879.
25. Korf I. Gene finding in novel genomes. *BMC Bioinf.* 2004;**5**:59.
26. Burge C, Karlin S. Prediction of complete gene structures in human genomic DNA. *J Mol Biol.* 1997;**268**:78-94.
27. Guigó R, Knudsen S, Drake N *et al.* Prediction of gene structure. *J Mol Biol.* 1992;**226**:141-157.
28. Birney E, Clamp M, Durbin R. GeneWise and Genomewise. *Genome Res.* 2004;**14**:988-995.
29. Kaul S, Koo HL, Jenkins J *et al.* Analysis of the genome sequence of the flowering plant *Arabidopsis thaliana*. *Nature* 2000;**408**:796-815.
30. Jaillon O, Aury JM, Noel B *et al.* The grapevine genome sequence suggests ancestral hexaploidization in major angiosperm phyla. *Nature* 2007;463-467.
31. Shulaev V, Sargent DJ, Crowhurst RN *et al.* The genome of woodland strawberry (*Fragaria vesca*). *Nat Genet.* 2011;**43**:109-116.
32. Ming R, VanBuren R, Wai CM *et al.* The pineapple genome and the evolution of CAM photosynthesis. *Nat Genet.* 2015;**47**:1435-1442.
33. Teh BT, Lim K, Yong CH *et al.* The draft genome of tropical fruit durian (*Durio zibethinus*). *Nat Genet.* 2017;**49**:1633-1641.
34. Jiao F, Luo R, Dai X *et al.* Chromosome-level reference genome and population genomic analysis provide insights into the evolution and improvement of domesticated mulberry (*Morus alba*). *Mol plant.* 2020;**13**:1001-1012.
35. Zhang XT, Wang G, Zhang SC *et al.* Genomes of the banyan tree and pollinator wasp provide insights into fig-wasp coevolution. *Cell* 2020;**183**:875-889.
36. Altschul SF, Gish W, Miller W *et al.* Basic local alignment search tool. *J Mol Biol.* 1990;**215**:403-410.
37. Kim D, Pertea G, Trapnell C *et al.* TopHat2: accurate alignment of transcriptomes in the presence of insertions, deletions and gene fusions. *Genome Biol.* 2013;**14**:R36.
38. Trapnell C, Williams BA, Pertea G *et al.* Transcript assembly and quantification by RNA-Seq reveals unannotated transcripts and isoform switching during cell differentiation. *Nat Biotechnol.* 2010;**28**:511-515.
39. Haas BJ, Delcher AL, Mount SM *et al.* Improving the Arabidopsis genome annotation using maximal transcript alignment assemblies. *Nucleic Acids Res.* 2003;**31**:5654-5666.
40. Haas BJ, Salzberg SL, Zhu W *et al.* Automated eukaryotic gene structure annotation using EVidenceModeler and the Program to Assemble Spliced Alignments. *Genome Biol.* 2008;**9**:R7.
41. Schneider M, Tognolli M, Bairoch A. The Swiss-Prot protein knowledgebase and ExPASy: providing the plant community with high quality proteomic data and tools. *Plant Physiol Biochem.* 2004;**42**:1013-1021.
42. The UniProt Consortium. UniProt: a hub for protein information. *Nucleic Acids Res.* 2015;43:D204-212.
43. Kanehisa M, Goto S. KEGG: kyoto encyclopedia of genes and genomes. *Nucleic Acids Res.* 2000;**28**:27-30.
44. Jones P, Binns D, Chang HY *et al.* InterProScan 5: genome-scale protein function classification. *Bioinformatics* 2014;**30**:1236-1240.
45. Bru C, Courcelle E, Carrere S *et al.* The ProDom database of protein domain families: more emphasis on 3D. *Nucleic Acids Res.* 2005;**33**:D212-215.
46. Attwood TK, Beck ME, Bleasby AJ *et al.* PRINTS--a database of protein motif fingerprints. *Nucleic Acids Res.* 1994;**22**:3590-3596.
47. Mistry J, Finn R. Pfam: a domain-centric method for analyzing proteins and proteomes. *Methods Mol Biol.* 2007;**396**:43-58.
48. Schultz J, Milpetz F, Bork P *et al.* SMART, a simple modular architecture research tool: identification of signaling domains. *Proc Natl Acad Sci U S A.* 1998;**95**:5857-5864.
49. Hulo N, Bairoch A, Bulliard V *et al.* The PROSITE database. *Nucleic Acids Res.* 2006;**34**:D227-230.
50. Emms DM, Kelly S. OrthoFinder: solving fundamental biases in whole genome comparisons dramatically improves orthogroup inference accuracy. *Genome Biol.* 2015;**16**:157.
51. Sahu SK, Liu M, Yssel A *et al.* Draft genomes of two *Artocarpus* plants, jackfruit (*A. heterophyllus*) and breadfruit (*A. altilis*). *Genes* 2020;**11**:27.
52. He N, Zhang C, Qi X, *et al*. Draft genome sequence of the mulberry tree *Morus notabilis*. *Nat Commun.* 2013;**4**: 2445.
53. Shirasawa K, Yakushiji H, Nishimura R *et al.* The *Ficus erecta* genome aids *Ceratocystis* canker resistance breeding in common fig (*F. carica*). *Plant J.* 2020;**102**:1313-1322.
54. Mori K; Shirasawa K; Nogata H *et al.* Identification of *RAN1* orthologue associated with sex determination through whole genome sequencing analysis in fig (*Ficus carica* L.). *Sci Rep*. 2017;**7**:41124.
55. van Bakel H, Stout JM, Cote AG *et al*. The draft genome and transcriptome of *Cannabis sativa*. *Genome Biol.* 2011;**12**:R102.
56. Edgar RC. MUSCLE: multiple sequence alignment with high accuracy and high throughput. *Nucleic Acids Res.* 2004;**32**:1792-1797.
57. Nguyen LT, Schmidt HA, von Haeseler A *et al.* IQ-TREE: a fast and effective stochastic algorithm for estimating maximum-likelihood phylogenies. *Mol Biol Evol.* 2015;**32**:268-274.
58. Mirarab S, Warnow T. ASTRAL-II: coalescent-based species tree estimation with many hundreds of taxa and thousands of genes. *Bioinformatics* 2015;**31**:i44-52.
59. Drummond AJ, Suchard MA, Xie D *et al.* Bayesian phylogenetics with BEAUti and the BEAST 1.7. *Mol Biol Evol.* 2012;**29**:1969-1973.
60. Mehrotra RCPU, Bande MB. Fossil woods of Lophopetalum and Artocarpus from the Deccan Intertrappean Beds of Mandla district, Madhya Pradesh, India. *Palaeobotanist* 1984;**32**:310–320.
61. Zhang Q, Onstein RE, Little SA *et al.* Estimating divergence times and ancestral breeding systems in Ficus and Moraceae. *Ann Bot.* 2019;**123**:191-204.
62. De Bie T, Cristianini N, Demuth JP *et al.* CAFE: a computational tool for the study of gene family evolution. *Bioinformatics* 2006;**22**:1269-1271.
63. Yu G, Wang LG, Han Y *et al.* clusterProfiler: an R package for comparing biological themes among gene clusters. *Omics: J Integr Biol.* 2012;**16**:284-287.

**Supplementary Figures**


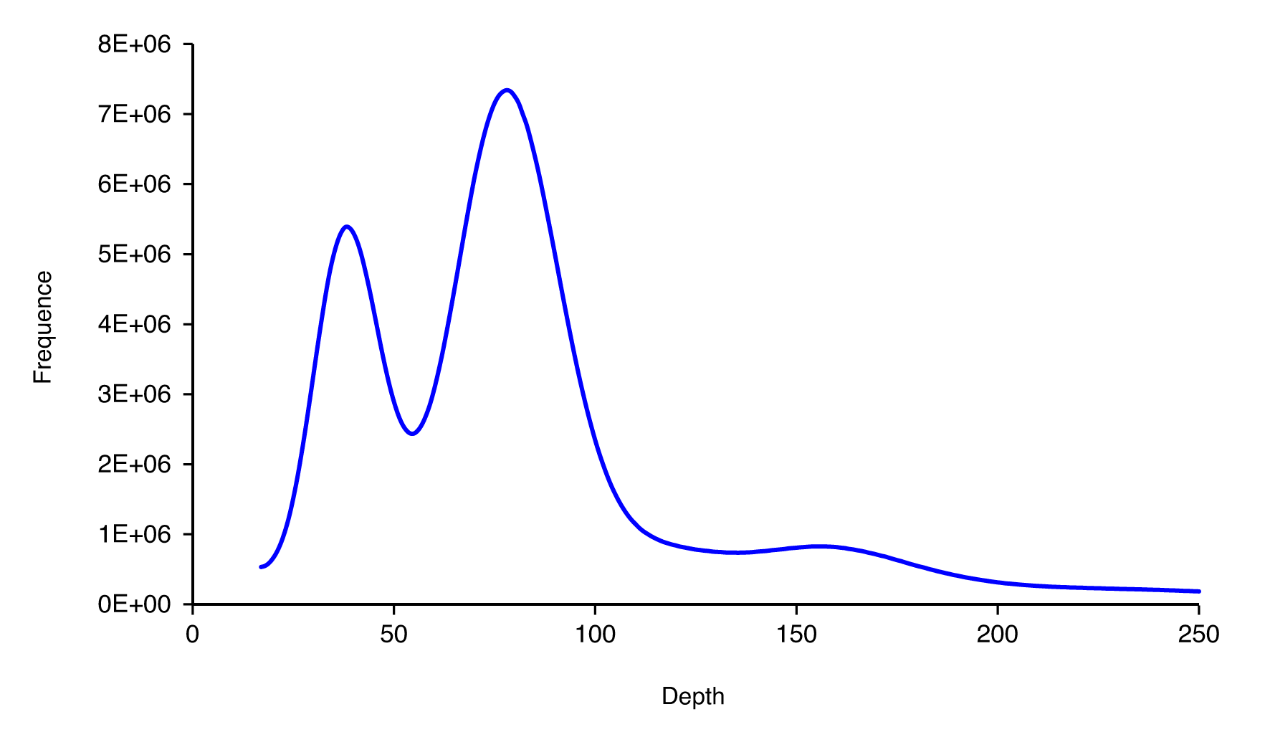


**Supplementary Figure S1.** *K*-mer frequency distributions.

**Supplementary Figure S2.** High-density genetic linkage map of jackfruit.
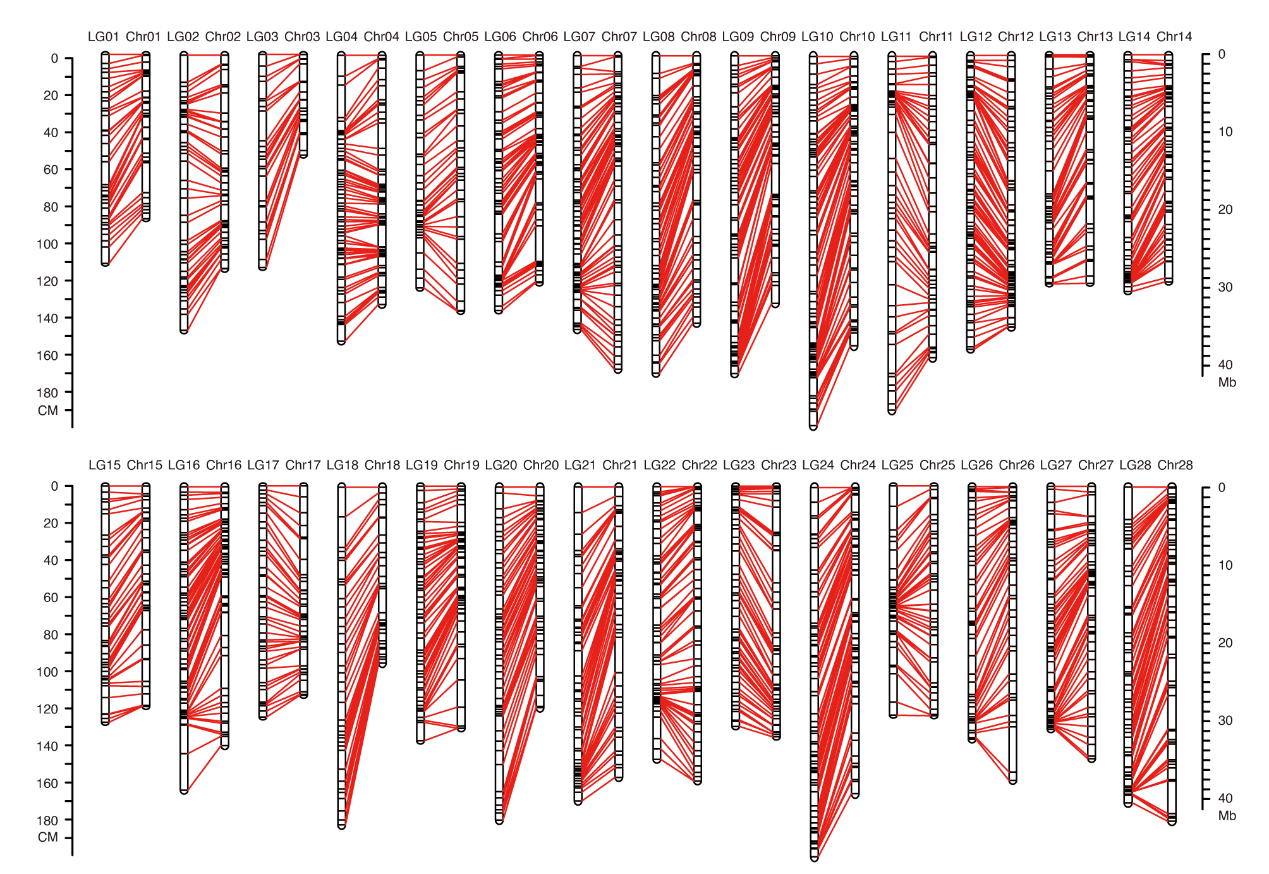


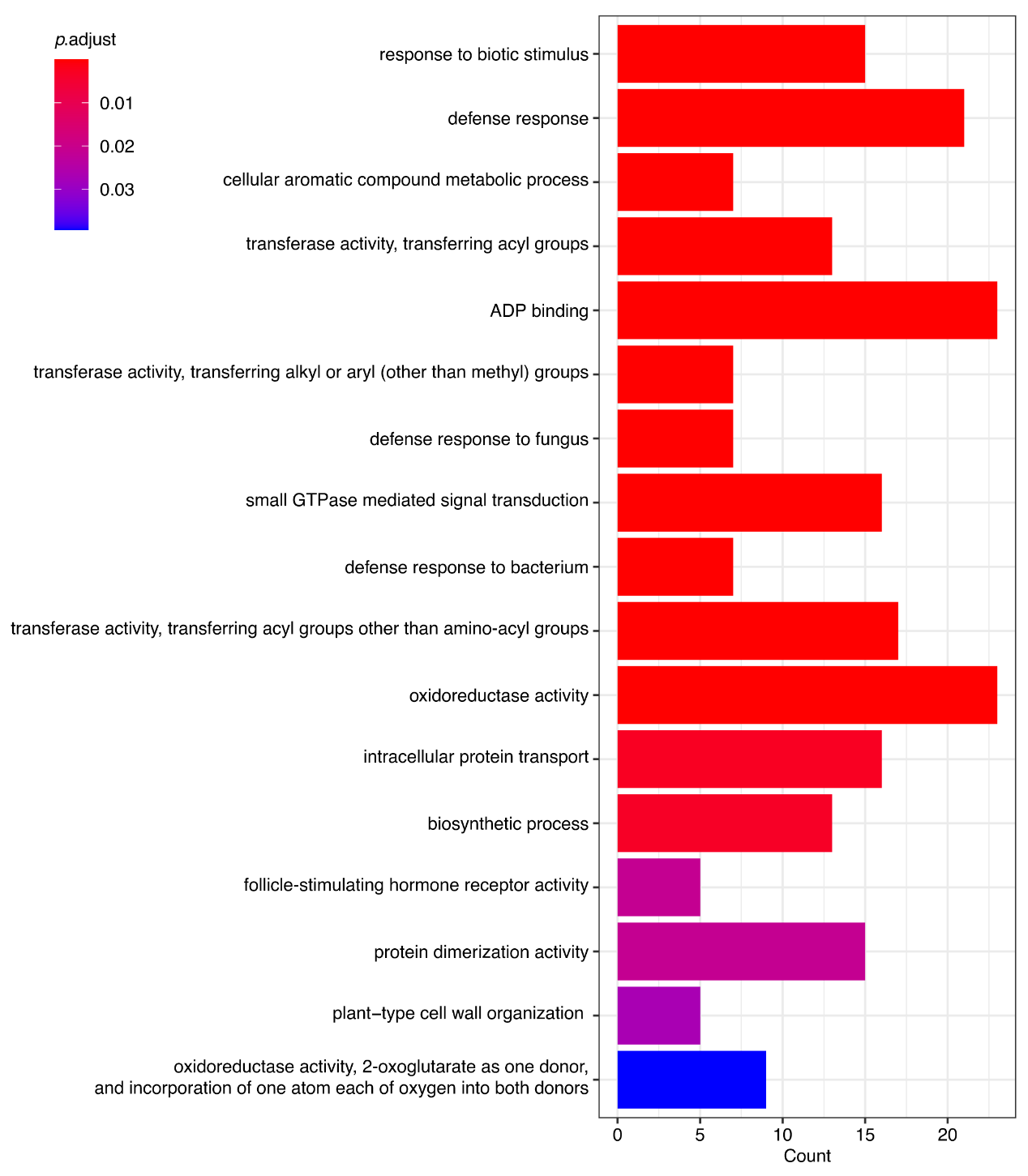
**Supplementary Figure S3.** The GO terms enrichment of the expanded genes from jackfruit.

**Supplementary Figure S4.** The synonymous substitution rate (*Ks*) distributions of different Moraceae species. *Ks* distributions of orthologous gene pairs identified in *Artocarpus*, *Morus*, [*Ficus*](javascript:;) and *Broussonetia* species.
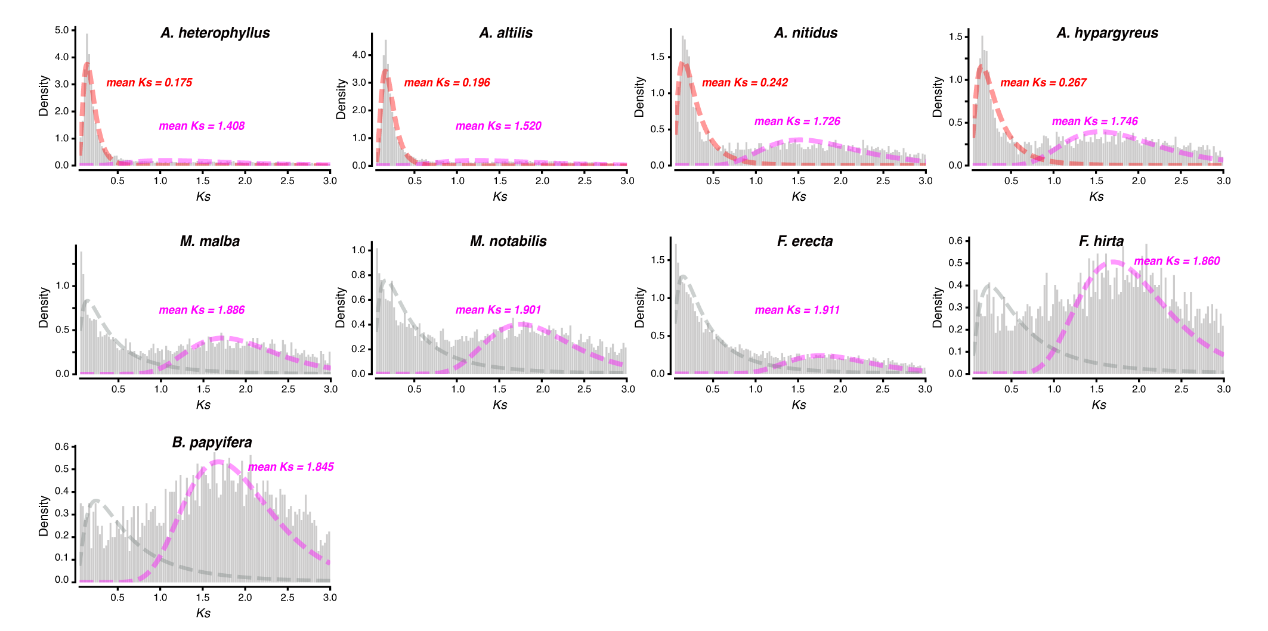


**
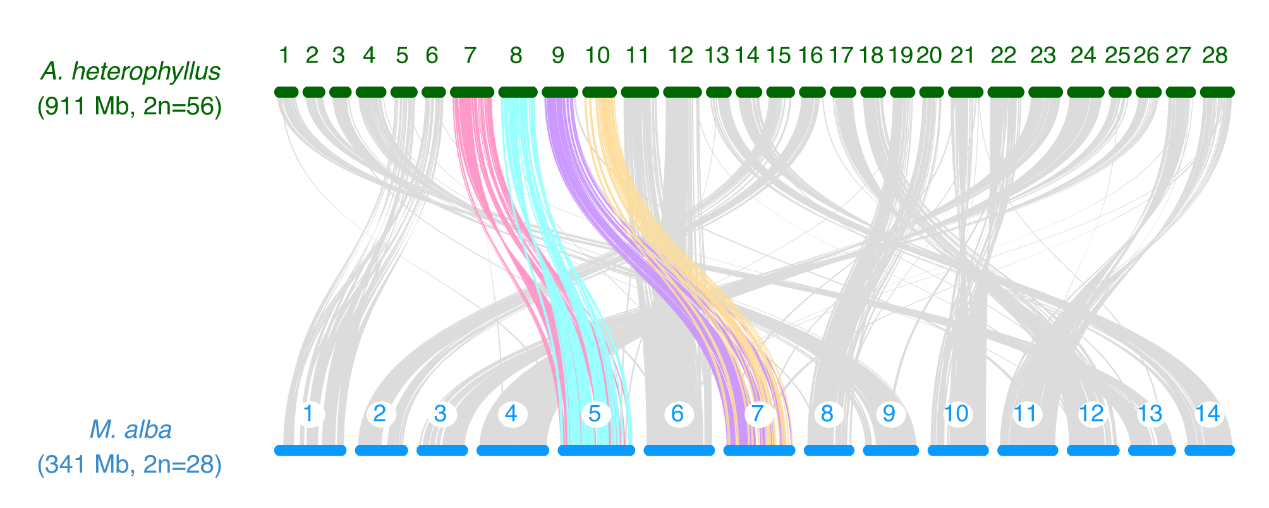
Supplementary Figure S5.** Collinearity result between *A. heterophyllus* and *M. alba*.

**Supplementary Figure S6.** Maximum Likelihood phylogenetic tree of 295 jackfruit accessions. Different colored lables indicate jackfruit individuals from different geographic distributions, while blue lables indicate cempedak individuals. Red rectangles represent the firm flesh individuals, and blue rectangles represent the soft flesh individuals. The light purple dots represent the branch with high bootstrap support (threshold of 0.9).
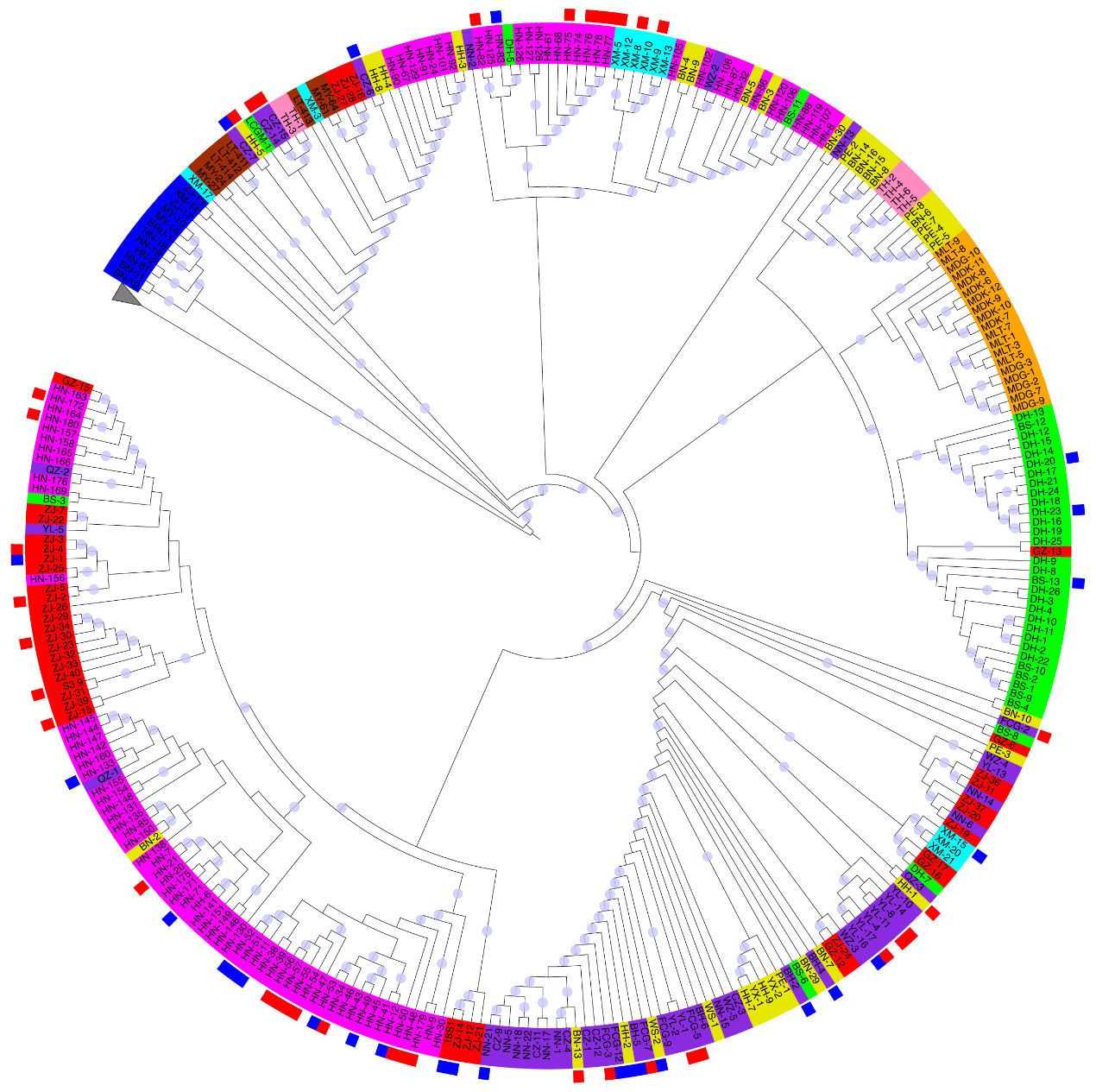


**Supplementary Figure S7.** Population structure of 295 jackfruit accessions. The phylogenetic tree was constructed by merging 19830 parallel trees of every 50-kb nonoverlapping window using Astral. The outgroup comprises 22 individuals of subgenera *Artocarpus* and *Pseudojaca*. Different colors of the branches represent individuals of jackfruit from different groups corresponding to geographic distributions. The population structure of the jackfruit accessions was estimated by running through *K* thresholds range from 2 to 7 using ADMIXTURE.
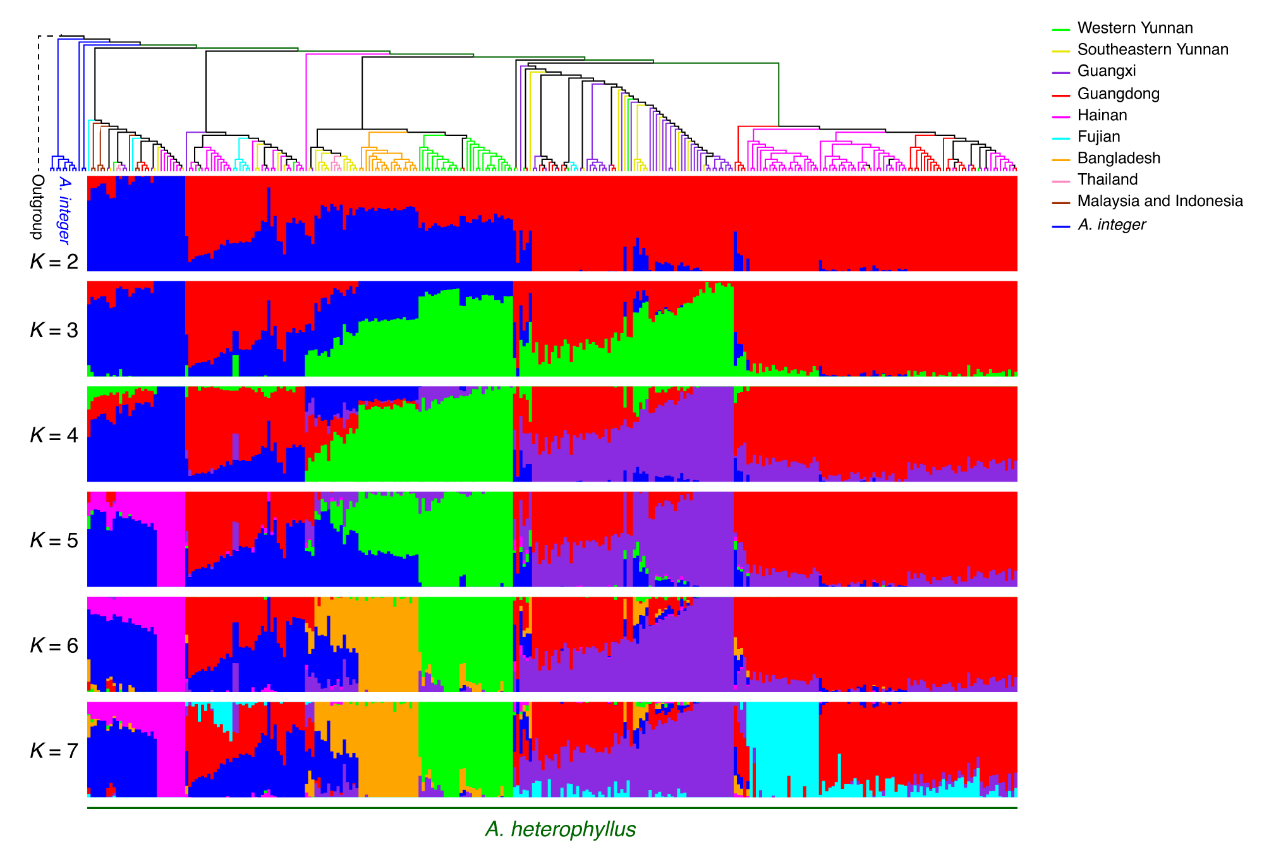


**Supplementary Figure S8.** Identity-by-descent haplotypes shared between jackfruit individuals of different geographic regions.
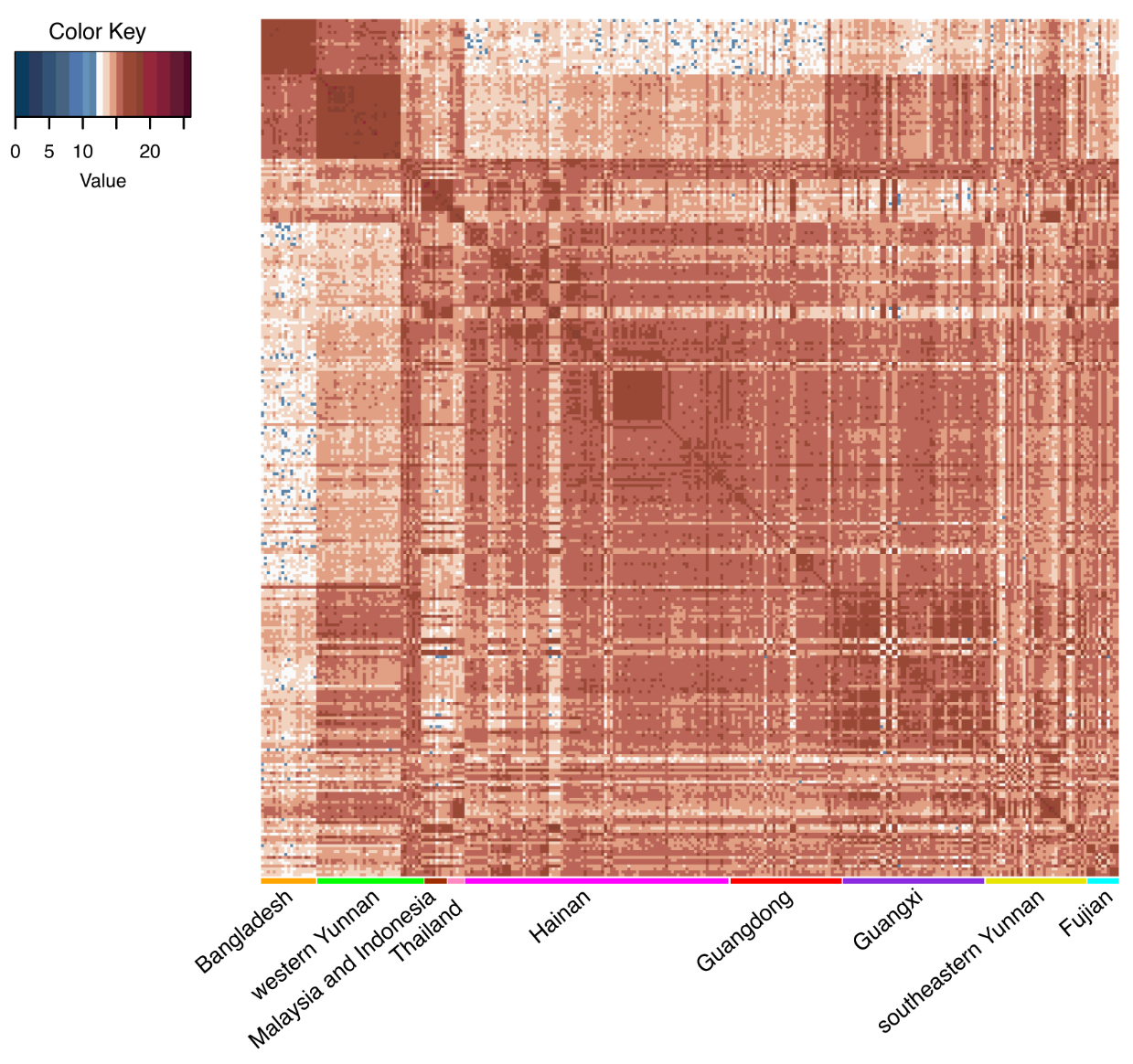


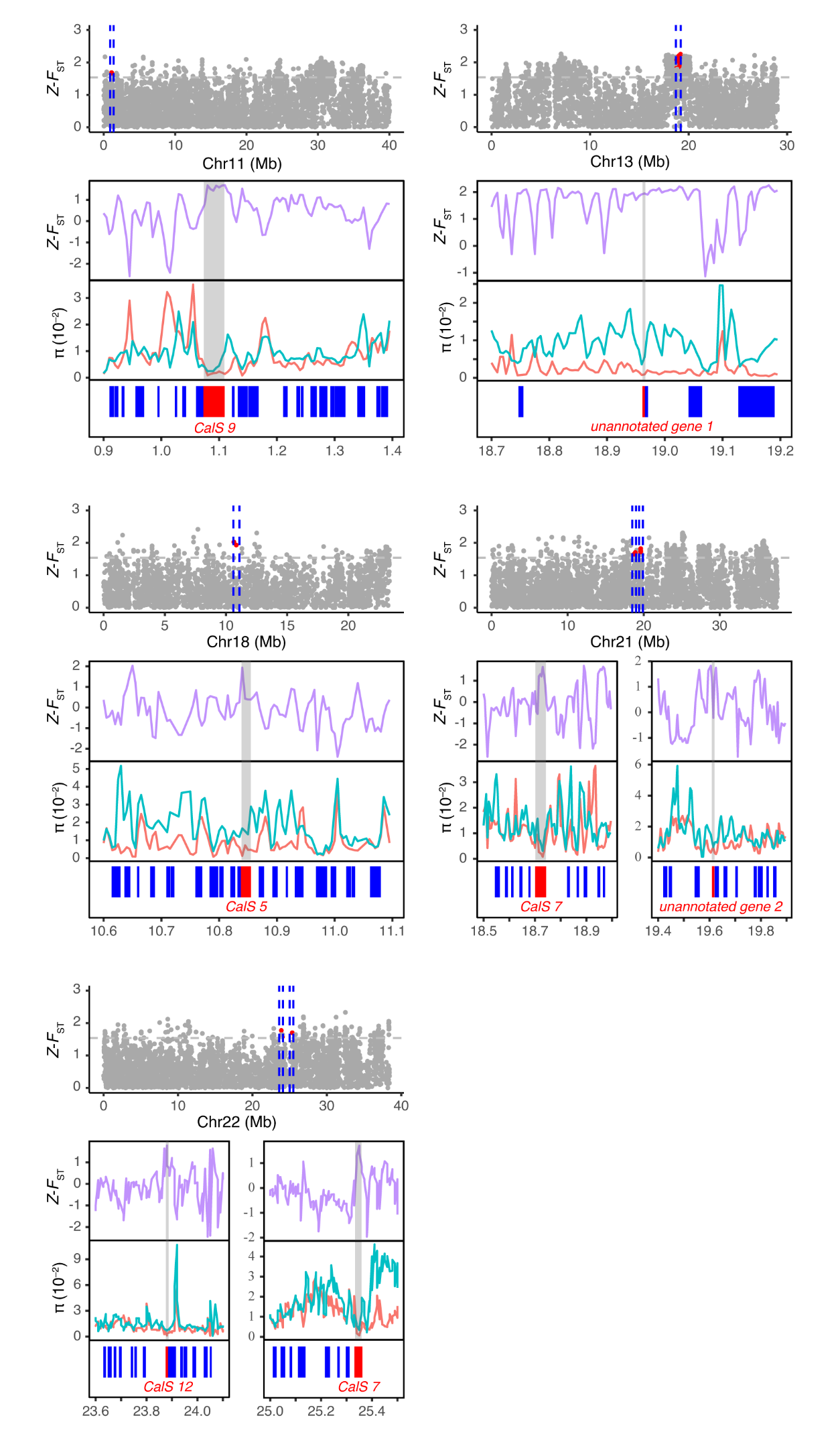


**Supplementary Figure S9.** *Z*-transformed *F*_ST_ and π values of the *CalSs* genes linking region between jackfruit and cempedak. These genes under selection were enriched in callose synthase process. Vertical blue dashed lines represent the 500-kb region around the candidate genes. Red dots indicate the signals of selective sweep in these regions. Red lines indicate the nucleotide diversity of *A. heterophyllus* (AHE group). Green lines indicate the nucleotide diversity of *A. integer* (AIN group).


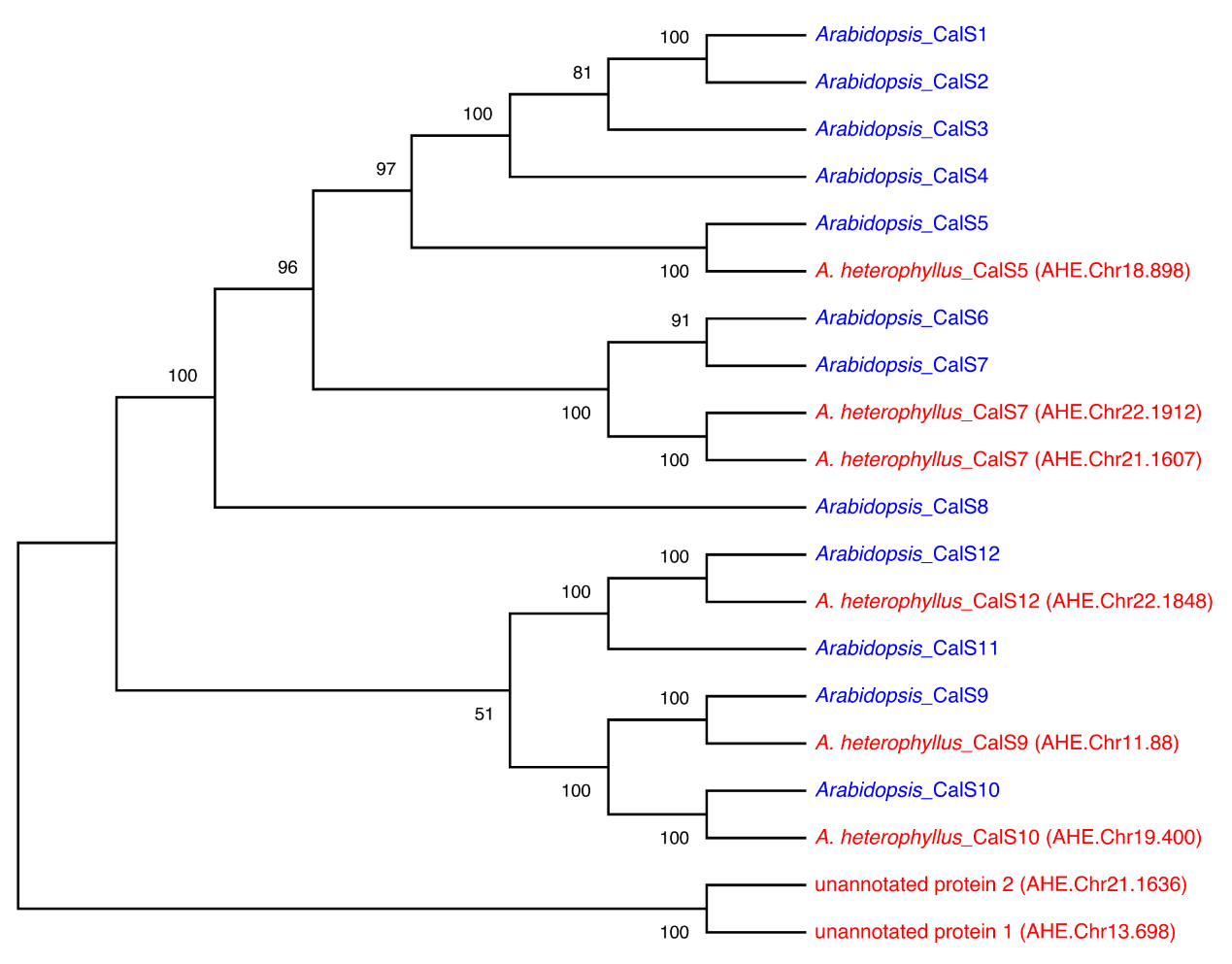
**Supplementary Figure S10.** The neighbor-joining tree of CalSs between *A. heterophyllus* and *A. thaliana.*


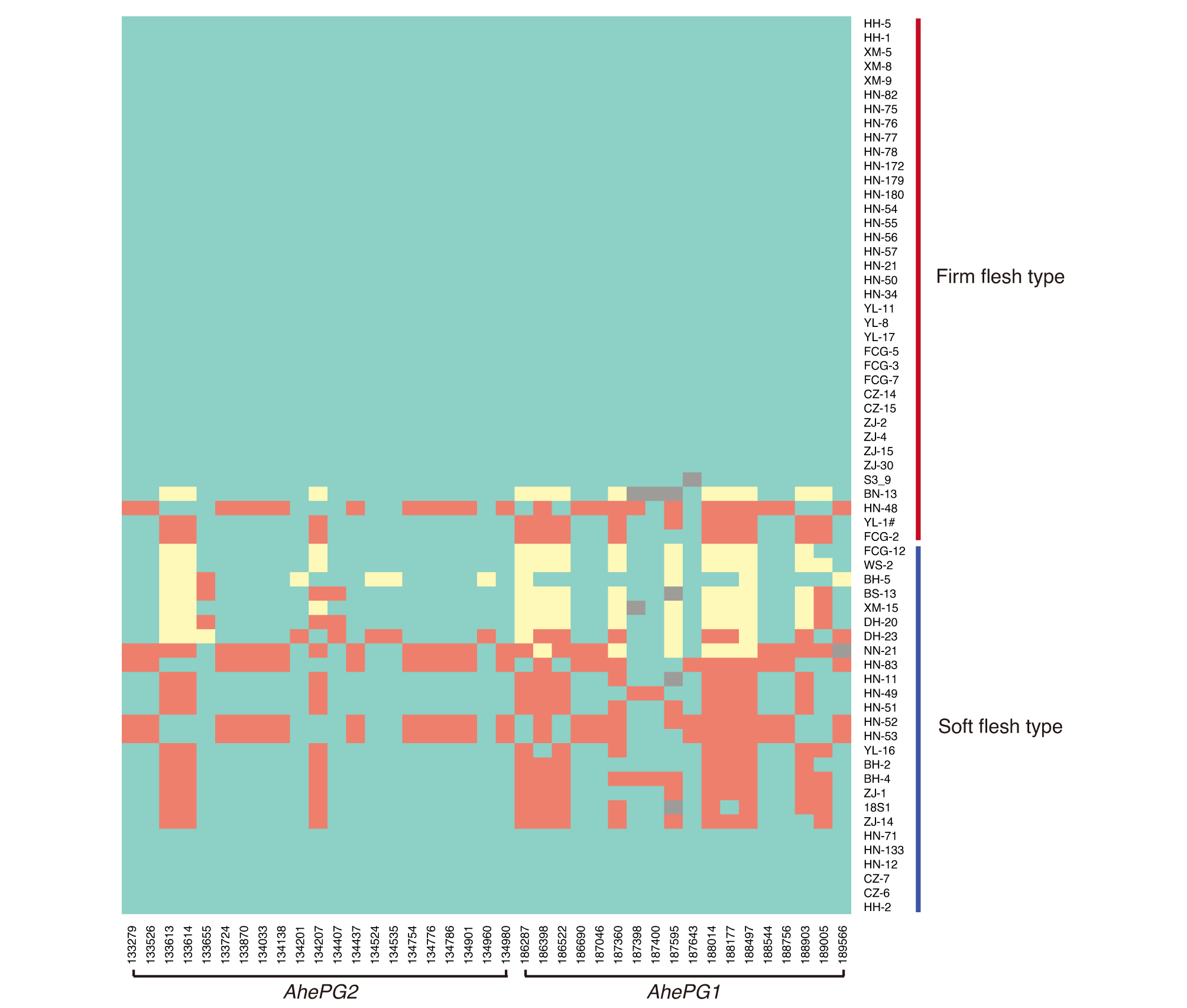
**Supplementary Figure S11.** Haplotype differentiation of *AhePG1* and *AhePG2* between firm and soft flesh types*.* Haplotype distributions suggest that most accessions of firm and soft types of jackfruit can be separated by the SNPs of these *PG* genes associated with fruit texture. Green indicates the homozygous reference alleles, red indicates the heterozygote, yellow indicates the homozygous variant, and gray indicates the missing data.


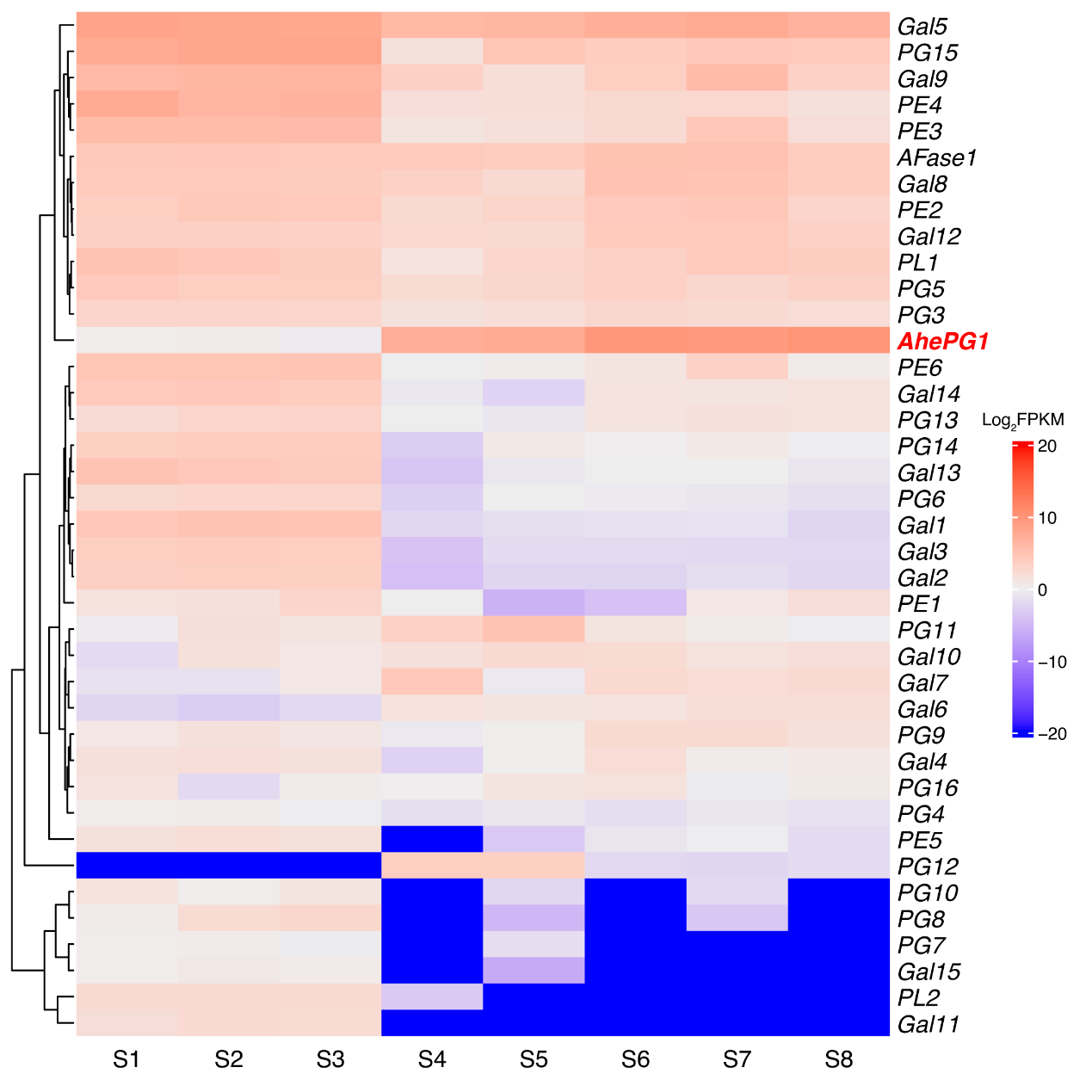
**Supplementary Figure S12.** Gene expression heat map of pectin degradation-related genes during jackfruit softening.

**
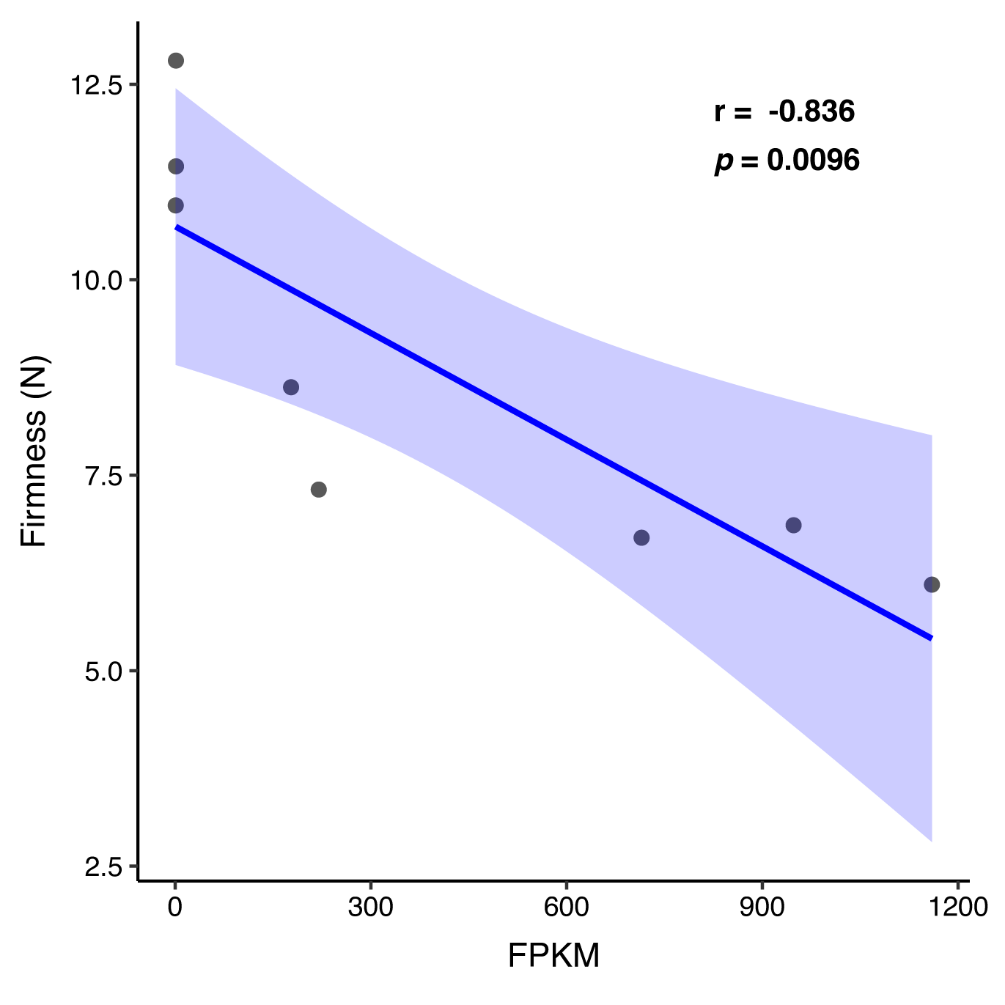
Supplementary Figure S13.** Correlation analysis between the gene expression of *AhePG1* and fruit firmness of jackfruit. The blue shade represents a 95% confidence interval.

**Supplementary Table S1.** Genome survey of jackfruit.

| ***K*-mer** | ***K*-mer**  **Number** | ***K*-mer**  **Depth** | **Genome Size (Mb)** | **Heterozygous Ratio (%)** | **Repeat**  **(%)** |
| --- | --- | --- | --- | --- | --- |
| 17 | 83,818,339,760 | 79 | 1,060.00 | 0.90 | 62.50 |

**Supplementary Table S2.** Statistics of the libraries and sequencing data constructed for the jackfruit genome assembly.

| **Paired-end libraries** | **Insert size (bp)** | **Total data (Gb)** | **Read length (bp)** | **Sequence coverage (×)** |
| --- | --- | --- | --- | --- |
| Illumina reads | 350 | 97.88 | 150 | 92.34 |
| Pacbio reads | - | 111.85 | - | 105.52 |
| 10× Genomics | - | 116.84 | 150 | 110.23 |
| Total | - | 326.57 | - | 308.09 |

**Supplementary Table S3.** Statistics of jackfruit preliminary genome assembly.

| **Parameter** | **length (Mb)** | | **number** | |
| --- | --- | --- | --- | --- |
|  | **Contig** | **Scaffold** | **Contig** | **Scaffold** |
| Total | 982.84 | 985.85 | 1634 | 1185 |
| Max | 13.36 | 13.36 | - | - |
| Number ≥2000 | - | - | 1593 | 1144 |
| N50 | 1.03 | 1.65 | 286 | 183 |
| N60 | 0.85 | 1.32 | 391 | 250 |
| N70 | 0.69 | 1.04 | 519 | 334 |
| N80 | 0.52 | 0.79 | 684 | 442 |
| N90 | 0.36 | 0.51 | 908 | 597 |

**Supplementary Table S4.** The genetic linkage groups of jackfruit.

| **linkage group** | **Total markers** | **Unique loci** | **Distance (cM)** | **Mr mean density (cM)** | **Loci mean density (cM)** | **Anchored scaffolds** | **length (Mb)** | **Loci mean density (Mb)** |
| --- | --- | --- | --- | --- | --- | --- | --- | --- |
| Chr01 | 57 | 36 | 114.721 | 2.01 | 3.19 | 19 | 22.97 | 0.64 |
| Chr02 | 69 | 46 | 147.111 | 2.13 | 3.2 | 19 | 27.33 | 0.59 |
| Chr03 | 34 | 25 | 121.077 | 3.56 | 4.84 | 10 | 14.19 | 0.57 |
| Chr04 | 195 | 68 | 174.5 | 0.89 | 2.57 | 24 | 32.48 | 0.48 |
| Chr05 | 65 | 37 | 123.841 | 1.91 | 3.35 | 27 | 32.81 | 0.89 |
| Chr06 | 93 | 59 | 179.2 | 1.93 | 3.04 | 22 | 29.12 | 0.49 |
| Chr07 | 116 | 69 | 157.867 | 1.36 | 2.29 | 40 | 40.54 | 0.59 |
| Chr08 | 95 | 58 | 180.337 | 1.9 | 3.11 | 37 | 34.73 | 0.6 |
| Chr09 | 187 | 67 | 181.13 | 0.97 | 2.7 | 27 | 32.2 | 0.48 |
| Chr10 | 127 | 77 | 199.235 | 1.57 | 2.59 | 36 | 37.55 | 0.49 |
| Chr11 | 68 | 47 | 190.808 | 2.81 | 4.06 | 37 | 40.06 | 0.85 |
| Chr12 | 238 | 82 | 176.455 | 0.74 | 2.15 | 32 | 35.64 | 0.43 |
| Chr13 | 225 | 55 | 134.117 | 0.6 | 2.44 | 19 | 29.03 | 0.53 |
| Chr14 | 67 | 60 | 128.82 | 1.92 | 2.15 | 32 | 29.09 | 0.48 |
| Chr15 | 56 | 41 | 125.733 | 2.25 | 3.07 | 22 | 30.68 | 0.75 |
| Chr16 | 218 | 64 | 162.848 | 0.75 | 2.54 | 25 | 33.73 | 0.53 |
| Chr17 | 54 | 41 | 133.296 | 2.47 | 3.25 | 20 | 35.57 | 0.87 |
| Chr18 | 45 | 37 | 181.832 | 4.04 | 4.91 | 20 | 23.38 | 0.63 |
| Chr19 | 79 | 56 | 136.339 | 1.73 | 2.43 | 25 | 31.05 | 0.55 |
| Chr20 | 61 | 51 | 184.964 | 3.03 | 3.63 | 29 | 28.75 | 0.56 |
| Chr21 | 74 | 50 | 168.72 | 2.28 | 3.37 | 36 | 37.59 | 0.75 |
| Chr22 | 106 | 60 | 158.825 | 1.5 | 2.65 | 37 | 38.42 | 0.64 |
| Chr23 | 78 | 55 | 130.275 | 1.67 | 2.37 | 26 | 32.72 | 0.59 |
| Chr24 | 98 | 68 | 191.908 | 1.96 | 2.82 | 22 | 36.74 | 0.54 |
| Chr25 | 74 | 43 | 121.812 | 1.65 | 2.83 | 34 | 29.15 | 0.68 |
| Chr26 | 80 | 46 | 164.363 | 2.05 | 3.57 | 33 | 37.83 | 0.82 |
| Chr27 | 100 | 59 | 146.795 | 1.47 | 2.49 | 29 | 34.69 | 0.59 |
| Chr28 | 76 | 66 | 169.46 | 2.23 | 2.57 | 35 | 43.24 | 0.66 |
| Total | **2834** | **1523** | - | - | - | **774** | **911.28** | - |

Note: Total markers, the number of original markers; Unique loci, the number of markers with a unique alignment position; Distance (cM), genetic distance; Mr mean density (cM), the average density of original marker; Loci mean density (cM), the average density of markers with a unique alignment position; Anchored scaffolds, the number of anchored scaffolds; length (Mb), the whole length of anchored scaffolds; Loci mean density (Mb), the average density of anchored markers.

**Supplementary Table S5.** Statistics of jackfruit final genome assembly combined with the genetic linkage groups.

| **Parameter** | **Statistic** | |
| --- | --- | --- |
|  | **All Contig** | **All Scaffold** |
| Total length | 982.84 Mb | 985.63 Mb |
| Longest length | 13.36 Mb | 43.24 Mb |
| N50 | 1.03 Mb | 32.81Mb |
| N90 | 0.36 Mb | 22.97 Mb |
| Total number | 1634 | 482 |
| Number ≥2000 | 1593 | 441 |
|  | **Assembly Contig** | **Assembly Scaffold** |
| Total length | 908.68 Mb | 911.28 Mb |
| Longest length | 13.36 Mb | 43.24 Mb |
| N50 | 1.08 Mb | 33.73 Mb |
| N90 | 0.41 Mb | 28.75 Mb |
| Total number | 1153 | 28 |
| Number ≥2000 | 1153 | 28 |

**Supplementary Table S6.** Assembly and annotation statistics of jackfruit genome.

| **Assembly feature** | **Statistic** |
| --- | --- |
| Estimated genome size (by k-mer analysis) (Mb) | 1060 |
| Number of scaffolds | 482 |
| Scaffold N50 (Mb) | 32.81 |
| Longest scaffold (Mb) | 43.24 |
| Assembled genome size (Mb) | 985.63 |
| Assembly % of genome | 92.98% |
| GC content (%) | 34.86% |
| Repeat region % of assembly | 54.02% |
| Predicted gene models | 41997 |
| Average coding sequence length (bp) | 1190.27 |
| Average exons per gene | 5.1 |
| Mean exon length (bp) | 233.46 |

**Supplementary Table S7.** CEGMA evaluation results for the jackfruit genome.

| **Species** | **Complete** | | **Complete + Partial** | |
| --- | --- | --- | --- | --- |
|  | **# Prots** | **%Completeness** | **# Prots** | **%Completeness** |
| jackfruit | 239 | 96.37 | 242 | 97.58 |

Note: #Prots represent number of 248 ultra conserved CEGs present in genome; %Completeness represent percentage of 248 ultra conserved CEGs.

**Supplementary Table S8.** BUSCO evaluation results for the jackfruit genome.

| **BUSCO notation** | **assessment results** |
| --- | --- |
| Complete BUSCOs | 93.50% |
| Complete and single copy BUSCOs | 58.00% |
| Complete and duplicated BUSCOs | 35.50% |
| Fragmented BUSCOs | 1.00% |
| Missing BUSCOs | 5.50% |
| Total BUSCO groups searched | 1440 |

**Supplementary Table S9.** The coverage assessment of transcriptome data as ESTs.

| **Dataset** | **Number** | **Total length (bp)** | **Sequences Covered by assembly (%)** | **with >90% sequence in one scaffold** | | **with >50% sequence in one scaffold** | |
| --- | --- | --- | --- | --- | --- | --- | --- |
|  |  |  |  | **Number** | **Percent** | **Number** | **Percent** |
| >200bp | 37,688 | 25,283,116 | 98.09 | 34,580 | 91.753 | 36,568 | 97.028 |
| >500bp | 13,148 | 17,604,279 | 98.859 | 12,037 | 91.55 | 12,849 | 97.726 |
| >1000bp | 6,395 | 13,003,307 | 99.312 | 5,971 | 93.37 | 6,302 | 98.546 |
| >2000bp | 2,607 | 7,539,202 | 99.386 | 2,434 | 93.364 | 2,576 | 98.811 |
| >5000bp | 92 | 569,183 | 100 | 81 | 88.043 | 90 | 97.826 |

**Supplementary Table S10.** The evaluation of RNA sequences for genome annotation.

| **Tissues** | **Number of reads** | **Number of mapped reads** | **Ratio（%）** |
| --- | --- | --- | --- |
| Female inflorescences | 41563918 | 34141056 | 82.14 |
| Male inflorescences | 50891992 | 41225068 | 81.01 |
| New leaf bud | 29321902 | 22970487 | 78.34 |
| Tender leaf | 28703394 | 23110159 | 80.51 |
| Old leaf | 36058378 | 28433528 | 78.85 |
| Tender stem | 29259080 | 23172920 | 79.2 |
| Old stem | 46179632 | 37098346 | 80.33 |
| Green fruit of firm flesh type | 30502294 | 24329791 | 79.76 |
| Full-ripening stage of firm flesh type | 53803536 | 44190451 | 82.13 |

**Supplementary Table S11.** Repeat region predictions of the assembled genome of jackfruit.

| **Algorithm** | **Repeat Size (bp)** | **% of genome** |
| --- | --- | --- |
| Tandem repeat finder | 27,736,795 | 2.814 |
| Repeatmasker | 492,760,043 | 49.995 |
| Proteinmask | 179,513,324 | 18.213 |
| Total | 532,435,074 | 54.020 |

**Supplementary Table S12.** Repeat sequences of transposable elements (TEs) in jackfruit.

| **Type** | **RepeatMasker**  **(DNA level)** | | **RepeatProteinMasker (Protein level)** | | **Combined** | |
| --- | --- | --- | --- | --- | --- | --- |
|  | **Len (bp)** | **Per (%)** | **Len (bp)** | **Per (%)** | **Len (bp)** | **Per (%)** |
| DNA transposons | 13,813,748 | 1.402 | 8,684,067 | 0.881 | 21,433,796 | 2.175 |
| Long interspersed nuclear elements (LINE) | 2,605,565 | 0.264 | 2,645,358 | 0.268 | 5,189,236 | 0.526 |
| Short interspersed nuclear elements (SINE) | 67,243 | - | - | - | 67,243 | - |
| Long terminal repeats (LTR) | 471,309,777 | 47.818 | 169,022,704 | 17.149 | 490,401,536 | 49.755 |
| Satellite repeats | 252,732 | 0.026 | - | - | 252,732 | 0.026 |
| Simple repeats | 1,059,180 | 0.107 | - | - | 1,059,180 | 0.107 |
| Unknown | 6,171,169 | 0.626 | - | - | 6,171,169 | 0.626 |
| Total | 492,760,043 | 49.995 | 179,513,324 | 18.213 | 520,076,420 | 52.766 |

**Supplementary Table S13.** Structural annotation of inferred protein-coding genes for jackfruit.

| **Annotation methods** | | **Number of genes** | **Average (bp)** | | | | **Average exon per gene** |
| --- | --- | --- | --- | --- | --- | --- | --- |
|  |  |  | **gene length** | **CDS length** | **exon length** | **intron length** |  |
| *De novo* | Augustus | 55,304 | 2,614.49 | 989.32 | 240.02 | 520.58 | 4.12 |
|  | Geneid | 88,947 | 4,054.21 | 690.16 | 183.09 | 1,214.67 | 3.77 |
|  | SNAP | 64,140 | 3,335.03 | 567.24 | 166.58 | 1,150.71 | 3.41 |
|  | Glimmer | 98,232 | 8,629.98 | 612.68 | 200.33 | 3,895.02 | 3.06 |
|  | Genscan | 59,135 | 9,660.53 | 1,019.73 | 194.86 | 2,041.26 | 5.23 |
| Homolog | *D. zibethinus* | 66,578 | 1,948.63 | 957.69 | 305.55 | 464.30 | 3.13 |
|  | *F. vesca* | 35,510 | 3,704.01 | 1,165.70 | 243.40 | 669.87 | 4.79 |
|  | *V. vinifera* | 44,764 | 2,578.17 | 1,064.47 | 269.35 | 512.77 | 3.95 |
|  | *A. comosus* | 65,519 | 2,061.35 | 796.83 | 263.12 | 623.40 | 3.03 |
|  | *A. thaliana* | 76,915 | 1,688.96 | 825.97 | 303.63 | 501.64 | 2.72 |
|  | *M. alba* | 82,475 | 1,685.10 | 798.98 | 284.00 | 488.67 | 2.81 |
|  | *F. hispida* | 46354 | 2972.76 | 1278.84 | 325.01 | 577.19 | 3.93 |
| RNA-seq | Cufflinks | 79,397 | 6,935.94 | 2,009.26 | 304.69 | 880.65 | 6.59 |
|  | PASA | 44,571 | 3,016.25 | 986.24 | 225.19 | 600.66 | 4.38 |
| Intergration | EVM | 57,113 | 2,817.58 | 993.78 | 230.46 | 550.65 | 4.31 |
|  | PASA-update | 57,024 | 2,782.47 | 991.79 | 231.90 | 546.46 | 4.28 |
|  | Final result | 41,997 | 3,353.70 | 1,190.27 | 233.46 | 527.88 | 5.10 |

**Supplementary Table S14.** Functional annotation of predicted genes for jackfruit.

| **Database** | **Annotated Number** | **Annotated Percent(%)** |
| --- | --- | --- |
| NR | 39211 | 93.4 |
| Swiss-Prot | 32164 | 76.6 |
| KEGG | 30240 | 72.0 |
| InterPro | 40777 | 97.1 |
| Pfam | 31228 | 74.4 |
| GO | 37487 | 89.3 |
| Annotated | 41670 | 99.2 |
| Total | 41997 | - |

**Supplementary Table S15.** Identification of non-coding RNA genes in jackfruit genome.

| **Type** | | **Copy Number** | **Average length (bp)** | **Total length (bp)** | **% of genome** |
| --- | --- | --- | --- | --- | --- |
| miRNA | | 611 | 107.75 | 65,833 | 0.0067 |
| tRNA | | 716 | 75.24 | 53,875 | 0.0055 |
| rRNA | Total rRNA | 1,576 | 321.06 | 505,988 | 0.0513 |
|  | 18S | 439 | 817.78 | 359,004 | 0.0364 |
|  | 28S | 578 | 134.96 | 78,008 | 0.0079 |
|  | 5.8S | 187 | 155.28 | 29,037 | 0.0029 |
|  | 5S | 372 | 107.36 | 39,939 | 0.0041 |
| snRNA | Total snRNA | 1,014 | 108.10 | 109,617 | 0.0111 |
|  | CD-box | 789 | 101.53 | 80,105 | 0.0081 |
|  | HACA-box | 82 | 145.46 | 11,928 | 0.0012 |
|  | splicing | 143 | 122.97 | 17,584 | 0.0018 |

**Supplementary Table S16.** Summary of resequenced accessions of jackfruit and its relatives.

| **ID** | **Lat.** | **Lon.** | **Species** | **MR (%)** | **MD (×)** | **Source** | **Group** |
| --- | --- | --- | --- | --- | --- | --- | --- |
| YX-2 | 23.60 | 101.99 | *A. heterophyllus* | 95.56% | 35.09 | YN | UN |
| YX-1 | 23.60 | 102.00 | *A. heterophyllus* | 94.59% | 29.19 | YN | UN |
| BN-6 | 21.93 | 101.25 | *A. heterophyllus* | 95.39% | 26.97 | YN | UN |
| BN-5 | 21.93 | 101.25 | *A. heterophyllus* | 95.35% | 25.62 | YN | UN |
| BN-7 | 21.93 | 101.25 | *A. heterophyllus* | 94.80% | 27.80 | YN | UN |
| BN-8 | 21.93 | 101.26 | *A. heterophyllus* | 94.14% | 30.02 | YN | UN |
| BN-4 | 21.93 | 101.26 | *A. heterophyllus* | 94.88% | 26.04 | YN | UN |
| BN-2 | 21.93 | 101.26 | *A. heterophyllus* | 95.42% | 26.01 | YN | UN |
| BN-3 | 21.93 | 101.27 | *A. heterophyllus* | 94.44% | 25.87 | YN | UN |
| BN-10 | 21.90 | 101.27 | *A. heterophyllus* | 95.41% | 27.77 | YN | UN |
| BN-9 | 21.91 | 101.27 | *A. heterophyllus* | 95.71% | 34.30 | YN | UN |
| BN-15 | 21.92 | 101.25 | *A. heterophyllus* | 95.69% | 28.40 | YN | UN |
| BN-16 | 21.92 | 101.25 | *A. heterophyllus* | 96.07% | 32.77 | YN | UN |
| BN-14 | 21.92 | 101.25 | *A. heterophyllus* | 95.21% | 26.09 | YN | UN |
| BN-13 | 21.93 | 101.25 | *A. heterophyllus* | 93.61% | 27.18 | YN | FF |
| BN-29 | 22.02 | 100.79 | *A. heterophyllus* | 95.20% | 26.87 | YN | UN |
| BN-30 | 22.02 | 100.79 | *A. heterophyllus* | 95.77% | 26.82 | YN | UN |
| WS-2 | 22.95 | 104.83 | *A. heterophyllus* | 94.98% | 34.44 | YN | SF |
| WS-1 | 22.95 | 104.84 | *A. heterophyllus* | 94.97% | 28.12 | YN | UN |
| PE-7 | 22.80 | 100.98 | *A. heterophyllus* | 95.04% | 29.44 | YN | UN |
| PE-8 | 22.80 | 100.98 | *A. heterophyllus* | 94.26% | 26.35 | YN | UN |
| PE-4 | 22.78 | 100.96 | *A. heterophyllus* | 94.25% | 28.16 | YN | UN |
| PE-5 | 22.78 | 100.97 | *A. heterophyllus* | 94.81% | 29.99 | YN | UN |
| PE-2 | 23.04 | 101.06 | *A. heterophyllus* | 94.93% | 31.98 | YN | UN |
| PE-3 | 23.08 | 101.06 | *A. heterophyllus* | 94.86% | 25.64 | YN | UN |
| PE-1 | 23.43 | 101.68 | *A. heterophyllus* | 94.33% | 26.09 | YN | UN |
| LCGM-1 | 24.12 | 99.37 | *A. heterophyllus* | 95.87% | 27.80 | YN | UN |
| HH-5 | 23.21 | 102.89 | *A. heterophyllus* | 95.94% | 31.41 | YN | FF |
| HH-6 | 23.23 | 102.83 | *A. heterophyllus* | 95.53% | 29.75 | YN | UN |
| HH-8 | 23.33 | 102.50 | *A. heterophyllus* | 94.05% | 33.78 | YN | UN |
| HH-9 | 23.35 | 102.42 | *A. heterophyllus* | 94.82% | 31.49 | YN | UN |
| HH-7 | 23.32 | 102.56 | *A. heterophyllus* | 93.61% | 34.33 | YN | UN |
| HH-3 | 22.57 | 103.88 | *A. heterophyllus* | 95.46% | 41.07 | YN | UN |
| HH-1 | 22.51 | 103.96 | *A. heterophyllus* | 95.41% | 28.99 | YN | FF |
| HH-2 | 22.60 | 103.85 | *A. heterophyllus* | 93.82% | 31.89 | YN | SF |
| HH-4 | 22.99 | 103.40 | *A. heterophyllus* | 95.12% | 26.96 | YN | UN |
| DH-14 | 24.69 | 97.99 | *A. heterophyllus* | 95.08% | 35.59 | YN | UN |
| DH-15 | 24.69 | 97.99 | *A. heterophyllus* | 94.06% | 25.72 | YN | UN |
| DH-13 | 24.69 | 97.98 | *A. heterophyllus* | 94.94% | 35.05 | YN | UN |
| DH-12 | 24.67 | 97.94 | *A. heterophyllus* | 95.18% | 37.68 | YN | UN |
| DH-17 | 24.77 | 98.14 | *A. heterophyllus* | 95.01% | 38.43 | YN | UN |
| DH-16 | 24.76 | 98.14 | *A. heterophyllus* | 94.51% | 40.50 | YN | UN |
| DH-19 | 24.77 | 98.13 | *A. heterophyllus* | 94.59% | 31.35 | YN | UN |
| DH-21 | 24.76 | 98.13 | *A. heterophyllus* | 94.67% | 32.66 | YN | UN |
| DH-18 | 24.77 | 98.13 | *A. heterophyllus* | 95.11% | 54.45 | YN | UN |
| DH-22 | 24.76 | 98.13 | *A. heterophyllus* | 95.24% | 31.71 | YN | UN |
| DH-20 | 24.76 | 98.13 | *A. heterophyllus* | 95.11% | 35.75 | YN | SF |
| DH-23 | 24.76 | 98.13 | *A. heterophyllus* | 94.95% | 33.48 | YN | SF |
| DH-24 | 24.76 | 98.13 | *A. heterophyllus* | 94.55% | 32.13 | YN | UN |
| DH-4 | 23.99 | 97.87 | *A. heterophyllus* | 94.34% | 35.38 | YN | UN |
| DH-1 | 24.01 | 97.85 | *A. heterophyllus* | 95.36% | 41.77 | YN | UN |
| DH-2 | 24.01 | 97.85 | *A. heterophyllus* | 95.07% | 33.54 | YN | UN |
| DH-5 | 24.02 | 97.86 | *A. heterophyllus* | 95.88% | 38.62 | YN | UN |
| DH-3 | 24.00 | 97.86 | *A. heterophyllus* | 95.09% | 40.08 | YN | UN |
| DH-26 | 24.43 | 98.59 | *A. heterophyllus* | 94.57% | 29.42 | YN | UN |
| DH-25 | 24.43 | 98.58 | *A. heterophyllus* | 95.71% | 33.89 | YN | UN |
| DH-7 | 24.18 | 97.80 | *A. heterophyllus* | 95.56% | 32.94 | YN | UN |
| DH-8 | 24.19 | 97.80 | *A. heterophyllus* | 95.41% | 33.40 | YN | UN |
| DH-9 | 24.22 | 97.85 | *A. heterophyllus* | 95.51% | 38.27 | YN | UN |
| DH-11 | 24.25 | 97.88 | *A. heterophyllus* | 94.04% | 33.62 | YN | UN |
| DH-10 | 24.25 | 97.88 | *A. heterophyllus* | 94.39% | 36.21 | YN | UN |
| BS-12 | 24.92 | 98.38 | *A. heterophyllus* | 95.44% | 36.99 | YN | UN |
| BS-1 | 24.97 | 98.88 | *A. heterophyllus* | 95.24% | 33.18 | YN | UN |
| BS-2 | 24.97 | 98.88 | *A. heterophyllus* | 94.97% | 34.26 | YN | UN |
| BS-3 | 24.97 | 98.88 | *A. heterophyllus* | 95.57% | 36.38 | YN | UN |
| BS-4 | 24.97 | 98.88 | *A. heterophyllus* | 96.45% | 39.21 | YN | UN |
| BS-6 | 24.97 | 98.88 | *A. heterophyllus* | 95.57% | 35.61 | YN | UN |
| BS-8 | 24.97 | 98.88 | *A. heterophyllus* | 95.94% | 33.69 | YN | UN |
| BS-9 | 24.97 | 98.88 | *A. heterophyllus* | 95.17% | 32.87 | YN | UN |
| BS-10 | 24.96 | 98.89 | *A. heterophyllus* | 95.30% | 25.87 | YN | UN |
| BS-11 | 24.96 | 98.89 | *A. heterophyllus* | 96.19% | 40.71 | YN | UN |
| BS-13 | 24.92 | 99.43 | *A. heterophyllus* | 95.18% | 32.10 | YN | SF |
| XM-10 | 24.45 | 118.10 | *A. heterophyllus* | 95.90% | 28.88 | FJ | UN |
| XM-12 | 24.45 | 118.10 | *A. heterophyllus* | 96.12% | 32.57 | FJ | UN |
| XM-13 | 24.45 | 118.10 | *A. heterophyllus* | 96.15% | 32.93 | FJ | UN |
| XM-15 | 24.45 | 118.06 | *A. heterophyllus* | 95.66% | 29.38 | FJ | SF |
| XM-17 | 24.45 | 118.06 | *A. heterophyllus* | 96.11% | 34.11 | FJ | UN |
| XM-20 | 24.45 | 118.06 | *A. heterophyllus* | 95.22% | 32.48 | FJ | UN |
| XM-21 | 24.45 | 118.06 | *A. heterophyllus* | 95.45% | 33.96 | FJ | UN |
| XM-3 | 24.45 | 118.10 | *A. heterophyllus* | 95.38% | 28.39 | FJ | UN |
| XM-5 | 24.45 | 118.10 | *A. heterophyllus* | 96.10% | 32.17 | FJ | FF |
| XM-8 | 24.45 | 118.10 | *A. heterophyllus* | 96.23% | 36.07 | FJ | FF |
| XM-9 | 24.45 | 118.10 | *A. heterophyllus* | 96.23% | 35.38 | FJ | FF |
| HN-146 | 19.61 | 110.75 | *A. heterophyllus* | 95.16% | 26.68 | HN | UN |
| HN-147 | 19.61 | 110.75 | *A. heterophyllus* | 95.52% | 32.06 | HN | UN |
| HN-148 | 19.61 | 110.75 | *A. heterophyllus* | 95.47% | 29.83 | HN | UN |
| HN-149 | 19.61 | 110.75 | *A. heterophyllus* | 95.05% | 26.88 | HN | UN |
| HN-141 | 19.54 | 110.75 | *A. heterophyllus* | 95.29% | 28.56 | HN | UN |
| HN-142 | 19.54 | 110.73 | *A. heterophyllus* | 95.56% | 30.95 | HN | UN |
| HN-144 | 19.53 | 110.73 | *A. heterophyllus* | 95.02% | 28.85 | HN | UN |
| HN-145 | 19.53 | 110.73 | *A. heterophyllus* | 95.29% | 30.43 | HN | UN |
| HN-125 | 18.77 | 110.21 | *A. heterophyllus* | 95.36% | 30.29 | HN | UN |
| HN-126 | 18.77 | 110.21 | *A. heterophyllus* | 95.52% | 37.47 | HN | UN |
| HN-127 | 18.77 | 110.22 | *A. heterophyllus* | 95.27% | 29.78 | HN | UN |
| HN-128 | 18.78 | 110.22 | *A. heterophyllus* | 95.28% | 30.95 | HN | UN |
| HN-129 | 18.78 | 110.22 | *A. heterophyllus* | 94.99% | 31.18 | HN | UN |
| HN-130 | 18.80 | 110.30 | *A. heterophyllus* | 95.45% | 29.23 | HN | UN |
| HN-101 | 18.74 | 110.19 | *A. heterophyllus* | 95.27% | 34.02 | HN | UN |
| HN-102 | 18.74 | 110.19 | *A. heterophyllus* | 96.11% | 34.35 | HN | UN |
| HN-105 | 18.74 | 110.19 | *A. heterophyllus* | 96.02% | 27.59 | HN | UN |
| HN-106 | 18.74 | 110.19 | *A. heterophyllus* | 95.53% | 29.12 | HN | UN |
| HN-119 | 18.73 | 110.19 | *A. heterophyllus* | 94.20% | 27.97 | HN | UN |
| HN-120 | 18.73 | 110.19 | *A. heterophyllus* | 95.13% | 34.31 | HN | UN |
| HN-86 | 18.74 | 110.19 | *A. heterophyllus* | 95.49% | 31.78 | HN | UN |
| HN-87 | 18.74 | 110.19 | *A. heterophyllus* | 95.65% | 28.20 | HN | UN |
| HN-88 | 18.74 | 110.19 | *A. heterophyllus* | 95.60% | 42.34 | HN | UN |
| HN-90 | 18.74 | 110.19 | *A. heterophyllus* | 94.89% | 25.37 | HN | UN |
| HN-91 | 18.74 | 110.19 | *A. heterophyllus* | 95.19% | 32.90 | HN | UN |
| HN-92 | 18.74 | 110.19 | *A. heterophyllus* | 95.22% | 35.18 | HN | UN |
| HN-107 | 18.74 | 110.19 | *A. heterophyllus* | 95.13% | 27.21 | HN | UN |
| HN-108 | 18.74 | 110.19 | *A. heterophyllus* | 95.99% | 36.64 | HN | UN |
| HN-82 | 18.73 | 110.23 | *A. heterophyllus* | 95.37% | 32.74 | HN | FF |
| HN-83 | 18.74 | 110.23 | *A. heterophyllus* | 95.35% | 29.07 | HN | SF |
| HN-85 | 18.74 | 110.23 | *A. heterophyllus* | 94.87% | 32.48 | HN | UN |
| HN-67 | 18.31 | 109.33 | *A. heterophyllus* | 95.09% | 29.45 | HN | UN |
| HN-68 | 18.31 | 109.33 | *A. heterophyllus* | 95.12% | 24.44 | HN | UN |
| HN-71 | 18.42 | 109.31 | *A. heterophyllus* | 95.67% | 35.57 | HN | SF |
| HN-74 | 18.39 | 109.32 | *A. heterophyllus* | 95.23% | 33.49 | HN | UN |
| HN-75 | 18.39 | 109.32 | *A. heterophyllus* | 95.24% | 31.86 | HN | FF |
| HN-76 | 18.39 | 109.32 | *A. heterophyllus* | 95.13% | 30.93 | HN | FF |
| HN-77 | 18.40 | 109.31 | *A. heterophyllus* | 95.08% | 29.03 | HN | FF |
| HN-78 | 18.40 | 109.31 | *A. heterophyllus* | 95.11% | 35.14 | HN | FF |
| HN-131 | 19.12 | 110.51 | *A. heterophyllus* | 95.11% | 35.97 | HN | UN |
| HN-133 | 19.12 | 110.51 | *A. heterophyllus* | 95.83% | 37.48 | HN | SF |
| HN-135 | 19.14 | 110.53 | *A. heterophyllus* | 95.62% | 34.59 | HN | UN |
| HN-138 | 19.13 | 110.53 | *A. heterophyllus* | 95.71% | 32.40 | HN | UN |
| HN-61 | 18.74 | 108.86 | *A. heterophyllus* | 94.85% | 28.45 | HN | UN |
| HN-163 | 19.89 | 110.19 | *A. heterophyllus* | 95.16% | 28.41 | HN | UN |
| HN-164 | 19.89 | 110.19 | *A. heterophyllus* | 94.63% | 28.84 | HN | UN |
| HN-165 | 19.89 | 110.19 | *A. heterophyllus* | 95.06% | 31.64 | HN | UN |
| HN-166 | 19.89 | 110.19 | *A. heterophyllus* | 95.40% | 28.52 | HN | UN |
| HN-169 | 19.89 | 110.19 | *A. heterophyllus* | 93.73% | 29.52 | HN | UN |
| HN-171 | 19.88 | 110.19 | *A. heterophyllus* | 95.68% | 33.48 | HN | UN |
| HN-172 | 19.88 | 110.19 | *A. heterophyllus* | 95.58% | 32.05 | HN | FF |
| HN-175 | 19.88 | 110.19 | *A. heterophyllus* | 95.59% | 31.16 | HN | UN |
| HN-154 | 19.72 | 110.32 | *A. heterophyllus* | 95.20% | 28.49 | HN | UN |
| HN-155 | 19.72 | 110.32 | *A. heterophyllus* | 95.56% | 36.23 | HN | UN |
| HN-150 | 19.67 | 110.40 | *A. heterophyllus* | 95.35% | 32.51 | HN | UN |
| HN-179 | 19.98 | 110.32 | *A. heterophyllus* | 93.85% | 30.75 | HN | FF |
| HN-180 | 19.98 | 110.32 | *A. heterophyllus* | 95.18% | 33.35 | HN | FF |
| HN-176 | 19.95 | 110.34 | *A. heterophyllus* | 95.33% | 34.84 | HN | UN |
| HN-151 | 19.70 | 110.35 | *A. heterophyllus* | 95.36% | 30.89 | HN | UN |
| HN-54 | 19.43 | 109.38 | *A. heterophyllus* | 95.24% | 30.14 | HN | FF |
| HN-55 | 19.43 | 109.38 | *A. heterophyllus* | 95.53% | 38.31 | HN | FF |
| HN-56 | 19.43 | 109.38 | *A. heterophyllus* | 95.36% | 29.17 | HN | FF |
| HN-57 | 19.43 | 109.38 | *A. heterophyllus* | 95.47% | 35.29 | HN | FF |
| HN-48 | 19.67 | 109.34 | *A. heterophyllus* | 94.84% | 24.07 | HN | FF |
| HN-11 | 19.51 | 109.49 | *A. heterophyllus* | 94.91% | 32.06 | HN | SF |
| HN-12 | 19.51 | 109.49 | *A. heterophyllus* | 95.06% | 29.54 | HN | SF |
| HN-20 | 19.51 | 109.49 | *A. heterophyllus* | 95.70% | 33.56 | HN | UN |
| HN-21 | 19.51 | 109.49 | *A. heterophyllus* | 94.92% | 33.20 | HN | FF |
| HN-24 | 19.53 | 109.49 | *A. heterophyllus* | 95.17% | 31.76 | HN | UN |
| HN-30 | 19.52 | 109.49 | *A. heterophyllus* | 95.97% | 35.22 | HN | UN |
| HN-32 | 19.52 | 109.50 | *A. heterophyllus* | 95.78% | 34.55 | HN | UN |
| HN-7 | 19.51 | 109.49 | *A. heterophyllus* | 95.29% | 28.71 | HN | UN |
| HN-8 | 19.51 | 109.49 | *A. heterophyllus* | 95.13% | 42.36 | HN | UN |
| HN-9 | 19.51 | 109.49 | *A. heterophyllus* | 95.20% | 28.21 | HN | UN |
| HN-46 | 19.52 | 109.37 | *A. heterophyllus* | 95.28% | 31.12 | HN | UN |
| HN-47 | 19.52 | 109.37 | *A. heterophyllus* | 95.56% | 37.20 | HN | UN |
| HN-49 | 19.51 | 109.38 | *A. heterophyllus* | 95.34% | 26.79 | HN | SF |
| HN-51 | 19.50 | 109.38 | *A. heterophyllus* | 95.46% | 36.33 | HN | SF |
| HN-52 | 19.50 | 109.38 | *A. heterophyllus* | 95.42% | 29.16 | HN | SF |
| HN-53 | 19.50 | 109.38 | *A. heterophyllus* | 95.54% | 35.49 | HN | SF |
| HN-50 | 19.50 | 109.38 | *A. heterophyllus* | 95.55% | 32.73 | HN | FF |
| HN-34 | 19.51 | 109.39 | *A. heterophyllus* | 95.10% | 31.97 | HN | FF |
| HN-38 | 19.52 | 109.38 | *A. heterophyllus* | 95.34% | 31.74 | HN | UN |
| HN-39 | 19.52 | 109.38 | *A. heterophyllus* | 95.42% | 31.87 | HN | UN |
| HN-41 | 19.52 | 109.38 | *A. heterophyllus* | 95.44% | 37.73 | HN | UN |
| HN-43 | 19.52 | 109.38 | *A. heterophyllus* | 95.32% | 33.26 | HN | UN |
| HN-45 | 19.52 | 109.38 | *A. heterophyllus* | 95.27% | 29.81 | HN | UN |
| HN-156 | 19.77 | 110.21 | *A. heterophyllus* | 95.17% | 29.52 | HN | UN |
| HN-157 | 19.77 | 110.21 | *A. heterophyllus* | 95.48% | 34.52 | HN | UN |
| HN-158 | 19.77 | 110.21 | *A. heterophyllus* | 95.45% | 35.47 | HN | UN |
| HN-160 | 19.79 | 110.18 | *A. heterophyllus* | 95.41% | 30.64 | HN | UN |
| YL-1# | 22.69 | 110.19 | *A. heterophyllus* | 95.29% | 30.56 | GX | FF |
| YL-2 | 22.69 | 110.19 | *A. heterophyllus* | 95.06% | 30.12 | GX | UN |
| YL-13 | 22.03 | 110.23 | *A. heterophyllus* | 95.38% | 30.92 | GX | UN |
| YL-14 | 22.03 | 110.23 | *A. heterophyllus* | 95.28% | 28.95 | GX | UN |
| YL-10 | 22.04 | 110.23 | *A. heterophyllus* | 95.73% | 34.70 | GX | UN |
| YL-11 | 22.04 | 110.23 | *A. heterophyllus* | 95.18% | 32.26 | GX | FF |
| YL-4 | 22.21 | 109.96 | *A. heterophyllus* | 94.75% | 27.54 | GX | UN |
| YL-8 | 22.20 | 109.92 | *A. heterophyllus* | 95.47% | 32.45 | GX | FF |
| YL-5 | 22.20 | 109.92 | *A. heterophyllus* | 95.84% | 32.89 | GX | UN |
| WZ-2 | 23.48 | 111.27 | *A. heterophyllus* | 95.95% | 35.29 | GX | UN |
| WZ-4 | 23.48 | 111.30 | *A. heterophyllus* | 95.33% | 30.75 | GX | UN |
| WZ-3 | 23.48 | 111.30 | *A. heterophyllus* | 95.07% | 30.94 | GX | UN |
| WZ-5 | 23.50 | 111.31 | *A. heterophyllus* | 95.71% | 40.34 | GX | UN |
| QZ-2 | 22.46 | 109.10 | *A. heterophyllus* | 95.05% | 34.29 | GX | UN |
| QZ-1 | 22.47 | 109.09 | *A. heterophyllus* | 95.68% | 27.95 | GX | UN |
| QZ-3 | 22.44 | 109.31 | *A. heterophyllus* | 94.07% | 33.35 | GX | UN |
| NN-21 | 22.76 | 108.48 | *A. heterophyllus* | 94.58% | 30.13 | GX | SF |
| NN-22 | 22.08 | 108.49 | *A. heterophyllus* | 94.82% | 34.48 | GX | UN |
| NN-17 | 22.81 | 108.29 | *A. heterophyllus* | 93.82% | 34.23 | GX | UN |
| NN-13 | 22.84 | 108.23 | *A. heterophyllus* | 95.81% | 30.40 | GX | UN |
| NN-1 | 22.84 | 108.28 | *A. heterophyllus* | 94.49% | 33.28 | GX | UN |
| NN-2 | 22.84 | 108.28 | *A. heterophyllus* | 95.73% | 33.00 | GX | UN |
| NN-18 | 22.85 | 108.25 | *A. heterophyllus* | 94.65% | 33.98 | GX | UN |
| NN-14 | 22.83 | 108.18 | *A. heterophyllus* | 95.17% | 34.69 | GX | UN |
| NN-15 | 22.83 | 108.18 | *A. heterophyllus* | 95.04% | 31.12 | GX | UN |
| NN-6 | 22.73 | 108.28 | *A. heterophyllus* | 94.85% | 30.00 | GX | UN |
| NN-5 | 22.73 | 108.28 | *A. heterophyllus* | 94.71% | 31.62 | GX | UN |
| YL-16 | 23.39 | 110.07 | *A. heterophyllus* | 95.41% | 28.94 | GX | SF |
| YL-17 | 23.39 | 110.07 | *A. heterophyllus* | 95.17% | 30.00 | GX | FF |
| CZ-1 | 22.15 | 107.98 | *A. heterophyllus* | 94.65% | 33.31 | GX | UN |
| CZ-3 | 22.16 | 107.97 | *A. heterophyllus* | 95.01% | 34.30 | GX | UN |
| FCG-9 | 21.63 | 108.35 | *A. heterophyllus* | 94.96% | 36.30 | GX | UN |
| FCG-12 | 21.56 | 108.30 | *A. heterophyllus* | 95.06% | 37.25 | GX | SF |
| FCG-5 | 21.52 | 108.14 | *A. heterophyllus* | 95.49% | 35.00 | GX | FF |
| FCG-3 | 21.53 | 108.15 | *A. heterophyllus* | 94.86% | 29.99 | GX | FF |
| FCG-2 | 21.61 | 108.10 | *A. heterophyllus* | 96.36% | 42.10 | GX | FF |
| FCG-7 | 21.59 | 108.13 | *A. heterophyllus* | 94.28% | 35.62 | GX | FF |
| CZ-12 | 22.02 | 106.72 | *A. heterophyllus* | 94.94% | 33.50 | GX | UN |
| CZ-11 | 22.12 | 106.75 | *A. heterophyllus* | 94.41% | 35.04 | GX | UN |
| CZ-9 | 22.12 | 106.74 | *A. heterophyllus* | 94.52% | 32.69 | GX | UN |
| CZ-7 | 22.15 | 107.06 | *A. heterophyllus* | 95.82% | 32.09 | GX | SF |
| CZ-6 | 22.15 | 107.06 | *A. heterophyllus* | 95.53% | 32.61 | GX | SF |
| CZ-4 | 22.43 | 107.53 | *A. heterophyllus* | 93.95% | 33.78 | GX | UN |
| BH-5 | 21.44 | 109.05 | *A. heterophyllus* | 95.22% | 39.55 | GX | SF |
| BH-6 | 21.44 | 109.05 | *A. heterophyllus* | 95.43% | 35.52 | GX | UN |
| BH-2 | 21.48 | 109.11 | *A. heterophyllus* | 95.28% | 36.88 | GX | SF |
| BH-4 | 21.48 | 109.11 | *A. heterophyllus* | 95.09% | 33.52 | GX | SF |
| CZ-14 | 22.00 | 106.69 | *A. heterophyllus* | 95.79% | 36.79 | GX | FF |
| CZ-15 | 22.00 | 106.69 | *A. heterophyllus* | 95.42% | 25.20 | GX | FF |
| ZJ-1 | 20.32 | 110.11 | *A. heterophyllus* | 95.27% | 28.85 | GD | SF |
| ZJ-2 | 20.32 | 110.11 | *A. heterophyllus* | 95.39% | 27.31 | GD | FF |
| ZJ-3 | 20.32 | 110.11 | *A. heterophyllus* | 95.04% | 26.75 | GD | UN |
| ZJ-4 | 20.32 | 110.11 | *A. heterophyllus* | 94.97% | 27.56 | GD | FF |
| ZJ-5 | 20.32 | 110.11 | *A. heterophyllus* | 95.58% | 46.08 | GD | UN |
| ZJ-7 | 20.32 | 110.12 | *A. heterophyllus* | 95.55% | 33.68 | GD | UN |
| ZJ-37 | 21.17 | 110.32 | *A. heterophyllus* | 95.60% | 32.89 | GD | UN |
| ZJ-39 | 21.15 | 110.32 | *A. heterophyllus* | 95.50% | 31.34 | GD | UN |
| ZJ-34 | 21.03 | 110.36 | *A. heterophyllus* | 94.98% | 25.36 | GD | UN |
| ZJ-36 | 21.03 | 110.36 | *A. heterophyllus* | 95.46% | 27.59 | GD | UN |
| ZJ-40 | 21.16 | 110.32 | *A. heterophyllus* | 95.24% | 31.68 | GD | UN |
| 18S1 | 21.16 | 110.30 | *A. heterophyllus* | 96.07% | 32.59 | GD | SF |
| S3_9 | 21.16 | 110.30 | *A. heterophyllus* | 93.63% | 28.45 | GD | FF |
| ZJ-15 | 21.16 | 110.30 | *A. heterophyllus* | 95.24% | 27.29 | GD | FF |
| ZJ-16 | 21.16 | 110.30 | *A. heterophyllus* | 94.98% | 31.38 | GD | UN |
| ZJ-19 | 21.16 | 110.30 | *A. heterophyllus* | 95.83% | 29.51 | GD | UN |
| ZJ-20 | 21.16 | 110.30 | *A. heterophyllus* | 95.29% | 31.17 | GD | UN |
| ZJ-21 | 21.16 | 110.30 | *A. heterophyllus* | 98.88% | 41.27 | GD | UN |
| ZJ-22 | 21.16 | 110.30 | *A. heterophyllus* | 95.53% | 31.84 | GD | UN |
| ZJ-23 | 21.16 | 110.30 | *A. heterophyllus* | 95.13% | 30.23 | GD | UN |
| ZJ-24 | 21.16 | 110.30 | *A. heterophyllus* | 94.74% | 29.00 | GD | UN |
| ZJ-25 | 21.16 | 110.30 | *A. heterophyllus* | 95.12% | 25.41 | GD | UN |
| ZJ-26 | 21.16 | 110.30 | *A. heterophyllus* | 95.23% | 24.25 | GD | UN |
| ZJ-27 | 21.16 | 110.30 | *A. heterophyllus* | 95.12% | 25.92 | GD | UN |
| ZJ-28 | 21.16 | 110.30 | *A. heterophyllus* | 95.07% | 25.89 | GD | UN |
| ZJ-14 | 21.16 | 110.28 | *A. heterophyllus* | 95.24% | 28.70 | GD | SF |
| ZJ-11 | 21.17 | 110.27 | *A. heterophyllus* | 95.24% | 32.24 | GD | UN |
| ZJ-12 | 21.17 | 110.27 | *A. heterophyllus* | 95.70% | 29.89 | GD | UN |
| ZJ-29 | 21.02 | 110.38 | *A. heterophyllus* | 95.15% | 30.14 | GD | UN |
| ZJ-30 | 21.02 | 110.38 | *A. heterophyllus* | 95.62% | 31.14 | GD | FF |
| ZJ-31 | 21.02 | 110.38 | *A. heterophyllus* | 95.42% | 31.44 | GD | UN |
| ZJ-32 | 21.02 | 110.38 | *A. heterophyllus* | 95.31% | 38.00 | GD | UN |
| ZJ-33 | 21.02 | 110.38 | *A. heterophyllus* | 95.71% | 33.91 | GD | UN |
| GZ-17 | 23.18 | 113.35 | *A. heterophyllus* | 95.70% | 34.38 | GD | UN |
| GZ-16 | 23.18 | 113.35 | *A. heterophyllus* | 95.40% | 37.56 | GD | UN |
| GZ-15 | 23.18 | 113.35 | *A. heterophyllus* | 95.07% | 31.34 | GD | UN |
| GZ-13 | 23.18 | 113.35 | *A. heterophyllus* | 95.70% | 32.12 | GD | UN |
| GZ-12 | 23.18 | 113.35 | *A. heterophyllus* | 95.72% | 36.30 | GD | UN |
| GZ-6 | 23.18 | 113.37 | *A. heterophyllus* | 96.70% | 44.70 | GD | UN |
| MLT-7 | 24.92 | 91.94 | *A. heterophyllus* | 95.27% | 31.13 | Ban | UN |
| MLT-8 | 24.92 | 91.94 | *A. heterophyllus* | 95.40% | 33.41 | Ban | UN |
| MLT-9 | 24.92 | 91.94 | *A. heterophyllus* | 95.26% | 29.60 | Ban | UN |
| MLT-1 | 24.26 | 91.88 | *A. heterophyllus* | 95.56% | 32.70 | Ban | UN |
| MLT-3 | 24.26 | 91.88 | *A. heterophyllus* | 95.47% | 32.88 | Ban | UN |
| MLT-5 | 24.26 | 91.88 | *A. heterophyllus* | 95.37% | 32.06 | Ban | UN |
| MDK-10 | 23.88 | 90.26 | *A. heterophyllus* | 95.27% | 34.07 | Ban | UN |
| MDK-11 | 23.88 | 90.26 | *A. heterophyllus* | 95.39% | 29.46 | Ban | UN |
| MDK-12 | 23.88 | 90.26 | *A. heterophyllus* | 95.18% | 28.84 | Ban | UN |
| MDK-6 | 23.88 | 90.26 | *A. heterophyllus* | 95.17% | 25.47 | Ban | UN |
| MDK-7 | 23.88 | 90.26 | *A. heterophyllus* | 94.99% | 24.76 | Ban | UN |
| MDK-8 | 23.88 | 90.26 | *A. heterophyllus* | 95.53% | 32.10 | Ban | UN |
| MDK-9 | 23.88 | 90.26 | *A. heterophyllus* | 95.44% | 30.31 | Ban | UN |
| MDG-1 | 22.45 | 91.77 | *A. heterophyllus* | 95.59% | 33.86 | Ban | UN |
| MDG-2 | 22.45 | 91.77 | *A. heterophyllus* | 95.51% | 30.83 | Ban | UN |
| MDG-3 | 22.45 | 91.77 | *A. heterophyllus* | 95.39% | 29.99 | Ban | UN |
| MDG-10 | 21.40 | 92.01 | *A. heterophyllus* | 95.36% | 30.23 | Ban | UN |
| MDG-7 | 21.40 | 92.01 | *A. heterophyllus* | 95.32% | 35.25 | Ban | UN |
| MDG-9 | 21.40 | 92.01 | *A. heterophyllus* | 95.28% | 33.06 | Ban | UN |
| LT-411 | 1.15 | 113.30 | *A. heterophyllus* | 95.45% | 31.87 | Ind | UN |
| LT-412 | 1.15 | 113.30 | *A. heterophyllus* | 95.58% | 35.76 | Ind | UN |
| LT-413 | 1.12 | 113.27 | *A. heterophyllus* | 96.18% | 29.48 | Ind | UN |
| LT-414 | 1.13 | 113.26 | *A. heterophyllus* | 93.86% | 26.99 | Ind | UN |
| TH-1 | 18.48 | 99.35 | *A. heterophyllus* | 95.27% | 32.00 | Tha | UN |
| TH-2 | 18.47 | 99.35 | *A. heterophyllus* | 95.31% | 29.84 | Tha | UN |
| TH-3 | 18.46 | 99.35 | *A. heterophyllus* | 95.06% | 24.36 | Tha | UN |
| TH-4 | 18.46 | 99.35 | *A. heterophyllus* | 95.22% | 32.91 | Tha | UN |
| TH-5 | 18.40 | 99.31 | *A. heterophyllus* | 95.15% | 28.21 | Tha | UN |
| TH-6 | 18.39 | 99.30 | *A. heterophyllus* | 94.90% | 31.04 | Tha | UN |
| MY-24 | 3.20 | 101.85 | *A. heterophyllus* | 95.47% | 28.19 | Mal | UN |
| MY-27 | 3.20 | 101.85 | *A. heterophyllus* | 96.08% | 39.20 | Mal | UN |
| MY-61 | 3.20 | 101.85 | *A. heterophyllus* | 95.38% | 31.96 | Mal | UN |
| MY-64 | 3.18 | 101.85 | *A. heterophyllus* | 95.12% | 28.67 | Mal | UN |
| BRU-1 | 4.53 | 114.69 | *A. integer* | 86.78% | 33.92 | Bru | UN |
| BN-11 | 21.93 | 101.26 | *A. integer* | 85.90% | 28.10 | YN | UN |
| BN-12 | 21.93 | 101.26 | *A. integer* | 85.85% | 27.21 | YN | UN |
| MY-10 | 3.18 | 101.85 | *A. integer* | 86.12% | 33.05 | Mal | UN |
| MY-14 | 3.18 | 101.85 | *A. integer* | 86.09% | 33.51 | Mal | UN |
| HN-81 | 18.39 | 109.31 | *A. integer* | 86.40% | 31.88 | HN | UN |
| HN-18 | 19.51 | 109.50 | *A. integer* | 87.84% | 35.13 | HN | UN |
| HN-19# | 19.51 | 109.50 | *A. integer* | 87.05% | 38.72 | HN | UN |
| HN-2 | 19.51 | 109.48 | *A. integer* | 86.60% | 31.54 | HN | UN |
| ZJ-13 | 21.17 | 110.27 | *A. integer* | 96.32% | 27.56 | GD | UN |
| ZJ-17 | 21.16 | 110.30 | *A. integer* | 96.67% | 35.71 | GD | UN |
| XM-14# | 24.45 | 118.06 | *A. integer* | 96.53% | 27.72 | FJ | UN |
| HN-1 | 19.51 | 109.49 | *A. hypargyreus* | 58.22% | 28.87 | HN | UN |
| MY-46 | 3.18 | 101.85 | *A. elasticus* | 67.12% | 36.85 | Mal | UN |
| MY-4 | 3.20 | 101.85 | *A. excelsus* | 66.11% | 33.27 | Mal | UN |
| MY-7# | 3.20 | 101.85 | *A. excelsus* | 67.25% | 40.16 | Mal | UN |
| BN-26 | 21.92 | 101.27 | *A. gomezianus* | 62.05% | 32.65 | YN | UN |
| HN-15 | 19.51 | 109.58 | *A. incisa* | 66.97% | 40.43 | HN | UN |
| HN-16 | 19.51 | 109.59 | *A. incisa* | 67.18% | 41.39 | HN | UN |
| MY-37 | 3.20 | 101.85 | *A. lancefolius* | 68.74% | 38.63 | Mal | UN |
| MY-41 | 3.20 | 101.85 | *A. lancefolius* | 68.31% | 35.84 | Mal | UN |
| BN-27 | 21.92 | 101.27 | *A. nanchuanensis* | 58.95% | 29.15 | YN | UN |
| MY-57 | 3.20 | 101.85 | *A. odoratissimus* | 68.89% | 32.68 | Mal | UN |
| MY-58 | 3.20 | 101.84 | *A. odoratissimus* | 68.55% | 34.11 | Mal | UN |
| BN-25 | 21.92 | 101.27 | *A. petelotii* | 58.74% | 30.12 | YN | UN |
| MY-16 | 3.20 | 101.84 | *A. sericicarpus* | 66.48% | 33.38 | Mal | UN |
| MY-21 | 3.20 | 101.84 | *A. sericicarpus* | 67.48% | 39.36 | Mal | UN |
| HN-59 | 18.71 | 108.88 | *A. styracifolius* | 62.75% | 34.92 | HN | UN |
| HN-62 | 18.75 | 108.86 | *A. styracifolius* | 58.84% | 27.53 | HN | UN |
| HN-63 | 18.75 | 108.86 | *A. styracifolius* | 59.41% | 32.66 | HN | UN |
| HN-64 | 18.75 | 108.86 | *A. styracifolius* | 58.30% | 25.94 | HN | UN |
| BN-23 | 21.92 | 101.25 | *A. tonkinensis* | 59.12% | 32.44 | YN | UN |
| HN-17 | 19.52 | 109.50 | *A. tonkinensis* | 60.60% | 35.88 | HN | UN |
| HN-33 | 19.53 | 109.48 | *A. tonkinensis* | 59.57% | 33.72 | HN | UN |

Note: Lat., Latitude; Lon., Longitude; MR, Mapping on reference genome; MD, Average mapping depth; YN, Yunnan, China; FJ, Fujian, China; HN, Hainan, China; GX, Guangxi, China; GD, Guangdong, China; Ban, Bangladesh; Tha, Thailand; Mal, Malaysia; Ind, Indonesia; Bru, Brunei; UN, Unknown; SF, Soft flesh group; FF, Firm flesh group.

**Supplementary Table S17.** Nucleotide diversity of different groups.

| **Groups** | **π** | **Multiple comparison** |
| --- | --- | --- |
| Western Yunnan | 0.005088 | a |
| Southeastern Yunnan | 0.004920 | c |
| Guangxi | 0.004770 | e |
| Guangdong | 0.004714 | f |
| Hainan | 0.004753 | ef |
| Fujian | 0.004855 | d |
| Bangladesh | 0.005112 | a |
| Thailand | 0.004758 | ef |
| Malaysia and Indonesia | 0.004996 | b |

**Supplementary Table S18.** Heatmap showing matrix of pairwise *F*_ST_ between jackfruits of different geographic regions.

| **Groups** | **WY** | **SY** | **GX** | **GD** | **HN** | **FJ** | **Ban** | **Tha** | **MI** |
| --- | --- | --- | --- | --- | --- | --- | --- | --- | --- |
| WY | NA | 0.066 | 0.073 | 0.095 | 0.113 | 0.103 | 0.088 | 0.114 | 0.181 |
| SY | 0.066 | NA | 0.032 | 0.036 | 0.041 | 0.038 | 0.093 | 0.052 | 0.118 |
| GX | 0.073 | 0.032 | NA | 0.036 | 0.061 | 0.065 | 0.124 | 0.127 | 0.186 |
| GD | 0.095 | 0.036 | 0.036 | NA | 0.016 | 0.042 | 0.136 | 0.121 | 0.157 |
| HN | 0.113 | 0.041 | 0.061 | 0.016 | NA | 0.033 | 0.146 | 0.102 | 0.129 |
| FJ | 0.103 | 0.038 | 0.065 | 0.042 | 0.033 | NA | 0.135 | 0.104 | 0.124 |
| Ban | 0.088 | 0.093 | 0.124 | 0.136 | 0.146 | 0.135 | NA | 0.116 | 0.181 |
| Tha | 0.114 | 0.052 | 0.127 | 0.121 | 0.102 | 0.104 | 0.116 | NA | 0.108 |
| MI | 0.181 | 0.118 | 0.186 | 0.157 | 0.129 | 0.124 | 0.181 | 0.108 | NA |

Note: WY, Western Yunnan; SY, Southeastern Yunnan; GX, Guangxi; GD, Guangdong; HN, Hainan; FJ, Fujian; Ban, Bangladesh; Tha, Thailand; MI, Malaysia and Indonesia.

**Supplementary Table S19.** Selection signals between jackfruit and cempedak.

| **Gene ID** | **Annotation** |
| --- | --- |
| *AHE.Chr01.28* | zinc finger CCCH domain-containing protein 38 |
| *AHE.Chr01.30* | hypothetical protein L484_020723 |
| *AHE.Chr01.135* | autophagy-related protein 13b |
| *AHE.Chr01.239* | hypothetical protein L484_006714 |
| *AHE.Chr01.241* | bifunctional 3-dehydroquinate dehydratase/shikimate dehydrogenase, chloroplastic |
| *AHE.Chr01.263* | uncharacterized protein LOC21400717 isoform X1 |
| *AHE.Chr01.292* | small GTPase LIP1 |
| *AHE.Chr01.331* | monooxygenase 2 |
| *AHE.Chr01.559* | hypothetical protein AQUCO_03400189v1 |
| *AHE.Chr01.622* | uncharacterized protein LOC21402729 |
| *AHE.Chr01.664* | transcription factor bHLH18 |
| *AHE.Chr01.679* | uncharacterized protein LOC21391149 |
| *AHE.Chr01.792* | hypothetical protein L484_005916 |
| *AHE.Chr01.793* | peroxidase 51 |
| *AHE.Chr01.794* | peroxidase 51 |
| *AHE.Chr01.801* | molybdate-anion transporter |
| *AHE.Chr01.802* | probable prolyl 4-hydroxylase 9 |
| *AHE.Chr01.828* | molybdate-anion transporter |
| *AHE.Chr01.829* | probable prolyl 4-hydroxylase 9 |
| *AHE.Chr01.830* | protein CONSERVED IN THE GREEN LINEAGE AND DIATOMS 27, chloroplastic |
| *AHE.Chr01.1184* | uncharacterized protein LOC21396432 |
| *AHE.Chr01.1210* | transformation/transcription domain-associated protein isoform X2 |
| *AHE.Chr01.1317* | histidine kinase 1 isoform X2 |
| *AHE.Chr01.1319* | proteasome subunit alpha type-7 |
| *AHE.Chr01.1341* | thaumatin-like protein 1 |
| *AHE.Chr02.7* | #N/A |
| *AHE.Chr02.8* | Nucleotide-binding alpha-beta plait domain containing protein |
| *AHE.Chr02.71* | Chitinase 2 |
| *AHE.Chr02.72* | O-fucosyltransferase 38 isoform X1 |
| *AHE.Chr02.76* | 60S ribosomal protein L7-2 |
| *AHE.Chr02.77* | uncharacterized protein LOC21402982 |
| *AHE.Chr02.137* | protein ACTIVITY OF BC1 COMPLEX KINASE 8, chloroplastic isoform X1 |
| *AHE.Chr02.185* | #N/A |
| *AHE.Chr02.417* | uncharacterized protein LOC21402722 |
| *AHE.Chr02.431* | E3 ubiquitin-protein ligase RING1 |
| *AHE.Chr02.468* | GPI ethanolamine phosphate transferase 2 |
| *AHE.Chr02.765* | Peptidase M24A, methionine aminopeptidase, subfamily |
| *AHE.Chr02.883* | BEL1-like homeodomain protein 2 |
| *AHE.Chr02.964* | BEL1-like homeodomain protein 2 |
| *AHE.Chr02.1007* | DnaJ-like |
| *AHE.Chr02.1106* | transformation/transcription domain-associated protein isoform X2 |
| *AHE.Chr02.1107* | Chalcone-flavanone isomerase family protein |
| *AHE.Chr02.1150* | histidine kinase 1 isoform X2 |
| *AHE.Chr02.1151* | proteasome subunit alpha type-7 |
| *AHE.Chr02.1152* | hypothetical protein L484_026659 |
| *AHE.Chr02.1153* | protein POLLENLESS 3-LIKE 2 |
| *AHE.Chr02.1154* | L-ascorbate peroxidase 3 |
| *AHE.Chr03.95* | DnaJ homolog subfamily C member 13 |
| *AHE.Chr03.123* | uncharacterized protein LOC112095306, partial |
| *AHE.Chr03.164* | uncharacterized protein LOC21404645 |
| *AHE.Chr03.165* | E3 ubiquitin-protein ligase BRE1-like 1 |
| *AHE.Chr03.209* | tRNA wybutosine-synthesizing protein 4 |
| *AHE.Chr03.210* | probable protein phosphatase 2C 6 |
| *AHE.Chr03.319* | LOW QUALITY PROTEIN: protein DETOXIFICATION 51 |
| *AHE.Chr03.564* | serine/threonine-protein kinase ATG1a |
| *AHE.Chr03.732* | Zn(2)-C6 fungal-type DNA-binding domain containing protein |
| *AHE.Chr03.820* | ABC transporter G family member 32 |
| *AHE.Chr03.946* | zinc finger CCCH domain-containing protein 24 |
| *AHE.Chr03.947* | probable E3 ubiquitin-protein ligase LOG2 |
| *AHE.Chr03.1011* | V-type proton ATPase subunit a1 |
| *AHE.Chr04.45* | E3 ubiquitin-protein ligase BRE1-like 1 |
| *AHE.Chr04.46* | autophagy-related protein 3 |
| *AHE.Chr04.69* | polyadenylate-binding protein 1-B-binding protein |
| *AHE.Chr04.70* | uncharacterized protein LOC21404699 |
| *AHE.Chr04.134* | coiled-coil domain-containing protein SCD2 isoform X1 |
| *AHE.Chr04.646* | 1-phosphatidylinositol-3-phosphate 5-kinase FAB1A |
| *AHE.Chr04.708* | cold-responsive protein kinase 1 |
| *AHE.Chr04.709* | #N/A |
| *AHE.Chr04.885* | myb-related protein 308 |
| *AHE.Chr04.894* | exportin-2 |
| *AHE.Chr04.962* | adenylate kinase 4 |
| *AHE.Chr04.963* | uncharacterized protein LOC21407785 |
| *AHE.Chr04.975* | -coumarate-CoA ligase 9 |
| *AHE.Chr04.1010* | lysine-specific demethylase REF6 |
| *AHE.Chr04.1086* | stress response protein NST1 isoform X1 |
| *AHE.Chr04.1297* | #N/A |
| *AHE.Chr04.1383* | probable receptor-like protein kinase At5g24010 |
| *AHE.Chr04.1384* | ferric reduction oxidase 2 |
| *AHE.Chr04.1387* | RAB6A-GEF complex partner protein 1 |
| *AHE.Chr04.1411* | mediator of RNA polymerase II transcription subunit 12 |
| *AHE.Chr04.1416* | uncharacterized protein LOC21399475 |
| *AHE.Chr04.1417* | uncharacterized protein LOC21400292 |
| *AHE.Chr04.1418* | UPF0187 protein At3g61320, chloroplastic |
| *AHE.Chr04.1419* | Dual specificity protein kinase splA |
| *AHE.Chr04.1427* | uncharacterized protein LOC21400315 isoform X1 |
| *AHE.Chr04.1428* | Tetratricopeptide-like helical domain containing protein |
| *AHE.Chr04.1536* | glyoxylate/hydroxypyruvate reductase HPR3 isoform X2 |
| *AHE.Chr04.1570* | #N/A |
| *AHE.Chr04.1571* | protein SDA1 homolog |
| *AHE.Chr04.1601.2* | polycomb group protein EMBRYONIC FLOWER 2 isoform X1 |
| *AHE.Chr04.1665* | protein LIGHT-DEPENDENT SHORT HYPOCOTYLS 10 |
| *AHE.Chr04.1811* | SCY1-like protein 2 |
| *AHE.Chr04.1812.1* | mannan endo-1,4-beta-mannosidase 5 |
| *AHE.Chr05.842* | receptor-like protein Cf-9 isoform X2 |
| *AHE.Chr05.900* | pyrophosphate-energized vacuolar membrane proton pump |
| *AHE.Chr05.901* | LOW QUALITY PROTEIN: arogenate dehydrogenase 1, chloroplastic |
| *AHE.Chr05.902* | #N/A |
| *AHE.Chr05.970* | hypothetical protein G4B88_020056 |
| *AHE.Chr05.1375* | uncharacterized protein LOC21395142 |
| *AHE.Chr05.1387* | gibberellin 2-beta-dioxygenase 8 |
| *AHE.Chr05.1518* | potassium channel SKOR |
| *AHE.Chr05.1777* | probable aldo-keto reductase 2 |
| *AHE.Chr05.1778* | hypothetical protein F0562_000006 |
| *AHE.Chr06.147* | RNA-binding KH domain-containing protein PEPPER |
| *AHE.Chr06.152* | plasma membrane ATPase 4 |
| *AHE.Chr06.242* | villin-4 |
| *AHE.Chr06.254* | ABC transporter I family member 17 |
| *AHE.Chr06.255* | Digestive organ expansion factor-like protein |
| *AHE.Chr06.256* | PREDICTED: 60S acidic ribosomal protein P3-1-like |
| *AHE.Chr06.338* | VIN3-like protein 2 isoform X2 |
| *AHE.Chr06.613* | probable glycosyltransferase At5g20260 |
| *AHE.Chr06.731* | Phospholipase D |
| *AHE.Chr06.797* | uncharacterized protein LOC21389719 |
| *AHE.Chr06.798* | mediator of RNA polymerase II transcription subunit 17 |
| *AHE.Chr06.802* | NADH-cytochrome b5 reductase-like protein |
| *AHE.Chr06.873* | mediator of RNA polymerase II transcription subunit 17 |
| *AHE.Chr06.874* | uncharacterized protein LOC21389719 |
| *AHE.Chr06.920* | calcium-binding protein PBP1 |
| *AHE.Chr06.972* | LOW QUALITY PROTEIN: rRNA biogenesis protein rrp36 |
| *AHE.Chr06.973* | probable transcriptional regulator SLK2 |
| *AHE.Chr06.1092* | trafficking protein particle complex subunit 3 |
| *AHE.Chr06.1320* | uncharacterized protein LOC21395684 |
| *AHE.Chr06.1321* | prohibitin-1, mitochondrial |
| *AHE.Chr06.1322* | hypothetical protein Pyn_02918 |
| *AHE.Chr07.3* | uncharacterized membrane protein At1g16860 |
| *AHE.Chr07.34* | probable inorganic phosphate transporter 1-9 |
| *AHE.Chr07.35* | increased DNA methylation 3 |
| *AHE.Chr07.36* | increased DNA methylation 3 |
| *AHE.Chr07.56* | pyrophosphate--fructose 6-phosphate 1-phosphotransferase subunit alpha |
| *AHE.Chr07.92* | myosin-2 |
| *AHE.Chr07.402* | hypothetical protein F0562_035836 |
| *AHE.Chr07.486* | uncharacterized protein LOC21404056 isoform X2 |
| *AHE.Chr07.488* | cytochrome P450 734A1 |
| *AHE.Chr07.489* | hypothetical protein CUMW_072090 |
| *AHE.Chr07.563* | transmembrane protein 147 |
| *AHE.Chr07.564* | B3 domain-containing protein REM16 |
| *AHE.Chr07.637* | PREDICTED: 26S proteasome non-ATPase regulatory subunit 14 homolog |
| *AHE.Chr07.638* | probable carboxylesterase 2 |
| *AHE.Chr07.639* | succinate dehydrogenase subunit 5, mitochondrial |
| *AHE.Chr07.656* | putative calcium-binding protein CML10 |
| *AHE.Chr07.658* | Parvalbumin |
| *AHE.Chr07.657* | Parvalbumin |
| *AHE.Chr07.659* | putative calcium-binding protein CML10 |
| *AHE.Chr07.730* | putative receptor protein kinase ZmPK1 |
| *AHE.Chr07.775* | heat stress transcription factor B-4 |
| *AHE.Chr07.776* | #N/A |
| *AHE.Chr07.819* | calcium-transporting ATPase 3, endoplasmic reticulum-type |
| *AHE.Chr07.847* | AP2-like ethylene-responsive transcription factor ANT |
| *AHE.Chr07.861.2* | CSC1-like protein RXW8 |
| *AHE.Chr07.862* | uncharacterized protein LOC21390505 |
| *AHE.Chr07.863* | transcription factor RAX3 |
| *AHE.Chr07.869* | homeobox protein knotted-1-like 2 |
| *AHE.Chr07.879* | uncharacterized protein LOC21402474 isoform X2 |
| *AHE.Chr07.880* | #N/A |
| *AHE.Chr07.985* | myosin-15 |
| *AHE.Chr07.1043* | protein disulfide-isomerase |
| *AHE.Chr07.1044* | nitrate regulatory gene2 protein |
| *AHE.Chr07.1112* | uncharacterized protein LOC21384851 |
| *AHE.Chr07.1114* | Polyadenylate-binding protein 3 |
| *AHE.Chr07.1115* | Polyadenylate-binding protein 3 |
| *AHE.Chr07.1116* | hypothetical protein FNV43_RR12561 |
| *AHE.Chr07.1117* | #N/A |
| *AHE.Chr07.1118* | ras-related protein Rab7 |
| *AHE.Chr07.1120* | uncharacterized protein LOC21386290 |
| *AHE.Chr07.1123* | #N/A |
| *AHE.Chr07.1124* | #N/A |
| *AHE.Chr07.1125.1* | probable F-actin-capping protein subunit beta |
| *AHE.Chr07.1236* | 7-deoxyloganetin glucosyltransferase |
| *AHE.Chr07.1266* | Serine/arginine-rich splicing factor 2 |
| *AHE.Chr07.1323* | uncharacterized protein LOC21404368 |
| *AHE.Chr07.1324* | #N/A |
| *AHE.Chr07.1367* | uncharacterized protein LOC21390780 |
| *AHE.Chr07.1389* | transcriptional corepressor LEUNIG isoform X1 |
| *AHE.Chr07.1521* | eukaryotic translation initiation factor 5B |
| *AHE.Chr07.1522* | protein FAM135B isoform X1 |
| *AHE.Chr07.1523* | xylulose kinase |
| *AHE.Chr07.1535* | transcriptional corepressor LEUNIG isoform X1 |
| *AHE.Chr07.1544* | uncharacterized protein LOC21390776 |
| *AHE.Chr07.1567* | COP1-interactive protein 1 |
| *AHE.Chr07.1585* | uncharacterized protein LOC21399481 |
| *AHE.Chr07.1628* | protein ECERIFERUM 1 isoform X1 |
| *AHE.Chr07.1692* | carboxypeptidase SOL1 isoform X1 |
| *AHE.Chr07.1695* | tryptophan aminotransferase-related protein 3 |
| *AHE.Chr07.1696* | E3 ubiquitin-protein ligase RING1-like |
| *AHE.Chr07.1698* | probable choline kinase 2 isoform X1 |
| *AHE.Chr07.1699* | dehydrogenase/reductase SDR family member 12 isoform X1 |
| *AHE.Chr07.1701* | #N/A |
| *AHE.Chr07.1702* | #N/A |
| *AHE.Chr07.1779* | PH, RCC1 and FYVE domains-containing protein 1 |
| *AHE.Chr07.1900* | #N/A |
| *AHE.Chr07.1974* | cytoplasmic tRNA 2-thiolation protein 1 isoform X1 |
| *AHE.Chr07.2048* | uncharacterized protein LOC21391113 |
| *AHE.Chr07.2049* | uncharacterized protein LOC21391112 isoform X2 |
| *AHE.Chr07.2095* | #N/A |
| *AHE.Chr07.2099* | protein SABRE isoform X1 |
| *AHE.Chr07.2142* | ent-kaurenoic acid oxidase 1 |
| *AHE.Chr07.2166* | LOW QUALITY PROTEIN: putative acyl-activating enzyme 19 |
| *AHE.Chr07.2167* | uncharacterized protein LOC21406602 |
| *AHE.Chr07.2168* | BTB/POZ domain-containing protein At5g17580 |
| *AHE.Chr07.2169* | transcription factor bHLH91 |
| *AHE.Chr07.2171.1* | protein transport protein SEC23 |
| *AHE.Chr07.2173* | TIGR00730 family Rossman fold protein |
| *AHE.Chr07.2176* | AT-hook motif nuclear-localized protein 23 |
| *AHE.Chr07.2179* | #N/A |
| *AHE.Chr07.2180* | #N/A |
| *AHE.Chr07.2182* | hypothetical protein L484_023837 |
| *AHE.Chr07.2183* | hypothetical protein L484_023837 |
| *AHE.Chr07.2184* | protein VASCULAR ASSOCIATED DEATH 1, chloroplastic isoform X2 |
| *AHE.Chr07.2277* | exocyst complex component SEC6 |
| *AHE.Chr07.2313* | #N/A |
| *AHE.Chr07.2314* | far upstream element-binding protein 1 isoform X2 |
| *AHE.Chr07.2397* | protein EMBRYONIC FLOWER 1 |
| *AHE.Chr07.2398* | hypothetical protein EZV62_013390 |
| *AHE.Chr07.2476* | uncharacterized protein LOC21408782 |
| *AHE.Chr07.2482* | gibberellin 20 oxidase 3 |
| *AHE.Chr07.2484.1* | Coiled-coil domain-containing protein |
| *AHE.Chr07.2582* | peroxidase 45 |
| *AHE.Chr07.2583* | plasma membrane ATPase 4 |
| *AHE.Chr08.11* | stromal processing peptidase, chloroplastic isoform X1 |
| *AHE.Chr08.52* | AT-hook motif nuclear-localized protein 25 |
| *AHE.Chr08.68* | monosaccharide-sensing protein 2 |
| *AHE.Chr08.69* | protein MULTIPLE CHLOROPLAST DIVISION SITE 1 |
| *AHE.Chr08.72* | dolichyl-diphosphooligosaccharide--protein glycosyltransferase subunit 1A isoform X2 |
| *AHE.Chr08.73* | hypothetical protein I3760_07G133800 |
| *AHE.Chr08.74* | MLO-like protein 9 |
| *AHE.Chr08.75* | hypothetical protein FNV43_RR12098 |
| *AHE.Chr08.383* | CHD3-type chromatin-remodeling factor PICKLE |
| *AHE.Chr08.581* | coenzyme Q-binding protein COQ10 homolog, mitochondrial |
| *AHE.Chr08.582* | Rho-associated protein kinase |
| *AHE.Chr08.689* | uncharacterized protein LOC21392226 |
| *AHE.Chr08.690* | ethylene-responsive transcription factor RAP2-1 |
| *AHE.Chr08.703* | calcium-transporting ATPase 3, endoplasmic reticulum-type |
| *AHE.Chr08.748* | homeobox-leucine zipper protein HAT22 |
| *AHE.Chr08.813* | ATP-dependent zinc metalloprotease FTSH 4, mitochondrial isoform X1 |
| *AHE.Chr08.814* | protein indeterminate-domain 7 |
| *AHE.Chr08.883* | myosin-15 |
| *AHE.Chr08.897* | endoribonuclease Dicer homolog 3 |
| *AHE.Chr08.898* | uncharacterized protein LOC21401591 |
| *AHE.Chr08.992* | uncharacterized protein LOC21387350 isoform X1 |
| *AHE.Chr08.993* | NAD(P)H-quinone oxidoreductase subunit M, chloroplastic |
| *AHE.Chr08.1009* | hypothetical protein FNV43_RR25894 |
| *AHE.Chr08.1010* | cytochrome P450 71B37 |
| *AHE.Chr08.1172* | uncharacterized protein LOC21394875 isoform X2 |
| *AHE.Chr08.1489* | transcription initiation factor IIA subunit 2-like |
| *AHE.Chr08.1490* | E3 ubiquitin-protein ligase UPL2 |
| *AHE.Chr08.1495* | vacuolar protein sorting-associated protein 45 homolog isoform X2 |
| *AHE.Chr08.1677* | rho GTPase-activating protein REN1 |
| *AHE.Chr08.1768* | Nitrate transporter 1.5 |
| *AHE.Chr08.1769* | NADH dehydrogenase |
| *AHE.Chr08.1773* | sister chromatid cohesion protein DCC1 |
| *AHE.Chr08.1774* | sister chromatid cohesion protein DCC1 |
| *AHE.Chr08.1775* | PC-Esterase |
| *AHE.Chr08.1779* | DNA replication ATP-dependent helicase/nuclease DNA2 isoform X2 |
| *AHE.Chr08.1780* | DNA replication ATP-dependent helicase/nuclease DNA2 |
| *AHE.Chr08.1781* | endoglucanase 14-like |
| *AHE.Chr08.1782* | ribose-phosphate pyrophosphokinase 1 |
| *AHE.Chr08.1786* | RING-H2 finger protein ATL52 |
| *AHE.Chr08.1787* | uncharacterized protein LOC21406593 isoform X2 |
| *AHE.Chr08.1788* | #N/A |
| *AHE.Chr08.1845* | probable serine/threonine-protein kinase At1g54610 |
| *AHE.Chr08.1853* | S ribosomal protein L3-1, chloroplastic |
| *AHE.Chr08.1854* | chromo domain protein LHP1 |
| *AHE.Chr08.1924* | casein kinase II subunit beta-1 |
| *AHE.Chr08.1925* | uncharacterized protein At1g28695 |
| *AHE.Chr08.1985* | hypothetical protein, partial |
| *AHE.Chr08.2121* | LOW QUALITY PROTEIN: ER membrane protein complex subunit 1 |
| *AHE.Chr08.2123.1* | hypothetical protein L484_007254 |
| *AHE.Chr08.2158_AHE.Chr08.2157* | protein ANTI-SILENCING 1 isoform X2 |
| *AHE.Chr08.2160* | pentatricopeptide repeat-containing protein At1g19720 |
| *AHE.Chr08.2221* | protein arginine N-methyltransferase 1.5 |
| *AHE.Chr08.2222* | protein starmaker |
| *AHE.Chr08.2226* | Ubiquitinyl hydrolase |
| *AHE.Chr08.2291_AHE.Chr08.2290* | probable cyclic nucleotide-gated ion channel 14 |
| *AHE.Chr08.2312* | proteasome subunit beta type-6 |
| *AHE.Chr08.2324* | cannabidiolic acid synthase-like |
| *AHE.Chr09.78* | uncharacterized protein LOC21397028 |
| *AHE.Chr09.79* | WD repeat-containing protein 6 |
| *AHE.Chr09.80* | uncharacterized protein LOC21397028 |
| *AHE.Chr09.81* | autophagy-related protein 8i |
| *AHE.Chr09.84* | Plant peroxidase |
| *AHE.Chr09.85* | hypothetical protein |
| *AHE.Chr09.143* | putative glutamyl endopeptidase |
| *AHE.Chr09.144* | probable glutamyl endopeptidase, chloroplastic isoform X2 |
| *AHE.Chr09.160* | uncharacterized protein LOC21393080 |
| *AHE.Chr09.170* | transcription factor MYB12 |
| *AHE.Chr09.171* | probable protein disulfide-isomerase A6 |
| *AHE.Chr09.172* | uncharacterized protein LOC21390849 isoform X2 |
| *AHE.Chr09.597* | hypothetical protein L484_005652 |
| *AHE.Chr09.770* | NHL repeat-containing protein 2 |
| *AHE.Chr09.947* | Bromodomain and PHD finger-containing protein 3 |
| *AHE.Chr09.994* | AP-1 complex subunit sigma-2 |
| *AHE.Chr09.996* | phototropin-2 isoform X1 |
| *AHE.Chr09.1091* | nuclear transcription factor Y subunit B-5 |
| *AHE.Chr09.1092* | potassium transporter 5 isoform X1 |
| *AHE.Chr09.1218* | TATA element modulatory factor isoform X2 |
| *AHE.Chr09.1264* | probable protein phosphatase 2C 63 |
| *AHE.Chr09.1516* | phospholipase A-2-activating protein |
| *AHE.Chr09.1869* | calmodulin-binding transcription activator 2 |
| *AHE.Chr09.1973* | uncharacterized protein At4g06598 |
| *AHE.Chr09.2025* | probable polyamine transporter At3g13620 |
| *AHE.Chr09.2026* | homogentisate solanesyltransferase, chloroplastic-like |
| *AHE.Chr09.2043* | MADS-box transcription factor |
| *AHE.Chr10.15* | vacuolar protein sorting-associated protein 51 homolog |
| *AHE.Chr10.16* | bZIP transcription factor 53 |
| *AHE.Chr10.17* | Calvin cycle protein |
| *AHE.Chr10.18* | unnamed protein product, partial |
| *AHE.Chr10.49* | DUF724 domain-containing protein 2 isoform X2 |
| *AHE.Chr10.145* | Serine/threonine protein kinase |
| *AHE.Chr10.180* | auxin-responsive protein SAUR21 |
| *AHE.Chr10.223* | probable amidase At4g34880 |
| *AHE.Chr10.293* | mediator of RNA polymerase II transcription subunit 33B |
| *AHE.Chr10.295* | Protein PALE CRESS |
| *AHE.Chr10.297* | agamous-like MADS-box protein AGL103 |
| *AHE.Chr10.298* | isoamylase 2, chloroplastic isoform X2 |
| *AHE.Chr10.440* | #N/A |
| *AHE.Chr10.678* | desumoylating isopeptidase 1 isoform X2 |
| *AHE.Chr10.826* | nucleolar complex protein 4 homolog |
| *AHE.Chr10.827* | nucleolar complex protein 4 homolog |
| *AHE.Chr10.828* | alpha-1,3-mannosyl-glycoprotein 2-beta-N-acetylglucosaminyltransferase isoform X1 |
| *AHE.Chr10.829* | actin cytoskeleton-regulatory complex protein PAN1 isoform X1 |
| *AHE.Chr10.1031* | uncharacterized protein LOC21392403 |
| *AHE.Chr10.1201* | E3 ubiquitin-protein ligase MBR2 |
| *AHE.Chr10.1220* | UDP-glucose flavonoid 3-O-glucosyltransferase 7 |
| *AHE.Chr10.1280* | serine/threonine-protein kinase SMG1 |
| *AHE.Chr10.1355* | calmodulin-like protein 1 |
| *AHE.Chr10.1356* | LOW QUALITY PROTEIN: protein disulfide-isomerase SCO2 |
| *AHE.Chr10.1363* | LAG1 longevity assurance homolog 2 |
| *AHE.Chr10.1448* | uncharacterized protein LOC21401528 isoform X2 |
| *AHE.Chr10.1482* | hypothetical protein L484_008434 |
| *AHE.Chr10.1535* | UDP-glucose:glycoprotein glucosyltransferase isoform X2 |
| *AHE.Chr10.1564* | coatomer subunit alpha-2 |
| *AHE.Chr10.1740* | beta carbonic anhydrase 5, chloroplastic isoform X1 |
| *AHE.Chr10.1871* | protein RETICULATA-RELATED 1, chloroplastic |
| *AHE.Chr10.1894.1* | pentatricopeptide repeat-containing protein At3g18110, chloroplastic isoform X1 |
| *AHE.Chr10.1898* | BRASSINOSTEROID INSENSITIVE 1-associated receptor kinase 1 |
| *AHE.Chr10.1908* | proteasome subunit alpha type-4 |
| *AHE.Chr10.1929.1* | Mini-chromosome maintenance protein |
| *AHE.Chr10.1967* | nuclear pore complex protein NUP214 |
| *AHE.Chr10.2075* | pentatricopeptide repeat-containing protein At5g66520-like |
| *AHE.Chr10.2076* | serine/threonine-protein phosphatase PP2A-4 catalytic subunit |
| *AHE.Chr11.17.2* | E3 ubiquitin-protein ligase CHFR |
| *AHE.Chr11.88* | callose synthase 9 isoform X2 |
| *AHE.Chr11.415* | probable leucine-rich repeat receptor-like protein kinase At5g63930 |
| *AHE.Chr11.416* | pentatricopeptide repeat-containing protein At5g18390, mitochondrial |
| *AHE.Chr11.417* | pentatricopeptide repeat-containing protein At5g18390, mitochondrial |
| *AHE.Chr11.418* | germin-like protein subfamily 1 member 17 |
| *AHE.Chr11.419* | germin-like protein subfamily 1 member 17 |
| *AHE.Chr11.672.1* | helicase protein MOM1 isoform X2 |
| *AHE.Chr11.673_AHE.Chr11.672* | helicase protein MOM1 isoform X2 |
| *AHE.Chr11.869* | probable protein S-acyltransferase 15 |
| *AHE.Chr11.870* | 17.4 kDa class I heat shock protein |
| *AHE.Chr11.985* | E3 ubiquitin-protein ligase SDIR1 isoform X2 |
| *AHE.Chr11.1042* | protein DAMAGED DNA-BINDING 2 |
| *AHE.Chr11.1043* | ABC transporter G family member 31 isoform X1 |
| *AHE.Chr11.1044* | 3-oxo-Delta(4,5)-steroid 5-beta-reductase |
| *AHE.Chr11.1056* | Phosphoinositide phospholipase C 2 |
| *AHE.Chr11.1057* | phosphoinositide phospholipase C 2 isoform X2 |
| *AHE.Chr11.1111* | LRR repeats and ubiquitin-like domain-containing protein At2g30105 |
| *AHE.Chr11.1113* | ubiquitin-activating enzyme E1 1 |
| *AHE.Chr11.1125* | transcription initiation factor TFIID subunit 15b-like isoform X1 |
| *AHE.Chr11.1126* | transcription initiation factor TFIID subunit 15b |
| *AHE.Chr11.1152* | N-terminal acetyltransferase B complex auxiliary subunit NAA25 |
| *AHE.Chr11.1246* | uncharacterized protein LOC21394409 |
| *AHE.Chr11.1247* | cation/H(+) antiporter 15 |
| *AHE.Chr11.1403* | uncharacterized protein LOC21401872 |
| *AHE.Chr11.1405* | nicotianamine synthase |
| *AHE.Chr11.1743* | Disease resistance protein |
| *AHE.Chr11.1879* | nicalin-1 |
| *AHE.Chr11.1888* | #N/A |
| *AHE.Chr11.1889* | vesicle-associated membrane protein 714-like isoform X3 |
| *AHE.Chr11.2017* | #N/A |
| *AHE.Chr11.2067* | uncharacterized protein LOC21406395 |
| *AHE.Chr11.2369* | sister chromatid cohesion 1 protein 4 |
| *AHE.Chr12.115* | linoleate 13S-lipoxygenase 2-1, chloroplastic |
| *AHE.Chr12.241* | uncharacterized protein LOC21389054 |
| *AHE.Chr12.242* | uncharacterized protein LOC21389053 isoform X1 |
| *AHE.Chr12.302* | uncharacterized protein LOC21398216 |
| *AHE.Chr12.303* | #N/A |
| *AHE.Chr12.304* | receptor-like protein EIX2 |
| *AHE.Chr12.305* | ALBINO3-like protein 2, chloroplastic |
| *AHE.Chr12.306* | Sugar/inositol transporter |
| *AHE.Chr12.307* | Sugar/inositol transporter |
| *AHE.Chr12.308* | protein PELOTA 1 |
| *AHE.Chr12.410* | ethylene-responsive transcription factor 1B |
| *AHE.Chr12.810* | 3-ketoacyl-CoA synthase 12 |
| *AHE.Chr12.970* | uncharacterized protein LOC21408033 |
| *AHE.Chr12.1008* | DExH-box ATP-dependent RNA helicase DExH11 isoform X2 |
| *AHE.Chr12.1288* | phytochrome A |
| *AHE.Chr12.1301* | anaphase-promoting complex subunit 11 isoform X1 |
| *AHE.Chr12.1305* | agamous-like MADS-box protein AGL80 |
| *AHE.Chr12.1306* | uncharacterized protein LOC21405464 |
| *AHE.Chr12.1320* | E3 ubiquitin-protein ligase ZNF598 |
| *AHE.Chr12.1358* | nicotianamine synthase |
| *AHE.Chr12.1359* | homeobox protein knotted-1-like 1 |
| *AHE.Chr12.1426* | lon protease homolog 1, mitochondrial |
| *AHE.Chr12.1427* | hypothetical protein TorRG33x02_293270 |
| *AHE.Chr12.1469_AHE.Chr12.1470* | protein DEHYDRATION-INDUCED 19 homolog 3 |
| *AHE.Chr12.1471* | trichohyalin |
| *AHE.Chr12.1472* | hypothetical protein CISIN_1g003346mg |
| *AHE.Chr12.1714* | #N/A |
| *AHE.Chr12.1715* | E3 ubiquitin-protein ligase HERC2 |
| *AHE.Chr12.1830* | aluminum-activated malate transporter 2 |
| *AHE.Chr12.1837* | uncharacterized protein LOC21392707 |
| *AHE.Chr12.1947* | putative ion channel POLLUX-like 2 isoform X2 |
| *AHE.Chr12.1948* | rRNA-processing protein EFG |
| *AHE.Chr12.1949* | alpha-mannosidase I MNS4 |
| *AHE.Chr12.1952_AHE.Chr12.1953* | uncharacterized protein LOC21392707 |
| *AHE.Chr12.1959* | uncharacterized protein LOC21392706 isoform X2 |
| *AHE.Chr12.2057* | E3 ubiquitin-protein ligase CIP8 |
| *AHE.Chr12.2191* | TMP21-related |
| *AHE.Chr12.2212* | TERMINAL FLOWER1-like protein |
| *AHE.Chr12.2245* | uncharacterized protein At1g08160 |
| *AHE.Chr12.2279* | AP-2 complex subunit alpha-1 |
| *AHE.Chr12.2285* | DDT domain-containing protein PTM |
| *AHE.Chr12.2330* | chromatin assembly factor 1 subunit FAS1 isoform X1 |
| *AHE.Chr13.40* | hypothetical protein F8388_001199 |
| *AHE.Chr13.138* | #N/A |
| *AHE.Chr13.187* | protein NRT1/ PTR FAMILY 3.1 |
| *AHE.Chr13.188* | ACD11 homolog protein |
| *AHE.Chr13.189* | protein PLANT CADMIUM RESISTANCE 2-like |
| *AHE.Chr13.204* | putative receptor-like protein kinase At4g00960 |
| *AHE.Chr13.205* | putative receptor-like protein kinase At4g00960 |
| *AHE.Chr13.215* | uncharacterized protein LOC21395210 |
| *AHE.Chr13.216* | uncharacterized protein LOC21395210 |
| *AHE.Chr13.227* | uncharacterized protein At4g22758 |
| *AHE.Chr13.228* | #N/A |
| *AHE.Chr13.237.6* | UDP-glycosyltransferase TURAN isoform X3 |
| *AHE.Chr13.238* | zinc finger protein VAR3, chloroplastic |
| *AHE.Chr13.494* | exocyst complex component EXO84B isoform X1 |
| *AHE.Chr13.672* | uncharacterized protein LOC112091850 |
| *AHE.Chr13.676* | uric acid degradation bifunctional protein TTL isoform X1 |
| *AHE.Chr13.677* | heavy metal-associated isoprenylated plant protein 4 |
| *AHE.Chr13.678* | Heavy metal-associated domain containing protein |
| *AHE.Chr13.679* | AT-hook motif nuclear-localized protein 23 |
| *AHE.Chr13.683* | protein transport protein SEC23 |
| *AHE.Chr13.685* | pentatricopeptide repeat-containing protein At1g31430 |
| *AHE.Chr13.686* | transcription factor bHLH91 |
| *AHE.Chr13.688* | E3 ubiquitin-protein ligase PUB23 |
| *AHE.Chr13.692* | #N/A |
| *AHE.Chr13.693* | MND1-interacting protein 1 |
| *AHE.Chr13.694* | LOW QUALITY PROTEIN: DAR GTPase 2, mitochondrial |
| *AHE.Chr13.696* | ent-kaurenoic acid oxidase 1 |
| *AHE.Chr13.698* | hypothetical protein L484_023837 |
| *AHE.Chr13.699* | hypothetical protein L484_023837 |
| *AHE.Chr13.703* | Mechanosensitive ion channel protein 10 |
| *AHE.Chr13.705* | protein VASCULAR ASSOCIATED DEATH 1, chloroplastic isoform X2 |
| *AHE.Chr13.706* | uncharacterized protein LOC21406588 |
| *AHE.Chr13.708* | probable glutathione S-transferase |
| *AHE.Chr13.713* | psbQ-like protein 3, chloroplastic |
| *AHE.Chr13.720* | #N/A |
| *AHE.Chr13.726* | xylulose 5-phosphate/phosphate translocator, chloroplastic-like, partial |
| *AHE.Chr13.727* | B3 domain-containing protein Os04g0386900 |
| *AHE.Chr13.728* | PREDICTED: protein NUCLEAR FUSION DEFECTIVE 4-like isoform X2 |
| *AHE.Chr13.729* | uncharacterized protein LOC21406572 isoform X2 |
| *AHE.Chr13.731* | tubulin-folding cofactor A |
| *AHE.Chr13.732* | uncharacterized protein LOC21406570 |
| *AHE.Chr13.760* | transcription factor TCP12 |
| *AHE.Chr13.763* | uncharacterized protein At1g26090, chloroplastic isoform X1 |
| *AHE.Chr13.880* | probable inactive purple acid phosphatase 2 |
| *AHE.Chr13.881* | Putative SNAP25-like protein |
| *AHE.Chr13.882* | putative SNAP25 homologous protein SNAP30 |
| *AHE.Chr13.919* | transcription factor MYB106 |
| *AHE.Chr13.920.3* | anaphase-promoting complex subunit 2 |
| *AHE.Chr13.1087* | protein RICE SALT SENSITIVE 3 |
| *AHE.Chr13.1164* | hypothetical protein G4B88_010328 |
| *AHE.Chr13.1172* | alpha-glucan water dikinase, chloroplastic isoform X1 |
| *AHE.Chr13.1299* | Ran BP2/NZF zinc finger-like superfamily protein |
| *AHE.Chr13.1300* | uncharacterized protein LOC21398585 isoform X1 |
| *AHE.Chr13.1303* | hypothetical protein GH714_029673 |
| *AHE.Chr13.1304* | alpha-ketoglutarate-dependent dioxygenase alkB |
| *AHE.Chr13.1305* | uncharacterized protein LOC120290308 |
| *AHE.Chr13.1409* | uncharacterized protein LOC21400438 |
| *AHE.Chr13.1410* | stress-associated endoplasmic reticulum protein 2-like |
| *AHE.Chr13.1411* | F-box only protein 6 |
| *AHE.Chr13.1473* | RNA polymerase II C-terminal domain phosphatase-like 3 |
| *AHE.Chr14.37* | ethylene-responsive transcription factor ERF113 |
| *AHE.Chr14.225* | vinorine synthase |
| *AHE.Chr14.252* | cleavage and polyadenylation specificity factor subunit CG7185 |
| *AHE.Chr14.253* | actin-related protein 3 |
| *AHE.Chr14.254* | #N/A |
| *AHE.Chr14.290* | cytochrome P450 71B37 |
| *AHE.Chr14.309* | hypothetical protein L484_027645 |
| *AHE.Chr14.310* | protein terminal ear1 |
| *AHE.Chr14.314* | actin-related protein 3 |
| *AHE.Chr14.315* | cleavage and polyadenylation specificity factor subunit CG7185 |
| *AHE.Chr14.322* | LOW QUALITY PROTEIN: ALBINO3-like protein 1, chloroplastic |
| *AHE.Chr14.415_AHE.Chr14.434* | putative beta-1,3-galactosyltransferase 19 |
| *AHE.Chr14.419* | hypothetical protein L484_021054 |
| *AHE.Chr14.516* | cullin-3A |
| *AHE.Chr14.517* | haloacid dehalogenase-like hydrolase domain-containing protein 3 isoform X2 |
| *AHE.Chr14.545* | ribonuclease 1 |
| *AHE.Chr14.574* | FK506-binding protein 5 |
| *AHE.Chr14.575* | uncharacterized protein LOC21398768 |
| *AHE.Chr14.577* | anaphase-promoting complex subunit 2 |
| *AHE.Chr14.686* | serine/threonine-protein kinase par-1 isoform X2 |
| *AHE.Chr14.687.1* | uncharacterized protein LOC21409100 |
| *AHE.Chr14.933* | ATP-dependent DNA helicase homolog RECG, chloroplastic isoform X1 |
| *AHE.Chr14.1085* | pentatricopeptide repeat-containing protein At4g04790, mitochondrial |
| *AHE.Chr14.1238* | nuclear pore complex protein NUP155 |
| *AHE.Chr14.1353* | uncharacterized protein LOC21393737 |
| *AHE.Chr14.1354* | uncharacterized protein LOC21393737 |
| *AHE.Chr14.1358* | NADH dehydrogenase |
| *AHE.Chr14.1390* | transmembrane 9 superfamily member 3 |
| *AHE.Chr14.1402* | phytosulfokine receptor 1 |
| *AHE.Chr14.1405* | Remorin family protein |
| *AHE.Chr14.1413* | ruvB-like protein 1 |
| *AHE.Chr14.1422* | lipase-like PAD4 |
| *AHE.Chr14.1423* | lipase-like PAD4 |
| *AHE.Chr14.1434* | uncharacterized protein LOC21398096 |
| *AHE.Chr14.1506* | protein BASIC PENTACYSTEINE2 |
| *AHE.Chr14.1538* | uncharacterized protein LOC21387328 |
| *AHE.Chr14.1563* | hypothetical protein EUGRSUZ_H04513 |
| *AHE.Chr14.1564* | Heat shock protein 70 family |
| *AHE.Chr15.52* | protein REDUCED WALL ACETYLATION 2 isoform X1 |
| *AHE.Chr15.79* | probable inactive DNA (cytosine-5)-methyltransferase DRM3 |
| *AHE.Chr15.80* | hypothetical protein DVH24_041236 |
| *AHE.Chr15.81* | hypothetical protein |
| *AHE.Chr15.136* | hypothetical protein L484_007750 |
| *AHE.Chr15.223* | hypothetical protein L484_009272 |
| *AHE.Chr15.473* | adenylate isopentenyltransferase 5, chloroplastic |
| *AHE.Chr15.481* | transcription factor MUTE |
| *AHE.Chr15.557* | imidazoleglycerol-phosphate dehydratase |
| *AHE.Chr15.558* | photosystem II D1 precursor processing protein PSB27-H2, chloroplastic isoform X1 |
| *AHE.Chr15.690* | putative deacetoxyvindoline 4-hydroxylase |
| *AHE.Chr15.790.1* | acylamino-acid-releasing enzyme |
| *AHE.Chr15.1175* | small subunit processome component 20 homolog isoform X3 |
| *AHE.Chr15.1342* | #N/A |
| *AHE.Chr15.1354* | uncharacterized protein LOC21394298 |
| *AHE.Chr15.1374* | #N/A |
| *AHE.Chr15.1562* | ferredoxin-thioredoxin reductase, variable chain isoform X1 |
| *AHE.Chr15.1563* | sphingoid long-chain bases kinase 1 |
| *AHE.Chr15.1590* | nuclear pore complex protein NUP160 |
| *AHE.Chr15.1668* | #N/A |
| *AHE.Chr15.1669* | flowering time control protein FPA isoform X1 |
| *AHE.Chr15.1672* | kinesin-like protein KIN-14T |
| *AHE.Chr15.1795* | eIF-2-alpha kinase GCN2 isoform X2 |
| *AHE.Chr15.1797* | uncharacterized protein LOC21407476 |
| *AHE.Chr16.147* | calmodulin-binding transcription activator 5 |
| *AHE.Chr16.148* | PREDICTED: GTP-binding protein SAR1A |
| *AHE.Chr16.344* | chromatin structure-remodeling complex protein SYD isoform X1 |
| *AHE.Chr16.356* | Glutaredoxin-like |
| *AHE.Chr16.357* | acyl-CoA-binding domain-containing protein 6 |
| *AHE.Chr16.358* | hypothetical protein FNV43_RR00803 |
| *AHE.Chr16.1075* | hypothetical protein CUMW_195630 |
| *AHE.Chr16.1376* | transcription factor TCP7 |
| *AHE.Chr16.1380* | sugar transport protein 10 |
| *AHE.Chr16.1381* | thioredoxin X, chloroplastic |
| *AHE.Chr16.1385* | Yif1 family |
| *AHE.Chr16.1407* | uncharacterized protein LOC21407404 |
| *AHE.Chr16.1410* | #N/A |
| *AHE.Chr16.1426* | LOW QUALITY PROTEIN: isoleucine--tRNA ligase, cytoplasmic |
| *AHE.Chr16.1427* | biotin synthase, mitochondrial |
| *AHE.Chr16.1429.1* | U11/U12 small nuclear ribonucleoprotein 35 kDa protein |
| *AHE.Chr16.1430* | hypothetical protein CCACVL1_07147 |
| *AHE.Chr16.1434* | vesicle transport protein GOT1 |
| *AHE.Chr16.1440.1* | uncharacterized protein LOC21399226 isoform X2 |
| *AHE.Chr16.1443* | uncharacterized protein LOC107421838 |
| *AHE.Chr16.1445* | #N/A |
| *AHE.Chr16.1446* | #N/A |
| *AHE.Chr16.1447* | pentatricopeptide repeat-containing protein At2g13600 |
| *AHE.Chr16.1448* | DNA damage-repair/toleration protein DRT100 |
| *AHE.Chr16.1449* | high mobility group B protein 7 |
| *AHE.Chr16.1455* | helicase-like transcription factor CHR28 |
| *AHE.Chr16.1459* | probable protein arginine N-methyltransferase 6 isoform X1 |
| *AHE.Chr16.1485* | histone-lysine N-methyltransferase ASHH3 |
| *AHE.Chr16.1527* | aspartic proteinase-like protein 2 isoform X1 |
| *AHE.Chr16.1528* | uncharacterized protein At1g05835 |
| *AHE.Chr16.1529* | probable protein phosphatase 2C 33 |
| *AHE.Chr16.1555* | protein STRICTOSIDINE SYNTHASE-LIKE 13 |
| *AHE.Chr16.1557* | protein ENHANCED DISEASE RESISTANCE 2-like isoform X2 |
| *AHE.Chr16.1558* | protein ENHANCED DISEASE RESISTANCE 2-like isoform X2 |
| *AHE.Chr16.1567* | PTI1-like tyrosine-protein kinase 1 isoform X2 |
| *AHE.Chr16.1572* | B-box zinc finger protein 25 isoform X1 |
| *AHE.Chr16.1574* | F-box protein SKIP16 |
| *AHE.Chr16.1575* | F-box protein SKIP16 |
| *AHE.Chr16.1596* | #N/A |
| *AHE.Chr16.1604.1* | dihydroorotate dehydrogenase (quinone), mitochondrial |
| *AHE.Chr16.1605* | hypothetical protein G4B88_029362 |
| *AHE.Chr16.1606* | Exocyst complex protein |
| *AHE.Chr16.1608* | myosin-1 |
| *AHE.Chr16.1610* | Riboflavin biosynthesis protein ribF |
| *AHE.Chr16.1613* | LRR domain containing protein |
| *AHE.Chr16.1617* | #N/A |
| *AHE.Chr16.1618* | E3 ubiquitin ligase BIG BROTHER-related |
| *AHE.Chr17.28* | probable envelope ADP,ATP carrier protein, chloroplastic |
| *AHE.Chr17.30* | protein root UVB sensitive 5 isoform X2 |
| *AHE.Chr17.31* | protein root UVB sensitive 5 isoform X2 |
| *AHE.Chr17.32* | dehydrodolichyl diphosphate synthase 2 |
| *AHE.Chr17.33* | #N/A |
| *AHE.Chr17.34* | high mobility group B protein 1 |
| *AHE.Chr17.38* | chlorophyll a-b binding protein CP29.2, chloroplastic |
| *AHE.Chr17.45* | fructose-1,6-bisphosphatase, chloroplastic |
| *AHE.Chr17.47* | hypothetical protein L484_023633 |
| *AHE.Chr17.48* | hypothetical protein L484_023633 |
| *AHE.Chr17.52* | hypothetical protein L484_023631 |
| *AHE.Chr17.56* | protein SSUH2 homolog |
| *AHE.Chr17.57* | hypothetical protein L484_023604 |
| *AHE.Chr17.61* | UPF0503 protein At3g09070, chloroplastic |
| *AHE.Chr17.66* | phosphatidylinositol:ceramide inositolphosphotransferase 1 |
| *AHE.Chr17.76* | reticulon-like protein B12 |
| *AHE.Chr17.77* | E3 ubiquitin-protein ligase ZNF645 |
| *AHE.Chr17.82* | mRNA-capping enzyme isoform X2 |
| *AHE.Chr17.87* | uncharacterized protein LOC21392790 |
| *AHE.Chr17.102* | transcription factor DIVARICATA |
| *AHE.Chr17.108* | BAHD acyltransferase DCR |
| *AHE.Chr17.111* | sulfoquinovosyl transferase SQD2 |
| *AHE.Chr17.115* | Serine/threonine-protein kinase ATG1 |
| *AHE.Chr17.132* | Auxin influx transporter |
| *AHE.Chr17.141_AHE.Chr17.143* | LOW QUALITY PROTEIN: lysine-specific demethylase JMJ25 |
| *AHE.Chr17.142* | #N/A |
| *AHE.Chr17.157* | #N/A |
| *AHE.Chr17.166* | Replication factor A protein |
| *AHE.Chr17.167* | putative ribonuclease |
| *AHE.Chr17.210* | importin subunit beta-1 |
| *AHE.Chr17.257.1* | uncharacterized protein LOC21399448 isoform X1 |
| *AHE.Chr17.343* | TOM1-like protein 6 |
| *AHE.Chr17.372.2* | AMP deaminase isoform X1 |
| *AHE.Chr17.480* | probable serine/threonine-protein kinase PBL1 isoform X3 |
| *AHE.Chr17.482.1* | B3 domain-containing transcription repressor VAL2 isoform X1 |
| *AHE.Chr17.684* | trafficking protein particle complex subunit 11 |
| *AHE.Chr17.984* | probable disease resistance protein At4g19060 |
| *AHE.Chr17.985* | hypothetical protein L484_024056 |
| *AHE.Chr17.1305* | homeobox protein BEL1 homolog |
| *AHE.Chr17.1335* | uncharacterized protein LOC21406988 |
| *AHE.Chr17.1422* | F-box/LRR-repeat protein |
| *AHE.Chr17.1458* | phospholipase D beta 1 |
| *AHE.Chr17.1469* | anthocyanin synthase |
| *AHE.Chr17.1562* | protein YABBY 4 |
| *AHE.Chr17.1630* | (S)-N-methylcoclaurine 3'-hydroxylase isozyme 1 |
| *AHE.Chr17.1631* | (S)-N-methylcoclaurine 3'-hydroxylase isozyme 1 |
| *AHE.Chr17.1635* | scarecrow-like protein 28 |
| *AHE.Chr17.1636* | #N/A |
| *AHE.Chr17.1637* | scarecrow-like protein 28 |
| *AHE.Chr17.1638* | plant UBX domain-containing protein 8 |
| *AHE.Chr18.74* | protein arginine N-methyltransferase 7 isoform X2 |
| *AHE.Chr18.75* | LOW QUALITY PROTEIN: probable cinnamyl alcohol dehydrogenase 1 |
| *AHE.Chr18.84* | asparagine--tRNA ligase, chloroplastic/mitochondrial isoform X1 |
| *AHE.Chr18.85* | protein transport protein SEC16A homolog |
| *AHE.Chr18.122* | hypothetical protein L484_023188 |
| *AHE.Chr18.195* | LOW QUALITY PROTEIN: rRNA biogenesis protein rrp36 |
| *AHE.Chr18.225* | uncharacterized protein LOC21393848 isoform X2 |
| *AHE.Chr18.274* | AT-hook motif nuclear-localized protein 5 |
| *AHE.Chr18.505* | dynamin-related protein 5A |
| *AHE.Chr18.535* | bidirectional sugar transporter SWEET4 |
| *AHE.Chr18.536* | protein KINESIN LIGHT CHAIN-RELATED 1 |
| *AHE.Chr18.538* | hypothetical protein L484_007825 |
| *AHE.Chr18.616* | pyruvate kinase isozyme G, chloroplastic |
| *AHE.Chr18.617* | Nitrate transporter 1.5 |
| *AHE.Chr18.642* | bidirectional sugar transporter SWEET4 |
| *AHE.Chr18.643* | protein KINESIN LIGHT CHAIN-RELATED 1 |
| *AHE.Chr18.648* | importin subunit beta-1 |
| *AHE.Chr18.702* | transcription factor MYC2 |
| *AHE.Chr18.883* | methanol O-anthraniloyltransferase |
| *AHE.Chr18.898* | LOW QUALITY PROTEIN: callose synthase 5 |
| *AHE.Chr18.930* | myb family transcription factor PHL11 |
| *AHE.Chr18.1012* | uncharacterized protein At4g29660 |
| *AHE.Chr18.1024* | uncharacterized protein LOC21405551 |
| *AHE.Chr18.1025* | uncharacterized protein LOC21405551 |
| *AHE.Chr18.1245* | importin subunit beta-1 |
| *AHE.Chr18.1315* | delta-1-pyrroline-5-carboxylate synthase |
| *AHE.Chr18.1361* | probable WRKY transcription factor 49 isoform X2 |
| *AHE.Chr19.1* | uncharacterized protein LOC21387445 |
| *AHE.Chr19.2* | ethylene-responsive transcription factor ERF015 |
| *AHE.Chr19.3* | LOW QUALITY PROTEIN: pentatricopeptide repeat-containing protein At4g31070, mitochondrial |
| *AHE.Chr19.4* | -ketoacyl-CoA synthase 15 |
| *AHE.Chr19.5* | uncharacterized protein LOC112091371 |
| *AHE.Chr19.27* | uncharacterized protein LOC21391599, partial |
| *AHE.Chr19.28* | methyl-CpG-binding domain-containing protein 2 |
| *AHE.Chr19.31* | ethanolamine-phosphate cytidylyltransferase |
| *AHE.Chr19.32* | uncharacterized protein LOC21391605 |
| *AHE.Chr19.33* | isoamylase 1, chloroplastic isoform X1 |
| *AHE.Chr19.226* | Small auxin-up RNA |
| *AHE.Chr19.235* | transcription factor bHLH137 |
| *AHE.Chr19.256* | bidirectional sugar transporter N3 |
| *AHE.Chr19.400* | callose synthase 10 isoform X2 |
| *AHE.Chr19.455* | cytochrome P450 71A24-like |
| *AHE.Chr19.513* | LOB domain-containing protein 25 |
| *AHE.Chr19.518* | uncharacterized protein LOC21397693 |
| *AHE.Chr19.607* | RNA-dependent RNA polymerase 2 |
| *AHE.Chr19.608* | V-type proton ATPase subunit F-like |
| *AHE.Chr19.619* | U-box domain-containing protein 19 |
| *AHE.Chr19.709* | protein ALP1-like |
| *AHE.Chr19.710* | peptidyl-prolyl cis-trans isomerase CYP57 |
| *AHE.Chr19.749* | formin-like protein 4 |
| *AHE.Chr19.750* | formin-like protein 4 |
| *AHE.Chr19.788* | Momilactone A synthase |
| *AHE.Chr19.996* | uncharacterized protein LOC21398582 isoform X1 |
| *AHE.Chr19.1020* | putative UDP-glucuronate:xylan alpha-glucuronosyltransferase 5 |
| *AHE.Chr19.1093* | probable helicase CHR10 |
| *AHE.Chr19.1300.1* | Guanine nucleotide-binding protein, beta subunit |
| *AHE.Chr19.1312* | inactive poly |
| *AHE.Chr19.1346* | 30-kDa cleavage and polyadenylation specificity factor 30 isoform X1 |
| *AHE.Chr19.1347* | #N/A |
| *AHE.Chr19.1348* | hypothetical protein PRUPE_1G160400 |
| *AHE.Chr19.1356* | probable protein phosphatase 2C 8 isoform X1 |
| *AHE.Chr19.1366* | Dolichyl-diphosphooligosaccharide--protein glycosyltransferase subunit STT3 |
| *AHE.Chr19.1368* | LOW QUALITY PROTEIN: kinase-interacting family protein |
| *AHE.Chr19.1393* | sucrose synthase 6 |
| *AHE.Chr20.9* | prefoldin subunit 1 |
| *AHE.Chr20.280* | agamous-like MADS-box protein AGL1 |
| *AHE.Chr20.368_AHE.Chr20.379* | stilbene synthase 8 |
| *AHE.Chr20.539* | serine/threonine protein phosphatase 2A 55 kDa regulatory subunit B beta isoform isoform X2 |
| *AHE.Chr20.540* | protein MICRORCHIDIA 6 isoform X1 |
| *AHE.Chr20.590* | CASP-like protein 4A3 |
| *AHE.Chr20.605* | LOW QUALITY PROTEIN: serine/threonine-protein kinase ATM, partial |
| *AHE.Chr20.673* | hypothetical protein L484_008932 |
| *AHE.Chr20.674* | serine/threonine-protein kinase AtPK2/AtPK19 |
| *AHE.Chr20.675* | proline-, glutamic acid- and leucine-rich protein 1 |
| *AHE.Chr20.694* | uncharacterized protein LOC109009495 |
| *AHE.Chr20.695* | probable glycosyltransferase At3g07620 |
| *AHE.Chr20.864* | mini zinc finger protein 3 |
| *AHE.Chr20.1172* | protein FLX-like 1 isoform X1 |
| *AHE.Chr20.1196* | uncharacterized protein At3g17950 |
| *AHE.Chr20.1233* | Exonuclease/helicase-like |
| *AHE.Chr20.1244* | LOW QUALITY PROTEIN: probable cyclic nucleotide-gated ion channel 20, chloroplastic |
| *AHE.Chr20.1245* | #N/A |
| *AHE.Chr20.1248* | #N/A |
| *AHE.Chr20.1252* | Transcription factor |
| *AHE.Chr20.1254* | zinc finger protein BRUTUS |
| *AHE.Chr20.1413* | zinc finger protein BRUTUS |
| *AHE.Chr20.1454* | uncharacterized PKHD-type hydroxylase At1g22950 |
| *AHE.Chr20.1471* | Prostaglandin G/H synthase 1 |
| *AHE.Chr21.14* | uncharacterized protein At2g33490 isoform X2 |
| *AHE.Chr21.15* | uncharacterized protein LOC21389133 isoform X1 |
| *AHE.Chr21.111* | glucose-6-phosphate 1-dehydrogenase, cytoplasmic |
| *AHE.Chr21.112* | plant UBX domain-containing protein 1 isoform X1 |
| *AHE.Chr21.313* | inositol hexakisphosphate and diphosphoinositol-pentakisphosphate kinase VIP2 isoform X2 |
| *AHE.Chr21.426* | hypothetical protein G4B88_015654 |
| *AHE.Chr21.622* | protein GLUTAMINE DUMPER 2 |
| *AHE.Chr21.623* | #N/A |
| *AHE.Chr21.722* | LRR receptor-like serine/threonine-protein kinase RPK2 |
| *AHE.Chr21.812* | casparian strip membrane protein 1 |
| *AHE.Chr21.866* | probable carboxylesterase 17 |
| *AHE.Chr21.955* | hypothetical protein L484_022219 |
| *AHE.Chr21.1077* | histone-lysine N-methyltransferase ATXR3 isoform X1 |
| *AHE.Chr21.1125* | G-type lectin S-receptor-like serine/threonine-protein kinase At2g19130 |
| *AHE.Chr21.1214* | serine/threonine-protein kinase ATR |
| *AHE.Chr21.1385* | hypothetical protein F0562_008542 |
| *AHE.Chr21.1386* | hypothetical protein L484_026291 |
| *AHE.Chr21.1463* | DNA-directed RNA polymerase I subunit 2 |
| *AHE.Chr21.1548* | transcription factor JUNGBRUNNEN 1 |
| *AHE.Chr21.1566* | putative ATP-dependent RNA helicase YTHDC2 |
| *AHE.Chr21.1567* | DExH-box ATP-dependent RNA helicase DExH6 |
| *AHE.Chr21.1568* | putative ATP-dependent RNA helicase YTHDC |
| *AHE.Chr21.1569* | DExH-box ATP-dependent RNA helicase DExH6 |
| *AHE.Chr21.1590* | AT-hook motif nuclear-localized protein 16 |
| *AHE.Chr21.1592* | uncharacterized protein LOC21404244 isoform X1 |
| *AHE.Chr21.1607* | callose synthase 7 |
| *AHE.Chr21.1617* | persulfide dioxygenase ETHE1 homolog, mitochondrial isoform X1 |
| *AHE.Chr21.1636* | hypothetical protein TorRG33x02_307100 |
| *AHE.Chr21.1637* | uncharacterized protein LOC21400713 |
| *AHE.Chr21.1714* | uncharacterized protein At4g15545 |
| *AHE.Chr21.1769* | hypothetical protein Csa_020731 |
| *AHE.Chr21.1770* | plastid division protein CDP1, chloroplastic |
| *AHE.Chr21.1773* | LOW QUALITY PROTEIN: DNA-binding protein RHL1 |
| *AHE.Chr21.1811* | LOW QUALITY PROTEIN: probable cyclic nucleotide-gated ion channel 20, chloroplastic |
| *AHE.Chr21.1973* | alpha-1,6-mannosyl-glycoprotein 2-beta-N-acetylglucosaminyltransferase |
| *AHE.Chr21.2055* | GBF-interacting protein 1-like isoform X2 |
| *AHE.Chr21.2057* | heterogeneous nuclear ribonucleoprotein 1 |
| *AHE.Chr21.2058* | #N/A |
| *AHE.Chr21.2063* | ribulose-1,5 bisphosphate carboxylase/oxygenase large subunit N-methyltransferase, chloroplastic |
| *AHE.Chr21.2141* | Myo-inositol-1-phosphate synthase |
| *AHE.Chr21.2177* | protease Do-like 7 isoform X2 |
| *AHE.Chr21.2219* | transmembrane protein 256 homolog |
| *AHE.Chr21.2221* | uncharacterized protein LOC21395421 |
| *AHE.Chr21.2222* | #N/A |
| *AHE.Chr21.2225* | fimbrin-5 |
| *AHE.Chr21.2227* | #N/A |
| *AHE.Chr21.2228* | hypothetical protein L484_004993 |
| *AHE.Chr21.2229* | #N/A |
| *AHE.Chr21.2230* | #N/A |
| *AHE.Chr22.17* | chalcone synthase |
| *AHE.Chr22.152* | sulfite oxidase |
| *AHE.Chr22.153* | cytochrome P450 94B3 |
| *AHE.Chr22.154* | Fidgetin-like protein 1 |
| *AHE.Chr22.155* | SWI/SNF complex component SNF12 homolog |
| *AHE.Chr22.192* | hypothetical protein E3N88_14086 |
| *AHE.Chr22.193* | phosducin-like protein 3 |
| *AHE.Chr22.194* | COP9 signalosome complex subunit 3 |
| *AHE.Chr22.307* | nuclear pore complex protein GP210 |
| *AHE.Chr22.522* | hypothetical protein FNV43_RR14452 |
| *AHE.Chr22.551* | SUPPRESSOR OF ABI3-5 isoform X1 |
| *AHE.Chr22.786* | hypothetical protein TorRG33x02_342640 |
| *AHE.Chr22.802* | transcription factor TCP13 |
| *AHE.Chr22.878* | uncharacterized protein LOC112094014 |
| *AHE.Chr22.879* | tRNA threonylcarbamoyladenosine dehydratase |
| *AHE.Chr22.1004* | auxin transport protein BIG |
| *AHE.Chr22.1019* | DNA-directed RNA polymerase 2B, chloroplastic/mitochondrial |
| *AHE.Chr22.1020* | LOW QUALITY PROTEIN: kanadaptin |
| *AHE.Chr22.1076* | delta(7)-sterol-C5(6)-desaturase, partial |
| *AHE.Chr22.1134* | Transducin beta-like protein 2 |
| *AHE.Chr22.1519* | hypothetical protein L484_011934 |
| *AHE.Chr22.1762* | plant UBX domain-containing protein 4 |
| *AHE.Chr22.1763* | #N/A |
| *AHE.Chr22.1786* | hypothetical protein FH972_014338 |
| *AHE.Chr22.1787* | condensin complex subunit 2 isoform X1 |
| *AHE.Chr22.1848* | callose synthase 12 |
| *AHE.Chr22.1861* | #N/A |
| *AHE.Chr22.1912* | callose synthase 7 |
| *AHE.Chr22.2067* | protein PAT1 homolog 2 |
| *AHE.Chr22.2120* | putative clathrin assembly protein At4g40080 |
| *AHE.Chr22.2123.1* | uncharacterized protein LOC21398538 isoform X1 |
| *AHE.Chr22.2153* | probable WRKY transcription factor 69 |
| *AHE.Chr22.2154* | ras-related protein Rab11D |
| *AHE.Chr22.2155* | hypothetical protein L484_026291 |
| *AHE.Chr22.2161* | probable alpha,alpha-trehalose-phosphate synthase |
| *AHE.Chr22.2162* | G-type lectin S-receptor-like serine/threonine-protein kinase SD2-5 |
| *AHE.Chr22.2164* | hypothetical protein L484_021362 |
| *AHE.Chr22.2165* | ATP-dependent zinc metalloprotease FTSH 2, chloroplastic |
| *AHE.Chr22.2177* | probable sugar phosphate/phosphate translocator At1g06470 |
| *AHE.Chr22.2178* | heat stress transcription factor A-4c |
| *AHE.Chr22.2183* | probable protein S-acyltransferase 19 |
| *AHE.Chr22.2184* | probable protein S-acyltransferase 19 |
| *AHE.Chr22.2279* | hypothetical protein G4B88_007217 |
| *AHE.Chr22.2317* | LEAF RUST 10 DISEASE-RESISTANCE LOCUS RECEPTOR-LIKE PROTEIN KINASE-like 2.5 |
| *AHE.Chr23.42* | peroxisomal membrane protein 13 |
| *AHE.Chr23.43* | hypothetical protein L484_018107 |
| *AHE.Chr23.126* | 2-alkenal reductase (NADP(+)-dependent) |
| *AHE.Chr23.289* | umecyanin |
| *AHE.Chr23.290* | Transcription initiation factor |
| *AHE.Chr23.301* | hypothetical protein L484_013645 |
| *AHE.Chr23.302* | U4/U6.U5 tri-snRNP-associated protein 2 |
| *AHE.Chr23.487* | probable GTP diphosphokinase RSH2, chloroplastic |
| *AHE.Chr23.488* | transcription initiation factor TFIID subunit 9-like |
| *AHE.Chr23.494* | LOW QUALITY PROTEIN: exportin-T |
| *AHE.Chr23.513* | transcription factor WER |
| *AHE.Chr23.524.2* | uncharacterized protein LOC21386182 isoform X1 |
| *AHE.Chr23.525* | developmentally-regulated G-protein 2 isoform X1 |
| *AHE.Chr23.526* | putative pentatricopeptide repeat-containing protein At5g37570 isoform X1 |
| *AHE.Chr23.527* | Carbon-nitrogen hydrolase |
| *AHE.Chr23.541* | transmembrane 9 superfamily member 7 |
| *AHE.Chr23.542* | #N/A |
| *AHE.Chr23.543* | uncharacterized protein LOC21388031 |
| *AHE.Chr23.544* | beta-galactosidase 1 |
| *AHE.Chr23.621* | nuclear poly(A) polymerase 4 isoform X2 |
| *AHE.Chr23.622* | hexokinase-1 |
| *AHE.Chr23.644* | nuclear poly(A) polymerase 4 isoform X2 |
| *AHE.Chr23.705* | hypothetical protein CDL15_Pgr014964 |
| *AHE.Chr23.706* | golgin candidate 5 |
| *AHE.Chr23.707* | protein tesmin/TSO1-like CXC 6 isoform X2 |
| *AHE.Chr23.726* | ATP-dependent DNA helicase DDX11 |
| *AHE.Chr23.791* | pleiotropic drug resistance protein 1 |
| *AHE.Chr23.824* | MAPK kinase substrate protein At1g80180 |
| *AHE.Chr23.956* | Poly(ADP-ribose) polymerase, catalytic domain containing protein |
| *AHE.Chr23.957* | protein HEAT-STRESS-ASSOCIATED 32-like |
| *AHE.Chr23.958* | uncharacterized protein LOC21397963 |
| *AHE.Chr23.1436* | RNA polymerase-associated protein Rtf |
| *AHE.Chr23.1599* | Parvalbumin |
| *AHE.Chr23.1762* | cationic amino acid transporter 1 |
| *AHE.Chr23.2001* | peroxidase A2 |
| *AHE.Chr23.2036* | protein CHROMATIN REMODELING 4 isoform X1 |
| *AHE.Chr24.22* | polygalacturonase |
| *AHE.Chr24.26* | polygalacturonase |
| *AHE.Chr24.27* | ADH_zinc_N domain-containing protein |
| *AHE.Chr24.28* | 2-alkenal reductase (NADP(+)-dependent) |
| *AHE.Chr24.30* | Dual specificity protein kinase shkC |
| *AHE.Chr24.31* | mitochondrial proton/calcium exchanger protein |
| *AHE.Chr24.37* | BTB/POZ and TAZ domain-containing protein 3 |
| *AHE.Chr24.38* | acyl-coenzyme A thioesterase 9, mitochondrial |
| *AHE.Chr24.42* | uncharacterized protein LOC21403279 isoform X1 |
| *AHE.Chr24.43* | #N/A |
| *AHE.Chr24.44* | 60S ribosomal protein L5 |
| *AHE.Chr24.112* | sacsin |
| *AHE.Chr24.300* | tRNA (guanine(9)-N1)-methyltransferase |
| *AHE.Chr24.301* | PREDICTED: nuclear transcription factor Y subunit B-6 |
| *AHE.Chr24.302* | trihelix transcription factor GT-2 |
| *AHE.Chr24.329* | P-type ATPase |
| *AHE.Chr24.483* | Nuclear pore protein |
| *AHE.Chr24.518* | uncharacterized protein LOC21392906 |
| *AHE.Chr24.519* | #N/A |
| *AHE.Chr24.520* | disease resistance protein RPP8 |
| *AHE.Chr24.551* | myb-related protein Zm38 |
| *AHE.Chr24.552* | transcription initiation factor TFIID subunit 12b |
| *AHE.Chr24.726* | lysM domain receptor-like kinase 3 |
| *AHE.Chr24.727* | hypothetical protein EUGRSUZ_J02936 |
| *AHE.Chr24.857* | DNA-repair protein XRCC1 |
| *AHE.Chr24.858* | N-terminal acetyltransferase A complex auxiliary subunit NAA15 isoform X1 |
| *AHE.Chr24.859* | E3 ubiquitin-protein ligase At1g63170 |
| *AHE.Chr24.1353* | #N/A |
| *AHE.Chr24.1382* | transcription factor IIIB 60 kDa subunit |
| *AHE.Chr24.1537* | protein LIGHT-DEPENDENT SHORT HYPOCOTYLS 10 |
| *AHE.Chr24.1619* | uncharacterized protein LOC21387046 |
| *AHE.Chr24.1833* | uncharacterized protein LOC21394693 |
| *AHE.Chr24.1887* | DNA damage-binding protein 1 isoform X2 |
| *AHE.Chr24.2005* | nucleolar MIF4G domain-containing protein 1-like, partial |
| *AHE.Chr24.2088* | MADS-box protein FBP24 |
| *AHE.Chr24.2091* | DNA-directed RNA polymerase 2B |
| *AHE.Chr24.2098* | chaperonin 60 subunit alpha 2, chloroplastic isoform X1 |
| *AHE.Chr24.2104* | ankyrin repeat-containing protein NPR4 |
| *AHE.Chr24.2109* | protein CHROMATIN REMODELING 4 isoform X1 |
| *AHE.Chr24.2113* | hypothetical protein CDL15_Pgr004606 |
| *AHE.Chr24.2127* | ZIP1 protein |
| *AHE.Chr25.21* | #N/A |
| *AHE.Chr25.22* | #N/A |
| *AHE.Chr25.43* | vitellogenin-2 |
| *AHE.Chr25.44* | glucan endo-1,3-beta-glucosidase 8 |
| *AHE.Chr25.45* | #N/A |
| *AHE.Chr25.239* | serine/threonine-protein kinase PCRK1 |
| *AHE.Chr25.241* | hypothetical protein L484_024359 |
| *AHE.Chr25.242* | RNA polymerase sigma factor sigF, chloroplastic |
| *AHE.Chr25.245* | uncharacterized protein LOC21407099 |
| *AHE.Chr25.348* | Carotene epsilon-monooxygenase |
| *AHE.Chr25.461* | Werner Syndrome-like exonuclease |
| *AHE.Chr25.530* | CAAX prenyl protease 2 |
| *AHE.Chr25.533* | ubiquitin-conjugating enzyme E2 22-like |
| *AHE.Chr25.692* | uncharacterized protein LOC21399947 |
| *AHE.Chr25.708* | cytochrome P450 94C1 |
| *AHE.Chr25.761* | protein EMBRYO SAC DEVELOPMENT ARREST 30 |
| *AHE.Chr25.782* | protein FAR1-RELATED SEQUENCE 7-like |
| *AHE.Chr25.785* | hypothetical protein L484_002648 |
| *AHE.Chr25.786* | bZIP transcription factor 17 |
| *AHE.Chr25.787* | uncharacterized protein LOC21386213 |
| *AHE.Chr25.791* | sugar transport protein MST4 |
| *AHE.Chr25.793* | chloride channel protein CLC-d isoform X1 |
| *AHE.Chr25.794* | long chain acyl-CoA synthetase 6, peroxisomal |
| *AHE.Chr25.819* | calmodulin-binding protein 25 |
| *AHE.Chr25.824* | exportin-7 |
| *AHE.Chr25.832* | regulation of nuclear pre-mRNA domain-containing protein 1B |
| *AHE.Chr25.843* | transcription elongation factor SPT6 homolog isoform X1 |
| *AHE.Chr25.853* | exocyst complex component SEC15A |
| *AHE.Chr25.854* | #N/A |
| *AHE.Chr25.859* | ubiquitin carboxyl-terminal hydrolase 5 isoform X1 |
| *AHE.Chr25.860* | Thioredoxin |
| *AHE.Chr25.862* | histone-lysine N-methyltransferase SUVR3 isoform X1 |
| *AHE.Chr25.880* | pentatricopeptide repeat-containing protein At1g80270, mitochondrial |
| *AHE.Chr25.888.1* | DNA gyrase, subunit B |
| *AHE.Chr25.907* | uncharacterized protein LOC112094718 |
| *AHE.Chr25.1247* | uncharacterized protein LOC21388445 isoform X1 |
| *AHE.Chr25.1353* | #N/A |
| *AHE.Chr25.1394* | uncharacterized protein LOC21388445 isoform X1 |
| *AHE.Chr26.27* | 60S ribosomal protein L7-2 |
| *AHE.Chr26.28* | protein ACTIVITY OF BC1 COMPLEX KINASE 3, chloroplastic |
| *AHE.Chr26.30* | vitellogenin-2 |
| *AHE.Chr26.31* | glucan endo-1,3-beta-glucosidase 8 |
| *AHE.Chr26.75* | #N/A |
| *AHE.Chr26.76* | NB-ARC domain, LRR domain containing protein |
| *AHE.Chr26.294* | splicing factor 3A subunit 2 |
| *AHE.Chr26.295* | phosphatidylinositol 4-phosphate 5-kinase 9 isoform X3 |
| *AHE.Chr26.422* | TERMINAL FLOWER1-like protein |
| *AHE.Chr26.479* | protein SMAX1-LIKE 3 |
| *AHE.Chr26.559* | ATP-binding cassette containing protein |
| *AHE.Chr26.560* | 14kDa zinc-binding protein |
| *AHE.Chr26.561* | ABC transporter B family member 29 |
| *AHE.Chr26.562* | heterogeneous nuclear ribonucleoprotein Q isoform X2 |
| *AHE.Chr26.563* | F-box/kelch-repeat protein At1g57790 |
| *AHE.Chr26.595* | TERMINAL FLOWER1-like protein |
| *AHE.Chr26.836* | polygalacturonase inhibitor |
| *AHE.Chr26.837* | PREDICTED: polygalacturonase inhibitor-like |
| *AHE.Chr26.838* | polygalacturonase inhibitor |
| *AHE.Chr26.871* | #N/A |
| *AHE.Chr26.872* | uncharacterized protein LOC21410629 |
| *AHE.Chr26.1060* | ferredoxin-dependent glutamate synthase, chloroplastic |
| *AHE.Chr26.1360* | protein EMBRYO SAC DEVELOPMENT ARREST 30 |
| *AHE.Chr26.1393* | glutathione S-transferase zeta class isoform X1 |
| *AHE.Chr26.1445* | 4-alpha-glucanotransferase DPE2 |
| *AHE.Chr26.1482* | putative SWI/SNF-related matrix-associated actin-dependent regulator of chromatin subfamily A member 3-like 1 |
| *AHE.Chr26.1561.1* | ribosomal lysine N-methyltransferase 3 |
| *AHE.Chr26.1568* | cleavage stimulation factor subunit 77 |
| *AHE.Chr26.1570* | phosphatidylinositol transfer protein 3 |
| *AHE.Chr26.1598* | probable apyrase 7 |
| *AHE.Chr26.1599* | conserved oligomeric Golgi complex subunit 6 |
| *AHE.Chr27.254* | TATA-binding protein-associated factor BTAF1 isoform X2 |
| *AHE.Chr27.255* | TATA-binding protein-associated factor BTAF1 isoform X2 |
| *AHE.Chr27.256* | TATA-binding protein-associated factor BTAF1 isoform X2 |
| *AHE.Chr27.389* | RNA-binding protein 1 |
| *AHE.Chr27.450* | probable 6-phosphogluconolactonase 4, chloroplastic |
| *AHE.Chr27.451* | GTPase-activating protein gyp7 isoform X1 |
| *AHE.Chr27.454* | ATPase family AAA domain-containing protein 1 |
| *AHE.Chr27.455* | ATPase family AAA domain-containing protein 1 |
| *AHE.Chr27.542* | probable isoaspartyl peptidase/L-asparaginase 3 isoform X1 |
| *AHE.Chr27.543* | LOW QUALITY PROTEIN: ceramide kinase |
| *AHE.Chr27.795* | aquaporin PIP1-1 |
| *AHE.Chr27.816* | WUSCHEL-related homeobox 5 |
| *AHE.Chr27.826* | #N/A |
| *AHE.Chr27.827* | C2 domain containing protein |
| *AHE.Chr27.828* | #N/A |
| *AHE.Chr27.844* | mRNA-capping enzyme isoform X2 |
| *AHE.Chr27.858* | proline transporter 2 |
| *AHE.Chr27.879* | hypothetical protein L484_010382 |
| *AHE.Chr27.924* | probable transcriptional regulator RABBIT EARS |
| *AHE.Chr27.1074* | U1 small nuclear ribonucleoprotein C |
| *AHE.Chr27.1106* | chalcone isomerase |
| *AHE.Chr27.1158* | protein NRT1/ PTR FAMILY 2.5 |
| *AHE.Chr27.1286* | thioredoxin-like 3-2, chloroplastic isoform X1 |
| *AHE.Chr27.1366* | putative serine/threonine-protein kinase |
| *AHE.Chr27.1530* | zinc finger CCCH domain-containing protein 37 isoform X1 |
| *AHE.Chr27.1542* | flowering time control protein FY isoform X2 |
| *AHE.Chr27.1548* | translation initiation factor eIF-2B subunit beta |
| *AHE.Chr27.1556* | hypothetical protein L484_012581 |
| *AHE.Chr27.1557* | nuclear pore complex protein NUP1 |
| *AHE.Chr27.1572.2* | ACT domain-containing protein ACR12 |
| *AHE.Chr27.1574* | #N/A |
| *AHE.Chr27.1576* | hypothetical protein L484_020255 |
| *AHE.Chr27.1577* | #N/A |
| *AHE.Chr27.1578* | basic leucine zipper 6 |
| *AHE.Chr27.1581* | Apoptosis-inducing factor-A-like protein |
| *AHE.Chr27.1583* | uncharacterized protein LOC21401330 |
| *AHE.Chr27.1584* | L-aminoadipate-semialdehyde dehydrogenase-phosphopantetheinyl transferase |
| *AHE.Chr27.1586.1* | uncharacterized protein LOC21403166 isoform X2 |
| *AHE.Chr27.1588* | ethylene-responsive transcription factor WRI1 |
| *AHE.Chr27.1589* | F-box protein GID2 |
| *AHE.Chr27.1607* | membrin-11 |
| *AHE.Chr27.1623* | ribonuclease H2 subunit A |
| *AHE.Chr27.1639* | hypothetical protein L484_023996 |
| *AHE.Chr27.1701* | dnaJ protein homolog |
| *AHE.Chr27.1729* | protein ELC-like |
| *AHE.Chr27.1730.1* | uncharacterized protein LOC21406759 |
| *AHE.Chr27.1735* | #N/A |
| *AHE.Chr27.1742* | protein NDH-DEPENDENT CYCLIC ELECTRON FLOW 5 |
| *AHE.Chr27.1782* | NAC domain-containing protein 86 |
| *AHE.Chr27.1785* | myb-related protein 308 |
| *AHE.Chr27.1838* | neutral/alkaline invertase 3, chloroplastic |
| *AHE.Chr27.1855* | MTP9 protein |
| *AHE.Chr27.1857* | ETHYLENE INSENSITIVE 3-like 1 protein |
| *AHE.Chr28.6* | uncharacterized protein LOC21402597 |
| *AHE.Chr28.7* | WEB family protein At5g16730, chloroplastic isoform X2 |
| *AHE.Chr28.79* | ERAD-associated E3 ubiquitin-protein ligase HRD1B isoform X2 |
| *AHE.Chr28.89* | uncharacterized protein LOC21402633 |
| *AHE.Chr28.90* | serine/threonine-protein phosphatase 4 regulatory subunit 2 |
| *AHE.Chr28.165* | AAA-ATPase At3g50940 |
| *AHE.Chr28.238* | U-box domain-containing protein 43 |
| *AHE.Chr28.246* | uncharacterized protein LOC21395087 |
| *AHE.Chr28.248* | dof zinc finger protein DOF4.6 |
| *AHE.Chr28.327* | CLP protease regulatory subunit CLPX1, mitochondrial |
| *AHE.Chr28.513* | hypothetical protein G4B88_019432 |
| *AHE.Chr28.558* | K(+) efflux antiporter 5 |
| *AHE.Chr28.785* | TIR-NBS-LRR-like protein |
| *AHE.Chr28.908* | ATP-dependent Clp protease proteolytic subunit 3, chloroplastic |
| *AHE.Chr28.909.2* | RNA binding protein |
| *AHE.Chr28.978* | Amino acid transporter, transmembrane domain containing protein |
| *AHE.Chr28.984* | transcriptional activator DEMETER |
| *AHE.Chr28.1106* | uncharacterized protein LOC21408126 |
| *AHE.Chr28.1260* | endoglucanase 17 |
| *AHE.Chr28.1261* | Pectinesterase inhibitor domain containing protein |
| *AHE.Chr28.1361* | potassium transporter 5 isoform X1 |
| *AHE.Chr28.1365* | potassium transporter 5 isoform X1 |
| *AHE.Chr28.1370* | #N/A |
| *AHE.Chr28.1476* | metal tolerance protein 11 |
| *AHE.Chr28.1478* | beta-hexosaminidase 1 |
| *AHE.Chr28.1513* | zinc finger protein ZAT11 |
| *AHE.Chr28.1774* | LOW QUALITY PROTEIN: uncharacterized protein LOC21399817 |
| *AHE.Chr28.1841* | subtilisin-like protease SBT1.4 |
| *AHE.Chr28.1847* | Dual specificity phosphatase |
| *AHE.Chr28.1849* | #N/A |
| *AHE.Chr28.1850* | zinc finger CCCH domain-containing protein 5 |
| *AHE.Chr28.1852* | Ribonuclease H2, subunit A |
| *AHE.Chr28.1851* | uncharacterized protein LOC112093994 |
| *AHE.Chr28.1853* | uncharacterized protein LOC21401340 |
| *AHE.Chr28.1854* | Synaptobrevin |
| *AHE.Chr28.1873* | uncharacterized protein LOC21403157 |
| *AHE.Chr28.1992* | ethylene-responsive transcription factor WRI1 |
| *AHE.Chr28.2000* | WAT1-related protein At4g19185 isoform X1 |
| *AHE.Chr28.2017* | photosystem II D1 precursor processing protein PSB27-H2, chloroplastic isoform X1 |
| *AHE.Chr28.2018* | imidazoleglycerol-phosphate dehydratase |
| *AHE.Chr28.2019* | Chromodomain-helicase-DNA-binding protein Mi-2-like protein |
| *AHE.Chr28.2028* | cyclic dof factor 2 |
| *AHE.Chr28.2033* | protein RBL isoform X1 |
| *AHE.Chr28.2041* | sucrose-phosphatase 2 isoform X1 |
| *AHE.Chr28.2043.1* | ubiquitin carboxyl-terminal hydrolase 12 |
| *AHE.Chr28.2045* | ABC transporter I family member 6, chloroplastic isoform X2 |
| *AHE.Chr28.2067* | SUN domain-containing protein 1 |
| *AHE.Chr28.2107* | WAT1-related protein At5g07050 isoform X1 |
| *AHE.Chr28.2109* | importin-11 |
| *AHE.Chr28.2114* | ABC transporter D family member 2, chloroplastic |
| *AHE.Chr28.2116* | zinc finger CCCH domain-containing protein 37 isoform X1 |
| *AHE.Chr28.2140* | E3 ubiquitin-protein ligase KEG |
| *AHE.Chr28.2150.1* | protein FAR1-RELATED SEQUENCE 3-like |
| *AHE.Chr28.2158* | probable carboxylesterase 15 |
| *AHE.Chr28.2165* | phospholipid-transporting ATPase 1-like isoform X1 |
| *AHE.Chr28.2166* | hypothetical protein ERO13_A05G395600v2 |
| *AHE.Chr28.2167* | phospholipid-transporting ATPase 1-like isoform X1 |
| *AHE.Chr28.2172* | vacuolar protein sorting-associated protein 36 |
| *AHE.Chr28.2178* | heme-binding-like protein At3g10130, chloroplastic |
| *AHE.Chr28.2183* | protein SSUH2 homolog |

**Supplementary Table S20.** KEGG enrichment of the genes under selection between jackfruit and cempedak.

| **ID** | **Description** | ***p*.adjust** | **geneID** | **Count** |
| --- | --- | --- | --- | --- |
| K11000 | callose synthase [EC:2.4.1.-] | 0.003459 | *AHE.Chr11.88/AHE.Chr13.698/AHE.Chr18.898/AHE.Chr19.400/AHE.Chr21.1607/AHE.Chr21.1636/AHE.Chr22.1848/AHE.Chr22.1912* | 8 |

**Supplementary Table S21.** The genes under selection of firm flesh type of jackfruit

| **Gene ID** | **Annotation** |
| --- | --- |
| *AHE.Chr24.1* | PREDICTED: aminoacyl tRNA synthase complex-interacting multifunctional protein 1-like |
| *AHE.Chr24.2* | #N/A |
| *AHE.Chr24.4.2* | desiccation-related protein At2g46140-like |
| *AHE.Chr24.6* | inactive glucose-1-phosphate adenylyltransferase small subunit 2, chloroplastic |
| *AHE.Chr24.7* | Ribosomal protein |
| *AHE.Chr24.8* | thioredoxin-like fold domain-containing protein MRL7L, chloroplastic |
| *AHE.Chr24.9* | hypothetical protein L484_016814 |
| *AHE.Chr24.10* | uncharacterized protein LOC21394645 |
| *AHE.Chr24.11* | #N/A |
| *AHE.Chr24.12* | probable uridine nucleosidase 2 |
| *AHE.Chr24.13* | la-related protein 6B |
| *AHE.Chr24.14.1* | outer envelope pore protein 37, chloroplastic |
| *AHE.Chr24.15* | uncharacterized protein LOC21403248 |
| *AHE.Chr24.16* | general transcription factor 3C polypeptide 5 isoform X1 |
| *AHE.Chr24.17* | probable thiol methyltransferase 2 isoform X2 |
| *AHE.Chr24.20* | type I inositol polyphosphate 5-phosphatase 13 isoform X1 |
| *AHE.Chr24.21* | ankyrin repeat-containing protein At5g02620 |
| *AHE.Chr24.22* | polygalacturonase |
| *AHE.Chr24.25* | transmembrane protein 147 |
| *AHE.Chr24.26* | polygalacturonase |
| *AHE.Chr24.27* | ADH_zinc_N domain-containing protein |
| *AHE.Chr24.28* | 2-alkenal reductase (NADP(+)-dependent) |
| *AHE.Chr24.29* | acetolactate synthase small subunit 2, chloroplastic |
| *AHE.Chr24.30* | Dual specificity protein kinase shkC |
| *AHE.Chr24.31* | mitochondrial proton/calcium exchanger protein |
| *AHE.Chr24.32* | UDP-glycosyltransferase 74F2 |
| *AHE.Chr24.33* | UDP-glycosyltransferase 74F2 |
| *AHE.Chr24.34* | UDP-glycosyltransferase 74F2 |
| *AHE.Chr24.37* | BTB/POZ and TAZ domain-containing protein 3 |
| *AHE.Chr24.38* | acyl-coenzyme A thioesterase 9, mitochondrial |
| *AHE.Chr24.39* | formin-like protein 2 |
| *AHE.Chr24.40* | protein FAM133 |
| *AHE.Chr24.41* | mitogen-activated protein kinase homolog MMK1 |
| *AHE.Chr24.42* | uncharacterized protein LOC21403279 isoform X1 |
| *AHE.Chr24.43* | #N/A |
| *AHE.Chr24.44* | 60S ribosomal protein L5 |
| *AHE.Chr24.45* | probable 2-oxoglutarate-dependent dioxygenase AOP1 |
| *AHE.Chr24.46* | Probably inactive leucine-rich repeat receptor-like protein kinase |
| *AHE.Chr24.47* | Probably inactive leucine-rich repeat receptor-like protein kinase |
| *AHE.Chr24.48* | probable phosphoinositide phosphatase SAC9 |
| *AHE.Chr24.49* | probably inactive leucine-rich repeat receptor-like protein kinase At5g48380 |
| *AHE.Chr24.50* | uncharacterized protein LOC112092503 |
| *AHE.Chr24.51.1* | ion channel CASTOR isoform X2 |
| *AHE.Chr24.52* | U5 small nuclear ribonucleoprotein 40 kDa protein |
| *AHE.Chr24.53* | cysteine synthase |
| *AHE.Chr24.54* | (+)-neomenthol dehydrogenase isoform X1 |
| *AHE.Chr24.56* | uncharacterized protein LOC21403287 |
| *AHE.Chr24.57* | protein FAM136A |

**Supplementary Table S22.** KEGG enrichment of the genes in Chr24-500-kb region under selection between firm flesh type and soft flesh type of jackfruit.

| **ID** | **Description** | ***p*.adjust** | **geneID** | **Count** |
| --- | --- | --- | --- | --- |
| K13691 | pathogen-inducible salicylic acid glucosyltransferase [EC:2.4.1.-] | 1.77E-05 | *AHE.Chr24.32/AHE.Chr24.33/AHE.Chr24.34* | 3 |
| K07119 | uncharacterized protein | 0.001429 | *AHE.Chr24.27/AHE.Chr24.28* | 2 |
| K01099 | phosphatidylinositol-bisphosphatase [EC:3.1.3.36] | 0.002746 | *AHE.Chr24.20/AHE.Chr24.47* | 2 |
| K01184 | polygalacturonase [EC:3.2.1.15] | 0.002746 | *AHE.Chr24.22/AHE.Chr24.26* | 2 |
| K00975 | glucose-1-phosphate adenylyltransferase [EC:2.7.7.27] | 0.02243 | *AHE.Chr24.6* | 1 |
| K06129 | lysophospholipase III [EC:3.1.1.5] | 0.02243 | *AHE.Chr24.25* | 1 |
| K00079 | carbonyl reductase 1 [EC:1.1.1.184 1.1.1.189 1.1.1.197] | 0.02243 | *AHE.Chr24.54* | 1 |
| K15503 | serine/threonine-protein phosphatase 6 regulatory ankyrin repeat subunit B | 0.02243 | *AHE.Chr24.21* | 1 |
| K01738 | cysteine synthase A [EC:2.5.1.47] | 0.024881 | *AHE.Chr24.53* | 1 |
| K04124 | gibberellin 3-beta-dioxygenase [EC:1.14.11.15] | 0.031244 | *AHE.Chr24.45* | 1 |
| K02184 | formin 2 | 0.044336 | *AHE.Chr24.39* | 1 |
| K03695 | ATP-dependent Clp protease ATP-binding subunit ClpB | 0.046668 | *AHE.Chr24.51.1* | 1 |

**Supplementary Table S23.** Genes related to pectin degradation of jackfruit.

| **Gene ID** | **Abbreviation** | **Annotation** |
| --- | --- | --- |
| *AHE.Chr26.1408* | *AFase1* | LOW QUALITY PROTEIN: alpha-L-arabinofuranosidase 1 |
| ***AHE.Chr24.26*** | *AhePG1* | polygalacturonase |
| *AHE.Chr01.149* | *Gal1* | beta-galactosidase 10 isoform X1 |
| *AHE.Chr22.360* | *Gal10* | beta-galactosidase |
| *AHE.Chr23.544* | *Gal11* | beta-galactosidase 1 |
| *AHE.Chr24.607* | *Gal12* | beta-galactosidase 17 |
| *AHE.Chr24.622* | *Gal13* | beta-galactosidase 1 |
| *AHE.Chr28.743* | *Gal14* | alpha-galactosidase 3 |
| *AHE.fragScaff_scaffold_206_pilon.26* | *Gal15* | beta-galactosidase 1 |
| *AHE.Chr01.1196* | *Gal2* | beta-galactosidase 3 |
| *AHE.Chr01.1277* | *Gal3* | beta-galactosidase 3 |
| *AHE.Chr02.1026* | *Gal4* | beta-galactosidase 3 |
| *AHE.Chr03.409* | *Gal5* | beta-galactosidase |
| *AHE.Chr04.461* | *Gal6* | beta-galactosidase |
| *AHE.Chr12.697* | *Gal7* | beta-galactosidase 8 |
| *AHE.Chr15.1201* | *Gal8* | beta-galactosidase 9 isoform X1 |
| *AHE.Chr19.1436* | *Gal9* | alpha-galactosidase |
| *AHE.Chr19.836* | *PE1* | pectinesterase 1 |
| *AHE.Chr22.657* | *PE2* | pectinesterase 31 isoform X1 |
| *AHE.Chr23.417* | *PE3* | pectinesterase |
| *AHE.Chr24.231* | *PE4* | pectinesterase |
| *AHE.original_scaffold_1148_pilon.11* | *PE5* | pectinesterase 31 isoform X1 |
| *AHE.original_scaffold_593_pilon.51* | *PE6* | pectinesterase |
| *AHE.Chr16.189* | *PG10* | probable polygalacturonase |
| *AHE.Chr21.536* | *PG11* | polygalacturonase At1g48100 isoform X2 |
| *AHE.Chr25.1398* | *PG12* | polygalacturonase |
| *AHE.Chr26.835* | *PG13* | PREDICTED: polygalacturonase |
| *AHE.Chr28.456* | *PG14* | probable polygalacturonase |
| *AHE.Chr28.458* | *PG15* | probable polygalacturonase |
| *AHE.original_scaffold_676_pilon.59* | *PG16* | probable polygalacturonase |
| *AHE.Chr03.550* | *PG3* | probable polygalacturonase |
| *AHE.Chr03.1134* | *PG4* | LOW QUALITY PROTEIN: probable polygalacturonase |
| *AHE.Chr04.613* | *PG5* | probable polygalacturonase |
| *AHE.Chr04.816* | *PG6* | exopolygalacturonase |
| *AHE.Chr09.123* | *PG7* | probable polygalacturonase |
| *AHE.Chr13.1118* | *PG8* | polygalacturonase 1 beta-like protein 3 |
| *AHE.Chr15.164* | *PG9* | probable polygalacturonase |

| **Gene ID** | **Abbreviation** | **Annotation** |
| --- | --- | --- |
| *AHE.Chr15.995* | *PL1* | Pectate lyase |
| *AHE.Chr28.1920* | *PL2* | probable pectate lyase 18 |
| *AHE.Chr01.57* | */* | probable polygalacturonase |
| *AHE.Chr01.1244* | */* | probable pectinesterase 53 |
| *AHE.Chr02.38* | */* | beta-galactosidase 10 isoform X2 |
| *AHE.Chr02.1074* | */* | probable pectinesterase 53 |
| *AHE.Chr02.1075* | */* | probable pectinesterase 53 |
| *AHE.Chr03.47* | */* | putative polygalacturonase |
| *AHE.Chr03.321* | */* | probable pectinesterase 53 |
| *AHE.Chr03.411* | */* | beta-galactosidase 1 |
| *AHE.Chr03.601* | */* | pectinesterase isoform X1 |
| *AHE.Chr03.616* | */* | Pectinesterase, Tyr active site |
| *AHE.Chr03.811* | */* | pectinesterase 2 |
| *AHE.Chr03.953* | */* | beta-galactosidase 8 |
| *AHE.Chr04.459* | */* | Beta-galactosidase 1 |
| *AHE.Chr04.634* | */* | Pectinesterase, Tyr active site |
| *AHE.Chr04.648* | */* | pectinesterase isoform X1 |
| *AHE.Chr04.1152* | */* | pectinesterase 2 |
| *AHE.Chr04.1199* | */* | exopolygalacturonase |
| *AHE.Chr04.1378* | */* | LOW QUALITY PROTEIN: probable polygalacturonase |
| *AHE.Chr05.17* | */* | exopolygalacturonase |
| *AHE.Chr05.589* | */* | exopolygalacturonase |
| *AHE.Chr05.1635* | */* | pectinesterase QRT1 |
| *AHE.Chr06.1372* | */* | pectinesterase QRT1 |
| *AHE.Chr07.350* | */* | beta-galactosidase 3 isoform X1 |
| *AHE.Chr07.436* | */* | putative pectinesterase 11 |
| *AHE.Chr07.1094* | */* | beta-galactosidase 16 |
| *AHE.Chr08.257* | */* | beta-galactosidase 3 isoform X1 |
| *AHE.Chr08.306* | */* | putative pectinesterase 11 |
| *AHE.Chr08.436* | */* | polygalacturonase-like |
| *AHE.Chr08.959* | */* | beta-galactosidase 16 |
| *AHE.Chr08.1341* | */* | probable polygalacturonase At3g15720 |
| *AHE.Chr08.1358* | */* | probable polygalacturonase At3g15720 |
| *AHE.Chr08.1926* | */* | putative pectinesterase 63 |
| *AHE.Chr09.91* | */* | polygalacturonase At1g48100 |
| *AHE.Chr09.354* | */* | beta-galactosidase 11 |
| *AHE.Chr09.724* | */* | probable pectate lyase 8 |
| *AHE.Chr09.1206* | */* | exopolygalacturonase |
| *AHE.Chr09.1593* | */* | putative pectinesterase 11 |

| **Gene ID** | **Abbreviation** | **Annotation** |
| --- | --- | --- |
| *AHE.Chr10.269* | */* | beta-galactosidase 11 |
| *AHE.Chr10.651* | */* | probable pectate lyase 8 |
| *AHE.Chr10.1498* | */* | beta-galactosidase 3 |
| *AHE.Chr10.1865* | */* | beta-xylosidase/alpha-L-arabinofuranosidase 2 |
| *AHE.Chr11.383* | */* | polygalacturonase At1g48100 |
| *AHE.Chr12.1367* | */* | Putative pectinesterase 10 |
| *AHE.Chr12.1408* | */* | Polygalacturonase |
| *AHE.Chr12.1500* | */* | Pectinesterase-2 precursor |
| *AHE.Chr12.2258* | */* | polygalacturonase |
| *AHE.Chr13.199* | */* | pectinesterase |
| *AHE.Chr13.1021* | */* | pectate lyase |
| *AHE.Chr13.1117* | */* | polygalacturonase 1 beta-like protein 3 |
| *AHE.Chr13.1119* | */* | polygalacturonase 1 beta-like protein 3 |
| *AHE.Chr13.1141* | */* | polygalacturonase At1g48100 |
| *AHE.Chr13.1142* | */* | probable polygalacturonase At1g80170 |
| *AHE.Chr13.1169* | */* | polygalacturonase At1g48100 |
| *AHE.Chr13.1269* | */* | Pectate lyase |
| *AHE.Chr14.248* | */* | Pectate lyase |
| *AHE.Chr14.282* | */* | putative alpha-galactosidase |
| *AHE.Chr14.319* | */* | Pectate lyase |
| *AHE.Chr14.476* | */* | pectate lyase |
| *AHE.Chr14.1177* | */* | pectinesterase |
| *AHE.Chr15.10* | */* | Pectinesterase 3 |
| *AHE.Chr15.76* | */* | polygalacturonase At1g48100 |
| *AHE.Chr15.243* | */* | probable pectinesterase 67 |
| *AHE.Chr15.244* | */* | probable pectinesterase 67 |
| *AHE.Chr15.1596* | */* | alpha-galactosidase 1 |
| *AHE.Chr15.1630* | */* | Beta-galactosidase 6 |
| *AHE.Chr15.1631* | */* | Beta-galactosidase 6 |
| *AHE.Chr15.1632* | */* | Beta-galactosidase 6 |
| *AHE.Chr16.32* | */* | probable pectinesterase 67 |
| *AHE.Chr16.88* | */* | polygalacturonase At1g48100 |
| *AHE.Chr16.581* | */* | exopolygalacturonase |
| *AHE.Chr16.1006* | */* | probable pectate lyase 1 |
| *AHE.Chr16.1076* | */* | probable pectinesterase 29 |
| *AHE.Chr16.1213* | */* | beta-galactosidase 9 isoform X1 |
| *AHE.Chr17.1295* | */* | probable polygalacturonase |
| *AHE.Chr17.1296* | */* | probable polygalacturonase |
| *AHE.Chr17.1320* | */* | probable polygalacturonase |
| *AHE.Chr17.1321* | */* | probable polygalacturonase |

| **Gene ID** | **Abbreviation** | **Annotation** |  |
| --- | --- | --- | --- |
| *AHE.Chr17.1652* | */* | probable pectinesterase 68 | |
| *AHE.Chr18.82* | */* | probable pectinesterase 68 | |
| *AHE.Chr18.463* | */* | probable polygalacturonase, partial | |
| *AHE.Chr19.54* | */* | polygalacturonase QRT2 | |
| *AHE.Chr19.58* | */* | pectinesterase 2 | |
| *AHE.Chr19.426* | */* | probable pectate lyase 18 | |
| *AHE.Chr19.427* | */* | pectate lyase 22 | |
| *AHE.Chr20.401* | */* | probable pectate lyase 18 | |
| *AHE.Chr20.586* | */* | polygalacturonase 1 beta-like protein 1 | |
| *AHE.Chr21.142* | */* | pectate lyase | |
| *AHE.Chr21.255* | */* | pectinesterase | |
| *AHE.Chr21.277* | */* | polygalacturonase At1g48100 | |
| *AHE.Chr21.295* | */* | probable pectinesterase 53 | |
| *AHE.Chr21.324* | */* | probable pectate lyase 19 | |
| *AHE.Chr21.554* | */* | beta-galactosidase | |
| *AHE.Chr21.800* | */* | pectinesterase 31 isoform X1 | |
| *AHE.Chr21.1316* | */* | probable pectate lyase 9 | |
| *AHE.Chr21.1332* | */* | probable pectate lyase 9 | |
| *AHE.Chr21.1480* | */* | probable pectinesterase 8 | |
| *AHE.Chr21.1820* | */* | exopolygalacturonase | |
| *AHE.Chr22.238* | */* | pectate lyase | |
| *AHE.Chr22.341* | */* | polygalacturonase At1g48100 isoform X2 | |
| *AHE.Chr22.412* | */* | polygalacturonase | |
| *AHE.Chr22.518* | */* | probable pectate lyase 19 | |
| *AHE.Chr22.1394* | */* | exopolygalacturonase | |
| *AHE.Chr22.1395* | */* | exopolygalacturonase | |
| *AHE.Chr22.2194* | */* | probable pectinesterase 8 | |
| *AHE.Chr23.129* | */* | polygalacturonase | |
| *AHE.Chr23.203* | */* | putative pectate lyase 2 | |
| *AHE.Chr23.233* | */* | putative pectate lyase 2 | |
| *AHE.Chr23.1146* | */* | alpha-galactosidase | |
| *AHE.Chr23.2049* | */* | polygalacturonase QRT3 | |
| *AHE.Chr24.22* | */* | polygalacturonase | |
| *AHE.Chr24.189* | */* | putative pectate lyase 2 | |
| *AHE.Chr24.1174* | */* | alpha-galactosidase | |
| *AHE.Chr24.2131* | */* | polygalacturonase QRT3 | |
| *AHE.Chr24.2141* | */* | polygalacturonase QRT3 | |
| *AHE.Chr25.311* | */* | probable pectate lyase 12 | |
| *AHE.Chr25.342* | */* | putative pectinesterase 15 | |
| *AHE.Chr25.343* | */* | putative pectinesterase 15 | |

| **Gene ID** | **Abbreviation** | **Annotation** |  |
| --- | --- | --- | --- |
| *AHE.Chr25.410* | */* | exopolygalacturonase | |
| *AHE.Chr25.1086* | */* | putative pectinesterase 63 | |
| *AHE.Chr26.286* | */* | probable pectate lyase 12 | |
| *AHE.Chr26.349* | */* | exopolygalacturonase | |
| *AHE.Chr26.951* | */* | polygalacturonase | |
| *AHE.Chr27.69* | */* | putative polygalacturonase | |
| *AHE.Chr27.71* | */* | pectate lyase | |
| *AHE.Chr27.470* | */* | alpha-galactosidase 3 | |
| *AHE.Chr27.596* | */* | putative pectinesterase 63 | |
| *AHE.Chr27.598* | */* | probable pectinesterase 50 | |
| *AHE.Chr27.651* | */* | alpha-galactosidase 3 | |
| *AHE.Chr27.1204* | */* | probable pectate lyase 18 | |
| *AHE.Chr28.460* | */* | pectate lyase | |
| *AHE.Chr28.674* | */* | putative pectinesterase 63 | |
| *AHE.Chr28.677* | */* | probable pectinesterase 50 | |
| *AHE.Chr28.1257* | */* | exopolygalacturonase | |
| *AHE.Chr28.1644* | */* | pectinesterase 2 | |
| *AHE.fragScaff_scaffold_221_pilon.4_AHE.fragScaff_scaffold_221_pilon.5* | */* | polygalacturonase 1 beta-like protein 3 | |
| *AHE.fragScaff_scaffold_221_pilon.6* | */* | olygalacturonase 1 beta-like protein 3 | |
| *AHE.fragScaff_scaffold_221_pilon.7* | */* | polygalacturonase 1 beta-like protein 3 | |
| *AHE.fragScaff_scaffold_221_pilon.32* | */* | polygalacturonase At1g48100 | |
| *AHE.fragScaff_scaffold_221_pilon.33* | */* | putative polygalacturonase | |
| *AHE.fragScaff_scaffold_82_pilon.2* | */* | alpha-galactosidase | |
| *AHE.original_scaffold_490_pilon.37* | */* | probable pectinesterase 50 | |
| *AHE.original_scaffold_545_pilon.33* | */* | ative pectinesterase 10 | |
| *AHE.original_scaffold_545_pilon.35* | */* | probable pectinesterase 29 | |
| *AHE.original_scaffold_622_pilon.7* | */* | polygalacturonase QRT2 | |
| *AHE.original_scaffold_622_pilon.8* | */* | probable pectinesterase 56 | |
| *AHE.original_scaffold_622_pilon.10* | */* | probable pectinesterase 56 | |
| *AHE.original_scaffold_679_pilon.42* | */* | probable pectinesterase 55 | |
| *AHE.original_scaffold_921_pilon.27* | */* | probable pectinesterase 56 | |
| *AHE.original_scaffold_921_pilon.29* | */* | probable pectinesterase 56 | |
| *AHE.original_scaffold_921_pilon.30* | */* | polygalacturonase QRT2 | |
| *AHE.original_scaffold_960_pilon.2* | */* | Pectinesterase, Tyr active site | |
